# Supplementary material for: Exploration of the Potential Transcriptional Regulatory Mechanisms of DNA Methyltransferases and MBD Genes in Petunia Anther Development and Multi-Stress Responses
Source: Genes (Basel). 2022 Feb 8;13(2):314. doi: 10.3390/genes13020314 (PMC8872020; doi:10.3390/genes13020314)
Supplement: Supplementary file 1 [file genes-13-00314-s001.zip › Figure S2.pdf]

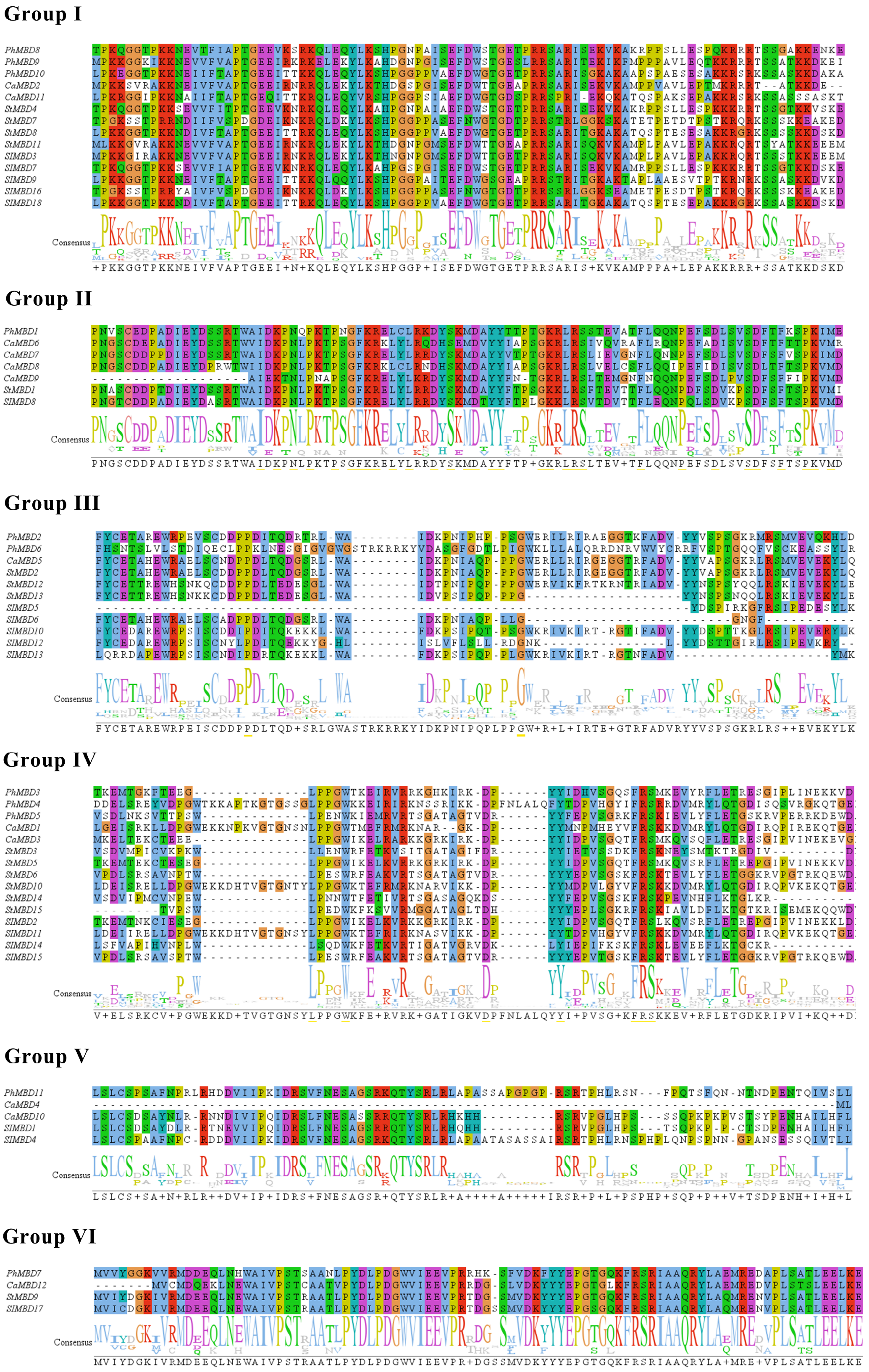

Figure S2. Multiple-sequence alignments of *MBD* genes

The conserved MBD domains of *PhMBDs*, *CaMBDs*, *StC5-MTases* and *SlC5-MTases*.
