# Supplementary material for: Exploration of the Potential Transcriptional Regulatory Mechanisms of DNA Methyltransferases and MBD Genes in Petunia Anther Development and Multi-Stress Responses
Source: Genes (Basel). 2022 Feb 8;13(2):314. doi: 10.3390/genes13020314 (PMC8872020; doi:10.3390/genes13020314)
Supplement: Supplementary file 1 [file genes-13-00314-s001.zip › Table S1.pdf]

Table S1. Sequences of C5-MTase proteins in the 15 tested species.

>SmelMET1
MGSLPALDKPDTDAGYKKNKTKQDSQSKRKTSATDKKEKKQPVSESIEELTAARKRPKRA
AACSDFKEKSLPLSKTSSVIETRKDHCVEEEDVAIRLTAGLQESQRPCRRLTDFVFHNSEGI
PQPFEMSEVDDLFISGLILPLEDSLDKEKAKGIRCEGFGRIEEWAISGYEDGTPVIWISTETA
DYDCVKPSGSYKKFYDHFLAKAMACVEVYKKLSKSSGGNPNLSLDELLAGVVRAMNGI
KCFSGGVSIRDFVVTQGRFIYNQLIGLDETSKKTDQLFVELPVLASLRDESSKLETLAQPEP
ISFGKALHIGPKAVNGEDKIDESGLANCAASEDEDLKLAKLLHEEEYWRSLKQKKGRNTS
SSSSKIYIKINEDEIASDYPLPAYYKTSNEETDEYIVFDSGVDTYHIDELPRSMLHNWALYNS
DSRLISLELLPMKACADIDVTIFGSGVMTADDGSGYNFDTDANHSSSCGSRPAEIDGMPIY
LSAIKEWMIEFGSSMIFISIRTDMAWYRLGKPSKQYAPWYEPVIKTARLAVSIITLLKEQSRV
ARLSFGDVIKRISEFKKDRPAYISSNVDVVERYVVVHGQIILQQFSEFPDVSIRNCAFAIGLS
RKMEERHHTKWLIKKKKVMQRHEQNLNPRASMAPSVKRKAMQATTTRLINRIWGEYYS
NYSPEVSKEVVDCEVNDDEEADEQEENEEDDVPEMNLDVPEKTHTLSSARKHIKSHSDS
KEINWDGESTGKTASGELLFERARVHGHEIAVGDSVLVEHDEPDELPSIYFVEYMFEKLDG
SKMLHGRMMQRGSDTVLGNAANEREVFLINECMDLQLGDIKESIAVNIRIMPWGHQHRN
TNADKLDRAKAEDRKRKGLPTEFYCKSFYRPERGAFFRLPFDKMGLGNGLCYSCELQRT
DQEKESFKFDMSNSSFVYLGTEYSVDDFVYVSPDHFTEERGGTGTFKAGRNVGLMAYVV
CQLLEIVGPKGSKQAKVDSTNAKVRRFFRPEDISSDKAYSSDIREIYYSEEIHTVSVGTIKG
KCEVRKKYDISSEDVPAIFDHNFFCEYLYDPSNGSLKKLPAQIKLKFSKVKLDDATSRKRK
GKGKEGEDEAGGLNEASPQNRLATLDIFAGCGGLSEGLQHSGVTDTKWAIEYEEPAADAF
RLNHPKTKVFIHNCNVILRAVMQKCGDSDDCISTPEASELAAAMDENELNSLPLPGQVDFI
NGGPPCQGFSGMNRFNQSTWSKVQCEMILAFLSFADYYRPKFFLLENVRNFVSFNQKQTF
RLSVASLLEMGYQVRFGILEAGAFGVPQSRKRAFIWAASPEEVLPEWPEPMHVFSVPELKI
TLSETSHYAAVRSTASGAPFRSLTVRDTIGDLPVVGNGACKTCIEYQGDPVSWFQKKIRGS
SITLSDHISKEMNELNLIRCQRIPKRPGADWRDLQDEKVKLSNGQLVDLIPWCLPNTAKRH
NQWKGLFGRLDWDGNFPTSITDPQPMGKVGMCFHPEQDRIVTVRECARSQGFPDSYQFA
GNILQKHRQIGNAVPPPLAYALGRKLKEAVESKKLT
>SmelDNMT2
MELEGSRSPWRVLEFYSGIGGLRYSLLKAAVNATVVEAFDINDVANDVYQHNFAHRPFQG
NIQTLTAADLDGYNADAWLLSPPCQPYTRQGLQKGSSDARASSFLKILELVPQLLQPPSY
LFVENVVGFETSDTHAILVEILKKNNFVTQEFILSPLQFGLPYSRPRYYCLPPDFGVMVR
RQCVMYRLSVHVKFEHLGRPKPEAEKGSVLVALSILSFENMHVLSYPYAKRKPLSFEVPE
FNNQLLRTPGPLLGQTESTMEKEQLLSPEYWDELLQACQPVDDFLVFKTFHNGKDSSTYS
FHSNDSVKSDRSDEENNVYFVPSSLIERWGSAMDIVYPHSKRCCCFTKSYYRYVKGTGSL
LASVQTLFSRNHFFVVYFEELPKIGAIPAMACISHPSDNIIYYNAGFKSYKITRTAGTES
WFEWTEYSRNLMRKVQISLKVLKWLVSVLIEASKCRGNIIKRWSLKDHFEEFYGTLKYNE
SGRYVSFIAIQGKNRSIIITSKPSHGGGWSNIAHKIAGFIYETNCRTEEKNYGGRKANMS
YKQAVQRSRWSTEKVKTTDLQGIDTHIDVTNGATLGKSELLSRCLVRTVLKSTARKPNP
>SmelCMT3a
MSTKRKASPAESPESKRHAVTENADQVVEEPTEDFLVECFVDEETVFDGLNDGSHAEKKE
RRVVQVNEEQEGVFSGETVRDSEAREKWPHRYIKDKVKANGTPMSLNCQDGSNQLIQA
KCHYTQAMVDSQIYKLGDDAYVKAADGEDDYICKIVEFFQAVDDMKYFTAQWFYRAKD

TVIKAHDQFIDNKRVFLSEIKDDNPLDCLVSLQFKESLRSNSDYYYDMKYLVPFSSFVSLSS
DFRDSAVFADVSSPYSESDSTISTDGDVVEANEQKQEKKLLDLYSGCGGMSTGLCLGADV
CGVKLVTKWTVDLNRYACDSLKVNHPETEVRNESASDYLLLLKEWEQLCASCSLLKSNT
PAHPLLKVGDEDEEDDDDGGDEDEGSGDNEEGEIFEVEEILEVCYGDPNEIKKPGLYFKVR
WKGYGPDEDTWEPIEGLEYEFQTKQHVVNPCFSFLTHLSNELIFAYSGCQKKIKDFVIKGF
QASLFPLPGEVDVICGGPPCQGISGFNRFRNSANPLQDPKNKQLEVFMGIVEFLKPRFVLM
ENVVDLLRFANGYLGRYALSRLVGMNYQARMGMMVAGAYGLPQFRMRVFMWGALPSE
KLPQYPLPTHNVIVRGGIPTEFELNAVEYEEGLQVKLKRELLLEDALSDLPPVENNEPRDE
MPYTGEPKSDFQCFIRSKRDGTLGTVLYDHRPLQLNDDDYQRVCQIPKRKGGNFRDLPGV
RVRADNIVEWDPDVERVKLTSGKPLVPDYAMTFVRGTSQKPFGRLWWDEIVSTVVTRAEP
HNQAILHPVQDRVLTIRENARLQGFPDYYKLTGPIKERYIQVGNAVAVPVARALGYSLALA
LKGLSGDQPLLTLPPNFPRLEELVSNEEALDKL

>SmelCMT2
METELNNSSKISENQTPSPEKTTPISSSSPNPDALALSFSLNEEPVPLVVCYPGRRRSSRFST
NKFSTSELMKKNRKCISPEKEVLLPSALAGKKTPAESTRRSPRLVSPSATGKVKGKGKNVN
SRKQVESSRKRKYKNNDDEPENPKTDGKKRRSDSVSAKLELLALPEADGAWGTRCGTKR
ELLALLTPEGSKNSKRGSRGSDSGNVGHDSRGSRRKNLVLAASPGTSEKKSDVVNSVGEK
SLRSRRIQGSVDNEESDTKRKMNSSDSAEESGRKQKSNVFFIGEPIDAEEAQEKWQWRYE
LKSQNTQRKGWKLNSGEEDEIILNVECHYAQAKVAGFVFNIGDCAFVKGEGKKKHIGRIL
EFFKTTEGEDYFRVQWYFRAEDTVLKGAASFHDPKRIFYSTLENDNLLDCIVSKVNVIELP
TAHALNKKDVPPAHFYYDMEYCVDYSTFRTLHNVKSSISPSLVDASYKPITTYPLEVSPSC
EPMKVELSLLDLYAGCGGMSTGLCLGTKLSGVNLVTKWAVDFNKSACDSLKLNHPQTHV
RNEGVENFLELLKRWEKLIELYGCSDIKTSSNDAVDDRDEGEINGDSLAGSDASSGEYEVL
RLVDICYGDPNNDEKSGLHFKVRWKGYGPSEDTWEPIDNLENCGESIKDFVRRGQQLKIL
PLPGDVDMICGGPPCQGISGYNRHRNVDDPLSDEKNRQIIVFMDVVEFLRPKYVLMENVA
DILRFDKASLGRYALSRLVHMKYQARLGTMAAGCYGLPQFRLRVFFWGALPSERLPPFPL
PSHDVIVKYWPSPEFERNTVAYEEGQPRDLEEALVLRDAISDLPAVTWHETREEMPYEMPP
ETAFQKYIRLSKHEIMSCSSTGVKEAKEPVLFDHRPCQLNEDDYFRVCLVPHRKGANFRD
LPGVIVGGDNVARRDTKDPKVLPNGKPMVPDCAFNFEQGKSKRPFGRLWWDETVATLVT
FPNHRAQAILHPEQDRVLTVREYARLQGFPDFYRFTGTPKERYCQVGNAVAVPVGRALGY
ALGLAYQRLAGNEPLIKLPPNFSFLTVPMDDIVVLQK

>SmelCMT3b
MPSKRKASPATKPESSSGSRKSKRLAVEKPDPVVAQPSDSDFESAPPVLSAKKKSTRGTTA
ESPVVASQSVSNDKKLNKRVVEKAESGVASPADTDFVSEPNLVTPGKRSGRGAAVKAEPV
VDSAAESDFVDEEDVDETVQGSLKKSLSISPSKRKPKRAEKVKDEECTLPGDPVPDAEAR
LKWPHRYNKGKANGTKNTNGQDDSDQLIQAKCHFGRAKVDGQVYYLEDDAHVKAAD
GDDDYICKIVEFFEAVDGTQYFRAQWFYRAKDTVIKSHDQFIDNKRVFLSEIKDDNPIDCL
VTKLKIVPVPSNATLQFKENVKSNCDFYYDMKYLLPYSSFISLPPDTTSPVSSSSTISSDVD
AGEVKEHNPEKKLLDLYSGCGAMSTGLCLGANSNGVKLVTKWAVDLNKHACDSLRLNH
PETQVRNEYASDFLSLLKEWVQLCVSCSLIKGSVPPHPHLKVTDEVEEDEENDDEGEDSG
DDKDGENFEVEELLEVCYGDPKEINKSGLYFKVHWKGYGPEEDTWEPIDGLSDCPKKIKE
FVVKGFKANLLPLPGDVDVVCGGPPCQGISGFNRFRNKENPMQDPKNKQLDVYMDIVDF

LKPRFVLMENVVDLVKFSNGFLGRYALSRLVGMNYQARMGMMAAGAYGLPQFRMRVF
MFGALSSEKLPQYPLPTHKVIVRGVIPTEFESNTVAYDEGRDFELKKELFLGDALSDLPSVE
NNEPRDEMPYADEPKSDFQHFIRLGRDGVLGSVLYDHRPLQLNDDDYQRVCQIPKRKGA
NFRDLPGVRVRPDKKVEWDPDVERVKLPSGKPLVPDYAMSFVGGSSSKPFGRLWWDETV
PTVVTRAEPHNQTIIHPLQDRVLTIRENARLQGFPDYYKLIGPIKERYMQVGNAVAVPVARA
LGYSLAMSMKGLSGETPLFSLPQNFPFHEEQNCNEVSQ

>SmelDRM2
MDKNLSEEDSDNIDWDTEDELEIQDTTFSSCRDLRTTGQYAITGDGEASSSSASGQSKFIQ
KFVVMGFPEESIAKAIEQNGENSDLVLDALLTLKAIEDSPEEQPSASPHLEPCINSDDSSSEY
NENFLDDVYEEDSWSSDSDFCLNSAKQCYLKEESSSLSEKEQTLLFLQNMGYPVEEVSIA
MERCGPEASVAELTDFICAAQMARAEDPYLPEDVKPKINGNGGFKKRKMFNQLCKSKKP
RAIFDEETIRLPKPMIGFGVPTEPVSAIVHRTLPEQAVGPPFFYYENVALAPKGVWDTISRFL
YDIEPEFVDSKYFCAAARKRGYIHNLPIENRFPLLPLPPRTINEALPLTKKWWPSWDPRTKL
NCLQTAIGSARLTDRIRKAVEAFDGEPPMRVQKYVLDQCRKWNLVWVGRNKVAPLEPDE
FEMLLGFPKNHTRGGGISRTDRYKSLGNSFQVDTVAYHLSVLKDRFPNGMNVLSLFSGIG
GAEVALYRLGIQLNNVVSVEKSEVNRNIVRSWWEQTNQRGNLIDFEDVQQLNGDRLEQLI
DSFGGFDLLIGGSPCNNLAGSNRVSRDGLEGKESSLFYDYVRILDLVKSIMARQR

>SmelDRM3
MCEYSDGENSSKPEGVSGIMPKLEDPFGEFPSLYTYTTPIGANIASSSSSNVRSSLLTMGFE
ASLVDKAIEENGEDNIDLLLETLIANSDPPRAESSDSLDKLFGDDEDTNSSAKYDGDVHIK
EEPDPCIGVCDDKRVSLLAMSFSLNEVEFAIGKLGEAAPVNELVDVIFAARIAGNHKKDDD
DDVSVVEIKESKKECTTEALFGTMEKTLKLLEMGFSDNEVSKVIEKFGSEVPLEELANLIV
DPSSGRRMNKHLLNPLGRNSSIGFHPVAVKKEEYSLDTSESREIDLLEKLKGKRPQESYIDE
RDTFKRPKSEYDEAFNNSLGPDWKEILGINNIRPKVCRRAIQQKSRVLDCQDKQKLSMPN
PCRSLDKMVAKAPYFFYGNVMNLSHDSWVRISQFLYSIEPEFVHTQLFSALSRKEGYVHN
LPTENRFHIVPKPPMTIREAVPNSKKWWPSWDTRQHLNCINSETSVVSQICDRLERIVSESQ
GFPSVDRQRDILYQSQIFNLVWVGRYKLAAVGPEQIEHILGYPENHTRLTGTSLMERLLSL
KHCFQIDTLAYCLSTLKHLYPGGLTVLSIYSGIGGAEVALHRLGIRLKAVVSIESSEENRRIL
KQWWSSSGQTGELVQMEDIHKLASNKVELLIKNYGGFDFIICQNPCTYSSKGNLAADIDS
HASLDFMLFHEFVRVMQRVRSTMGRN

>AtCMT1
MAARNKQKKRAEPESDLCFAGKPMSVVESTIRWPHRYQSKKTKLQAPTKKPANKGGKK
EDEEIIKQAKCHFDKALVDGVLINLNDDVYVTGLPGKLKFIAKVIELFEADDGVPYCRFR
WYYRPEDTLIERFSHLVQPKRVFLSNDENDNPLTCIWSKVNIAKVPLPKITSRIEQRVIPPCD
YYYDMKYEVPYLNFTSADDGSDASSSLSSDSALNCFENLHKDEKFLLDLYSGCGAMSTG
FCMGASISGVKLITKWSVDINKFACDSLKLNHPETEVRNEAAEDFLALLKEWKRLCEKFS
LVSSTEPVESISELEDEEVEENDDIDEASTGAELEPGEFEVEKFLGIMFGDPQGTGEKTLQL
MVRWKGYNSSYDTWEPYSGLGNCKEKLKEYVIDGFKSHLLPLPGTVYTVCGGPPCQGIS
GYNRYRNNEAPLEDQKNQQLLVFLDIIDFLKPNYVLMENVVDLLRFSKGFLARHAVASFV
AMNYQTRLGMMAAGSYGLPQLRNRVFLWAAQPSEKLPPYPLPTHEVAKKFNTPKEFKDL
QVGRIQMEFLKLDNALTLADAISDLPPVTNYVANDVMDYNDAAPKTEFENFISLKRSETL

LPAFGGDPTRRLFDHQPLVLGDDDLERVSYIPKQKGANYRDMPGVLVHNNKAEINPRFRA
KLKSGKNVVPAYAISFIKGKSKKPFGRLWGDEIVNTVVTRAEPHNQCVIHPMQNRVLSVR
ENARLQGFPDCYKLCGTIKEKYIQVGNAVAVPVGVALGYAFGMASQGLTDDEPVIKLPFK
YPECMQAKDQI

>AtCMT2
MLSPAKCESEEAQAPLDLHSSSRSEPECLSLVLWCPNPEEAAPSSTRELIKLPDNGEMSLRR
STTLNCNSPEENGGEGRVSQRKSSRGKSQPLLMLTNGCQLRRSPRFRALHANFDNVCSVP
VTKGGVSQRKFSRGKSQPLLTLTNGCQLRRSPRFRAVDGNFDSVCSVPVTGKFGSRKRKS
NSALDKKESSDSEGLTFKDIAVIAKSLEMEIISECQYKNNVAEGRSRLQDPAKRKVDSDTLL
YSSINSSKQSLGSNKRMRRSQRFMKGTENEGEENLGKSKGKGMSLASCSFRRSTRLSGTV
ETGNTETLNRRKDCGPALCGAEQVRGTERLVQISKKDHCCEAMKKCEGDGLVSSKQELL
VFPSGCIKKTVNGCRDRTLGKPRSSGLNTDDIHTSSLKISKNDTSNGLTMTTALVEQDAME
SLLQGKTSACGAADKGKTREMHVNSTVIYLSDSDEPSSIEYLNGDNLTQVESGSALSSGG
NEGIVSLDLNNPTKSTKRKGKRVTRTAVQEQNKRSICFFIGEPLSCEEAQERWRWRYELKE
RKSKSRGQQSEDDEDKIVANVECHYSQAKVDGHTFSLGDFAYIKGEEEETHVGQIVEFFK
TTDGESYFRVQWFYRATDTIMERQATNHDKRRLFYSTVMNDNPVDCLISKVTVLQVSPRV
GLKPNSIKSDYYFDMEYCVEYSTFQTLRNPKTSENKLECCADVVPTESTESILKKKSFSGE
LPVLDLYSGCGGMSTGLSLGAKISGVDVVTKWAVDQNTAACKSLKLNHPNTQVRNDAA
GDFLQLLKEWDKLCKRYVFNNDQRTDTLRSVNSTKETSGSSSSSDDDSDSEEYEVEKLVD
ICFGDHDKTGKNGLKFKVHWKGYRSDEDTWELAEELSNCQDAIREFVTSGFKSKILPLPG
RVGVICGGPPCQGISGYNRHRNVDSPLNDERNQQIIVFMDIVEYLKPSYVLMENVVDILR
MDKGSLGRYALSRLVNMRYQARLGIMTAGCYGLSQFRSRVFMWGAVPNKNLPPFPLPTH
DVIVRYGLPLEFERNVVAYAEGQPRKLEKALVLKDAISDLPHVSNDEDREKLPYESLPKTD
FQRYIRSTKRDLTGSAIDNCNKRTMLLHDHRPFHINEDDYARVCQIPKRKGANFRDLPGLI
VRNNTVCRDPSMEPVILPSGKPLVPGYVFTFQQGKSKRPFARLWWDETVPTVLTVPTCHS
QALLHPEQDRVLTIRESARLQGFPDYFQFCGTIKERYCQIGNAVAVSVSRALGYSLGMAFR
GLARDEHLIKLPQNFSHSTYPQLQETIPH

>AtCMT3
MAPKRKRPATKDDTTKSIPKPKKRAPKRAKTVKEEPVTVVEEGEKHVARFLDEPIPESEAK
STWPDRYKPIEVQPPKASSRKKTKDDEKVEIIRARCHYRRAIVDERQIYELNDDAYVQSGE
GKDPFICKIIEMFEGANGKLYFTARWFYRPSDTVMKEFEILIKKKRVFFSEIQDTNELGLLE
KKLNILMIPLNENTKETIPATENCDFFCDMNYFLPYDTFEAIQQETMMAISESSTISSDTDIR
EGAAAISEIGECSQETEGHKKATLLDLYSGCGAMSTGLCMGAQLSGLNLVTKWAVDMNA
HACKSLQHNHPETNVRNMTAEDFLFLLKEWEKLCIHFSLRNSPNSEEYANLHGLNNVEDN
EDVSEESENEDDGEVFTVDKIVGISFGVPKKLLKRGLYLKVRWLNYDDSHDTWEPIEGLS
NCRGKIEEFVKLGYKSGILPLPGGVDVVCGGPPCQGISGHNRFRNLLDPLEDQKNKQLLV
YMNIVEYLKPKFVLMENVVDMLKMAKGYLARFAVGRLLQMNYQVRNGMMAAGAYGL
AQFRLRFFLWGALPSEIIPQFPLPTHDLVHRGNIVKEFQGNIVAYDEGHTVKLADKLLLKD
VISDLPAVANSEKRDEITYDKDPTTPFQKFIRLRKDEASGSQSKSKSKKHVLYDHHPLNLNI
NDYERVCQVPKRKGANFRDFPGVIVGPGNVVKLEEGKERVKLESGKTLVPDYALTYVDG
KSCKPFGRLWWDEIVPTVVTRAEPHNQVIIHPEQNRVLSIRENARLQGFPDDYKLFGPPKQ
KYIQVGNAVAVPVAKALGYALGTAFQGLAVGKDPLLTLPEGFAFMKPTLPSELA

>AtDRM1
MVMSHIFLISQIQEVEHGDSDDVNWNTDDDELAIDNFQFSPSPVHISATSPNSIQNRISDET
VASFVEMGFSTQMIARAIEETAGANMEPMMILETLFNYSASTEASSSKSKVINHFIAMGFP
EEHVIKAMQEHGDEDVGEITNALLTYAEVDKLRESEDMNININDDDDDNLYSLSSDDEED
ELNNSSNEDRILQALIKMGYLREDAAIAIERCGEDASMEEVVDFICAAQMARQFDEIYAEP
DKKELMNNNKKRRTYTETPRKPNTDQLISLPKEMIGFGVPNHPGLMMHRPVPIPDIARGP
PFFYYENVAMTPKGVWAKISSHLYDIVPEFVDSKHFCAAARKRGYIHNLPIQNRFQIQPPQ
HNTIQEAFPLTKRWWPSWDGRTKLNCLLTCIASSRLTEKIREALERYDGETPLDVQKWVM
YECKKWNLVWVGKNKLAPLDADEMEKLLGFPRDHTRGGGISTTDRYKSLGNSFQVDTV
AYHLSVLKPLFPNGINVLSLFTGIGGGEVALHRLQIKMNVVVSVEISDANRNILRSFWEQT
NQKGILREFKDVQKLDDNTIERLMDEYGGFDLVIGGSPCNNLAGGNRHHRVGLGGEHSSL
FFDYCRILEAVRRKARHMRR

>AtDRM2
MVIWNNDDDDFLEIDNFQSSPRSSPIHAMQCRVENLAGVAVTTSSLSSPTETTDLVQMGFS
DEVFATLFDMGFPVEMISRAIKETGPNVETSVIIDTISKYSSDCEAGSSKSKAIDHFLAMGF
DEEKVVKAIQEHGEDNMEAIANALLSCPEAKKLPAAVEEEDGIDWSSSDDDTNYTDMLN
SDDEKDPNSNENGSKIRSLVKMGFSELEASLAVERCGENVDIAELTDFLCAAQMAREFSEF
YTEHEEQKPRHNIKKRRFESKGEPRSSVDDEPIRLPNPMIGFGVPNEPGLITHRSLPELARG
PPFFYYENVALTPKGVWETISRHLFEIPPEFVDSKYFCVAARKRGYIHNLPINNRFQIQPPPK
YTIHDAFPLSKRWWPEWDKRTKLNCILTCTGSAQLTNRIRVALEPYNEEPEPPKHVQRYVI
DQCKKWNLVWVGKNKAAPLEPDEMESILGFPKNHTRGGGMSRTERFKSLGNSFQVDTV
AYHLSVLKPIFPHGINVLSLFTGIGGGEVALHRLQIKMKLVVSVEISKVNRNILKDFWEQTN
QTGELIEFSDIQHLTNDTIEGLMEKYGGFDLVIGGSPCNNLAGGNRVSRVGLEGDQSSLFFE
YCRILEVVRARMRGS

>AtDRM3
MADMRRRNGSGGSSNHERNEQILFPKPETLDFDLPCDTSFPQQIGDNAASSSGSNVKSLLI
EMGFCPTLVQKAIDENGQDDFELLLEILTKSTETEPPGPSFHGLMEPKPEPDIEYETDRIRIA
LLTMKFPENLVDFALDRLGKDTPIDEMVDFIVAAQLAEKYAEESEDSLDGAEINEEDEDVT
PVTARGPEVPNEQLFETMDKTLRLLEMGFSNDEISMAIEKIGTKGQISVLAESIVTGEFPAE
CHDDLEDIEKKVSAAAPAVNRTCLSKSWRFVGVGAQKEDGGGGSSSGTANIKPDPGIESFP
FPATDNVGETSRGKRPKDEDENAYPEEYTGYDDRGKRLRPEDMGDSSSFMETPWMQDE
WKDNTYEFPSVMQPRLSQSLGPKVARRPYFFYGQLGELSPSWWSKISGFLFGIHPEHVDT
RLCSALRRTEGYLHNLPTVNRFNTLPNPRLTIQDAMPHMRSWWPQWDIRKHFNSGTCSN
MKDATLLCERIGRRIAECKGKPTQQDQTLILRHCHTSNLIWIAPNILSPLEPEHLECIMGYP
MNHTNIGGGRLAERLKLFDYCFQTDTLGYHLSVLKSMFPQGLTVLSLFSGIGGAEIALDRL
GIHLKGVVSVESCGLSRNILKRWWQTSGQTGELVQIEEIKSLTAKRLETLMQRFGGFDFVI
CQNPSTPLDLSKEISNSEACEFDYTLFNEFARVTKRVRDMM

>AtMET1
MVENGAKAAKRKKRPLPEIQEVEDVPRTRRPRRAAACTSFKEKSIRVCEKSATIEVKKQQI
VEEEFLALRLTALETDVEDRPTRRLNDFVLFDSDGVPQPLEMLEIHDIFVSGAILPSDVCTD

KEKEKGVRCTSFGRVEHWSISGYEDGSPVIWISTELADYDCRKPAASYRKVYDYFYEKAR
ASVAVYKKLSKSSGGDPDIGLEELLAAVVRSMSSGSKYFSSGAAIIDFVISQGDFIYNQLAG
LDETAKKHESSYVEIPVLVALREKSSKIDKPLQRERNPSNGVRIKEVSQVAESEALTSDQLV
DGTDDDRRYAILLQDEENRKSMQQPRKNSSSGSASNMFYIKINEDEIANDYPLPSYYKTSE
EETDELILYDASYEVQSEHLPHRMLHNWALYNSDLRFISLELLPMKQCDDIDVNIFGSGVV
TDDNGSWISLNDPDSGSQSHDPDGMCIFLSQIKEWMIEFGSDDIISISIRTDVAWYRLGKPS
KLYAPWWKPVLKTARVGISILTFLRVESRVARLSFADVTKRLSGLQANDKAYISSDPLAVER
YLVVHGQIILQLFAVYPDDNVKRCPFVVGLASKLEDRHHTKWIIKKKKISLKELNLNPRAG
MAPVASKRKAMQATTTRLVNRIWGEFYSNYSPEDPLQATAAENGEDEVEEEGGNGEEEV
EEEGENGLTEDTVPEPVEVQKPHTPKKIRGSSGKREIKWDGESLGKTSAGEPLYQQALVG
GEMVAVGGAVTLEVDDPDEMPAIYFVEYMFESTDHCKMLHGRFLQRGSMTVLGNAANE
RELFLTNECMTTQLKDIKGVASFEIRSRPWGHQYRKKNITADKLDWARALERKVKDLPTE
YYCKSLYSPERGGFFSLPLSDIGRSSGFCTSCKIREDEEKRSTIKLNVSKTGFFINGIEYSVED
FVYVNPDSIGGLKEGSKTSFKSGRNIGLRAYVVCQLLEIVPKESRKADLGSFDVKVRRFYR
PEDVSAEKAYASDIQELYFSQDTVVLPPGALEGKCEVRKKSDMPLSREYPISDHIFFCDLFF
DTSKGSLKQLPANMKPKFSTIKDDTLLRKKKGKGVESEIESEIVKPVEPPKEIRLATLDIFA
GCGGLSHGLKKAGVSDAKWAIEYEEPAGQAFKQNHPESTVFVDNCNVILRAIMEKGGDQ
DDCVSTTEANELAAKLTEEQKSTLPLPGQVDFINGGPPCQGFSGMNRFNQSSWSKVQCE
MILAFLSFADYFRPRYFLLENVRTFVSFNKGQTFQLTLASLLEMGYQVRFGILEAGAYGVS
QSRKRAFIWAAAPEEVLPEWPEPMHVFGVPKLKISLSQGLHYAAVRSTALGAPFRPITVRD
TIGDLPSVENGDSRTNKEYKEVAVSWFQKEIRGNTIALTDHICKAMNELNLIRCKLIPTRPG
ADWHDLPKRKVTLSDGRVEEMIPFCLPNTAERHNGWKGLYGRLDWQGNFPTSVTDPQP
MGKVGMCFHPEQHRILTVRECARSQGFPDSYEFAGNINHKHRQIGNAVPPPLAFALGRKL
KEALHLKKSPQHQP

>AtMET2a
MEMETKAGKQKKRSVDSDDDVSKERRPKRAAACTNFKEKSLRISDKSETVEAKKEQILA
EEIVAIQLTSSLESNDDPRPNRRLTDFVLHDSEGVPQPVEMLELGDIFIEGVVLPLGDEKKE
EKGVRFQSFGRVENWNISGYEDGSPVIWISTALADYDCRKPSKKYKKLYDYFFEKACACV
EVFKSLSKNPDTSLDELLAAVSRSMSGSKIFSSGGAIQEFVISQGEFIYNQLAGLDETAKNH
ETCFVENRVLVSLRDHESNKIHKALSNVALRIDESKVVTSDHLVDGAEDEDVKYAKLIQEE
EYRKSMERSRNKRSSTTSGGSSRFYIKISEDEIADDYPLPSYYKNTKEETDELVLFEAGYEV
DTRDLPCRTLHNWTLYNSDSRMISLEVLPMRPCAEIDVTVFGSGVVAEDDGSGFCLDDSE
SSTSTQSNDHDGMNIFLSQIKEWMIEFGAEMIFVTLRTDMAWYRLGKPSKQYAPWFGTV
MKTVRVGISIFNMLMRESRVAKLSYANVIKRLCGLEENDKAYISSKLLDVERYVVVHGQII
LQLFEEYPDKDIKRCPFVTSLASKMQDIHHTKWIIKKKKKILQKGKNLNPRAGIAPVVSR
MKAMQATTTRLVNRIWGEFYSIYSPEVPSEAINAENVEEEELEEVEEEDENEEDDPEENEL
EAVEIQNSPTPKKIKGISEDMEIKWDGEILGKTSAGEPLYGRAFVGGDVVVVGSAVILEVD
DQDDTQLICFVEFMFESSNHSKMLHGKLLQRGSETVLGMAANERELFLTNECLTVQLKDI
KGTVSLEIRSRLWGHQYRKENIDVDKLDRARAEERKTNGLPTDYYCKSLYSPERGGFFSL
PRNDMGLGSGFCSSCKIRENEEERSKTKLNDSKTGFLSNGIEYHNGDFVYVLPNYITKDGL
KKGSRRTTLKCGRNVGLKAFVVCQLLDVIVLEESRKASKASFQVKLTRFYRPEDISEEKAY
ASDIQELYYSQDTYILPPEAIQGKCEVRKKSDMPLCREYPILDHIFFCEVFYDSSTGYLKQF
PANMKLKFSTIKDETLLREKKGKGVETGTSSGMLMKPDEVPKEKPLATLDIFAGCGGLSH

GLENAGVSTTKWAIEYEEPAGHAFKQNHPEATVFVDNCNVILRAIMEKCGDVDDCVSTV
EAAELAAKLDENQKSTLPLPGQVDFINGGPPCQGFSGMNRFSHGSWSKVQCEMILAFLSF
ADYFRPKYFLLENVKKFVTYNKGRTFQLTMASLLEMGYQVRFGILEAGTYGVSQPRKRVI
IWAASPEEVLPEWPEPMHVFDNPGSKISLPRGLRYDAGCNTKFGAPFRSITVRDTIGDLPPV
ENGESKINKEYGTTPASWFQKKIRGNMSVLTDHICKGLNELNLIRCKKIPKRPGADWRDLP
DENVTLSNGLVEKLRPLALSKTAKNHNEWKGLYGRLDWQGNLPISITDPQPMGKVGMCF
HPEQDRIITVRECARSQGFPDSYEFSGTTKHKHRQIGNAVPPPLAFALGRKLKEALYLKSSL
QHQS

>AtMET2b
METKVGKQKKRSVDSNDDVSKERRPKRAAACRNFKEKPLRISDKSETVEAKKEQNVVEE
IVAIQLTSSLESNDDPRPNRRLTDFVLHNSDGVPQPVEMLELGDIFLEGVVLPLGDDKNEE
KGVRFQSFGRVENWNISGYEDGSPGIWISTALADYDCRKPASKYKKIYDYFFEKACACVE
VFKSLSKNPDTSLDELLAAVARSMSGSKIFSSGGAIQEFVISQGEFIYNQLAGLDETAKNHE
TCFVENSVLVSLRDHESSKIHKALSNVALRIDESQLVKSDHLVDGAEAEDVRYAKLIQEEE
YRISMERSRNKRSSTTSASNKFYIKINEHEIANDYPLPSYYKNTKEETDELLLFEPGYEVDT
RDLPCRTLHNWALYNSDSRMISLEVLPMRPCAEIDVTVFGSGVVAEDDGSGFCLDDSESST
STQSNVHDGMNIFLSQIKEWMIEFGAEMIFVTLRTDMAWYRLGKPSKQYAPWFETVMKT
VRVAISIFNMLMRESRVAKLSYANVIKRLCGLEENDKAYISSKLLDVERYVVVHGQIILQLF
EEYPDKDIKRCPFVTGLASKMQDIHHTKWIIKRKKKILQKGKNLNPRAGLAHVVTRMKP
MQATTTRLVNRIWGEFYSIYSPEVPSEAIHEVEEEEIEEDEEEDENEEDDIEEEAVEVQKSHT
PKKSRGNSEDMEIKWNGEILGETSDGEPLYGRALVGGETVAVGSAVILEVDDPDETPAIYF
VEFMFESSDQCKMLHGKLLQRGSETVIGTAANERELFLTNECLTVHLKDIKGTVSLDIRSR
PWGHQYRKENLVVDKLDRARAEERKANGLPTEYYCKSLYSPERGGFFSLPRNDIGLGSGF
CSSCKIKEEEEEERSKTKLNISKTGVFSNGIEYYNGDFVYVLPNYITKDGLKKGTSRRTTLKC
GRNVGLKAFVVCQLLDVIVLEESRKASNASFQVKLTRFYRPEDISEEKAYASDIQELYYSH
DTYILPPEALQGKCEVRKKNDMPLCREYPILDHIFFCEVFYDSSTGYLKQFPANMKLKFST
IKDETLLREKKGKGVETGTSSGILMKPDEVPKEMRLATLDIFAGCGGLSHGLEKAGVSNT
KWAIEYEEPAGHAFKQNHPEATVFVDNCNVILRAIMEKCGDVDDCVSTVEAAELVAKLDE
NQKSTLPLPGQADFISGGPPCQGFSGMNRFSDGSWSKVQCEMILAFLSFADYFRPKYFLLE
NVKKFVTYNKGRTFQLTMASLLEIGYQVRFGILEAGTYGVSQPRKRVIIWAASPEEVLPEW
PEPMHVFDNPGSKISLPRGLHYDTVRNTKFGAPFRSITVRDTIGDLPLVENGESKINKEYRT
TPVSWFQKKIRGNMSVLTDHICKGLNELNLIRCKKIPKRPGADWRDLPDENVTLSNGLVE
KLRPLALSKTAKNHNEWKGLYGRLDWQGNLPISITDPQPMGKVGMCFHPEQDRIITVREC
ARSQGFPDSYEFSGTTKHKHRQIGNAVPPPLAFALGRKLKEALYLKSSLQHQS

>AtMET3
MKTKAGKQKKRSVDSDDDVSRERRPKRATSGTNFKEKSLRFSEKYETVEAKKEQIVGDD
EKEEKGVRFQSFGRVENWTISGYEDGSPVIWISTVIADYDCRKPSKKYKKLYDYFFEKAC
ACVEVCKNLSTNPDTSLKELLAAVVRSMNGRKIFSSGGVIQEFVISQGEFIYNQLAGLDET
SKNHETKFVDNRVLVSLRDESRKIHKAFSNVALRIDESKVLTSDQLMDGGEDEDLKYAKL
LQEEEHMKSMDRSRNKRSSTTSAPNKFYIKINEDEIAHDYPLPSYYKNTKDETDELVLFNA
GYAVDARNLPCRTLHNWALYNSDLMLISLEFLPMKPCADIDVTYLGQIKEWKIDFGEDMIF
VLLRTDMAWYRLGKPSEQYAPWFEPILKTVRIGTSILALLKNETRMAKLSYTDVIKRLCGL

EENDQAYISSTFFDVERYVIVHGQIILQFLTECPDEYIKRCPFVTGLASKMQDRHHTKWIIK
KKRKMLQKGENLNLRRGKAPKVSKMKAMQATTTRLINRIWGEFYSIYSPEDPLEEIGAEE
EFEEVEDVEEEDENEEEDTIQKAIEVQKADTLKKIRGSCKEMEIRWEGEILGETCAGEPLY
GQALVGGRKMDVGGAVILEVDDQGETPLIYFVEYMFESSDNSKKLHGKLLQRGSETVLG
TAANERELFLTNECLTVQLKDIKGTVSFEIRSRPWGHQYKKEHMAADKLDRARAEERKA
KDLPIEYYCKSLYSPEKGGFFSLPRSDMGLGSGFCSSCKIRENEEERSKTKLNDSKTRFLSN
GIKYSVGDFVYQIPNYLSKDRGKRRPVFKYGRNVGLRAFVVCQILDIVDLKEPKKGNTTS
FEVKVRRFYRPDDVSAEEAYASDIQEVYYSEDTYILPPEAIKGKCEVMKKTDMPLCREYPI
LDHVYFCDRFYDSSNGCLKKLPYNMMLKFSTIKDDTLLREKKTETGSAMLLKPDEVPKG
KRLATLDIFAGCGGLSYGLEKAGVSDTKWAIEYEEPAAQAFKQNHPKTTVFVDNCNVILRI
SWLRLLINDRAIMEKCGDVDDCISTTEAAELATKLDENQKSTLPLPGQVDFISGGPPCQGF
SRLNRFSDGSWSKNQCQMILAFLSFADYFRPKYFLLENVKTFVSFNEGHTFHLTVASLLEM
GYQVRFGLLEAGAYGISQPRKRAFIWAAAPNEVLPEWPEPMHVFNNPGFKIPLSQGLHYA
AVQSTKFGAPFRSITVRDAIGDLPPIESGESKINKEEMRGSMTVLTDHICKKMNELNLIRCK
KIPKTPGADWRDLPDEHVNLSNGIVKNIVPNLLNKAKDHNGYKGLYGRLDWHGNLPTCI
TNLQPMGLVGMCFHPDQDRIISVRECARSQGFPDSYKFSGNIKDKHRQVGNAVPPPLAFA
LGRKLKEALHLRNI

>CcCMT3_like2
MARGKRKSSAANDDASASSSILEKKPKLVEDKNEEISSNVGVVEASAVLDRVSPAKRSGQ
KLDKPKKEDEEDEDEVESRFIGDPVPDDEARQRWPHRYATINNNDINFAKKKAGKSGSLE
TLKGMAPQKELMQARRHFSEALVDGRVNFKLGDDGYVQAGEGEDSYICRIVEMFEGMD
CALYFSAQWFYRAKDTIIQACSDLIDDKRVFLSEIKDDNPLDCLLEKLKIVRVPLDADIADK
QARLADGDYYYDMSYLVPYSTYQNLPPVYGKHLSYILVSERLKFLLYEQPITDNDGDGNE
SDSTISSESDVNGVVSEVPQVQDGKRSEMRMLDLYSGCGAMSTGLCLGANMGDVNLVTR
WAIDLNRYACESLKLNHPETEVRNESAEDFLMLLKEWEKLCQSFSLVGGGDSQQRMNPAS
IEEDEGEADDDDDDDSDGLDEEVFEVEKVLSICYGDPKEIKKPGLYLKIRWKGYGPEEDT
WEPMEGLCDCHDKIKAFVVKGFNSKILPLPGDVDVICGGPPCQGISGFNRFRNKDKPLED
EKNKQLVVYMDIVEYLKPRFALMENVVDIVKFAKGFLGRYALGRLVSMNYQARVGLMV
AGSYGLPQFRRRMFMWGARPSEFNLFNTSFDVQKLPQYPLPTHNVVTRGVSPLEFESNTV
VHEEGQKVELEKELFLGDAISDLPPVPNDETRDEMPYEEMPKTEFQKFIRLKKDDMPGFS
ASGQDSSDHLLYDHRPLKLNDDDYQRVCQIPKRKGANFRDLKGVRVRKDNHVEWDPDV
ERVYLPSGKPLVPDYAMTFVDGRSSKPFGRMWWDETVPTVVTRAEPHNQAILHPLQDRV
LTIRENARLQGFPDYYKLVGPIKERYIQVGNAVAVPVARALGYSLAMSCKGSSGAEATFTL
PSKFPNIQPVTSPSVDQQNQ

>CcCMT3_like3
MPSSKRKSRASLKDADLASPTSEKRPKSEEEEKGEILPNADAVEAPAVNNGDSSVRSSGRR
SENNKEEAELNDDEESRLVGDPIPDDEARQRWPHRYLGKNDAICYLYGTSLTEPQKELIQA
RRHFTEALVDGCIPFKLGDDGYVQAGEGEESYICRIVELFEGVDGAPYFYAQWFYRAKDT
ACSNLIDDKRVFLSEIKDDNPLDCLLQKLKIVRVPLDVDVASKRAMLLDGNYYYDMLYLV
PYSTYQNLPLVGSHSSNVLKLKTFIGTQQLYLYTDNEADGNESESTISSESDSNAAVTETSK
IQEDDKSEMRMLDLYSGCGAMSTGLCLGANMADVNLVTRWAVDLNRYACESLKLNHPE
TEVRNESAEDFLWLLKEWHKLCQSFSLVGGGDSQLSGDPMIVKEDETEDAVDDDDDNDG

LDEEVFEVDKILSICYGDPKGLKKPGLYLKIRWKNYGPAEDTWEPIDGLGDCQEKIKQFVV
NGFKSKILPLPGDVDVICGGPPCQGISGFNRYRNKDKPLEDEKNKQLVVYMDIVEYLKPRF
ALMENVVDIVKFAKGFLGRYALGRLVSMNYQVRIGLMTAGSYGLPQFRMRMFMWGARP
SEKLPQYPLPTHNVVARGVSPVEFESNAVVYDEVSGIELEKELFLGDAISDLPPVANDEER
DEIPYQEMPKTEFQKFIRLKKEDMPGFSMVGVESSDHPLYDHRPYKLNDDDYLRVCQIPK
RKGANFRDLKGVQVRDDNHVEWDPDVERVYLPSGKPLVPDYAMTFVDGRSSKPFGRLW
WDEIVPTVVTRAEPHNQAILHPLQDRVLTIRENARLQGFPDYYKLVGPIKERYTQVGNAVA
VPVARALGYSLAMSCKGTASEGPVFRLPERFPNIEAVPVAVAVEENQ

>CcCMT2_like1
MVNSAGTGEGSGSCPPSKPPMAPSSSQPSSITANYDSPALHFGQISRRRSPRLLNCSKDGKD
SSVDFNKCPKQKKLRTTSPSDSFPSSPNDAPFLIGDPVSDEEARRRWPWRYECKVLSVME
VLIFSRVPDRGDDNEWALLFFLEHEGYLRSDLFRPSVTQTKKEPFKDDDDKLIANVKCHY
LQAKVETHVFDLGDCACVKGEEDGHNHVGRILEFFKTNDSKDFFRIQWFFRAEDTDEAA
SHHKKRLFYSTLRNDNALDCIVSKVDFRSIPACDYYYDMKYNIDYSTYCTIKDDDCCLFSS
HNKKEMHSNGSKTNLNGTLTSFKPHKLELSLLDMYSGCGGMSTGLCFGAKLSGVDLSTI
RNESAEDFLDLIKEWDKLCKKYMVKEEKTQGNDSTFAGSADDKPSKAKKIVPEDEYEVE
RLVDICYSDLDGTSKRGLKFKVRWAGYGPSDDTWEPIKELSNCQEKIREFVQKGIKTNKLP
RPGDVDIICGGPPCQGISGFNRHRNFESPLEDEKNYQIVVFMDIINFLRPKYILMENVVDIL
RFANGRLARYAISCLVRDYYQVRLGIMAAGCYGLPQFRLRQLPPFPLPTHDVVFKYGGAS
GFERNVVAYDEGQFRNLEKPVLLKDAISDLPLVSNSEVRDKMMYRSAPETEFQKYIRATK
SDMLGIASGSSSEVEKSVLHDHRPLQLNEDDYLRGANFRELPGIVVDDDNVVSRAPEAEL
MPSGKHWVPDYAINLHERKSTKPFARLWWDETVSTVICTPNFRCEAVLHPEQDRVLTIREN
ARLQGFPDFYALCGTVNERYRQVGNAVAVPVGRALGYTLGMAVQKLCGDEPLITLPPEFA
HSTTLDLLQASSLATEP

>CcDRM2_like2
MGDHAPGDDSENIDWDTEDELEIQNIAPSSCAQLITRDTEAVISNGKASSSAGPSNTKLVE
HFLGMGFREQWVTKAIEKNGEGDHESILDTLFAYQRFKCGDYELPAKRVVAPGLVGSHSG
LDIQGVQIFGLLMRISVLQALEDPQQGHNSCHPNHLNSPQQQQCVNDDDLSSDYDESLLD
DFSESDSWLGSETEDFDSLPEHEKTLKYLVAMGYTEEEASIAMERCELAYISTNPMGPGEQ
RNLLWPCGTRTGPGASIAVLTDFIGAAQNAKTEPVFFEDEKRKNLGENGKLKKRKLYELE
AWKRNKQKGPLTEQDEVIRLPNPMTGFGVPSEPSSMVTHRTLPDDAIGPPFFYYENVALAP
KGSWDTISRFLYDVEPEFVDSKYFCAAARKRGYVHNLPINNRFCILPLPPRTISDALPLTKR
WWPEWDKRTKLNCLQTVIASAKLTERIRKDLEKYSDYVPLDVQKKVIEDCRKWNLVWV
GKNKVAPLEPDEVEMLLGFPRNHTRGGGISRTDRYKSLGNSFQGNPCQIGFGDWLRVFMP
SLPKRLDSTSNPGYRWSTHSLGYRSANNPLVDTVAYHLSVLKDIFPKGINMLSLFSGIGGA
EVALHRLGILLNNIVSVEISEANRDIVRSWWEQTNQKGNLVHLPDVQQLNGDQLEQFISSF
GGFDLVVGGSPCNNLTGSNRVSRDGLQALDDCLVVGYWLNDIESGYLSNAACIAPSKCFA
FGTCVGIVLRPNVKVRLITGLDWTGWARVKKTGEEL

>CcCMT2_like2
MFTGKKSEPCDEVSRTNSASKMVVRGSTRMNSDINVAKWGKFSTTGSDALALTLHSLSSD
FYYNSDESPVPLKVYDPSLAHGKSVRRSPRFTMGESIITTKGASKNKSVLGERVEGSSSGR

SSRLSSSTALIVGTLDRQRSPKSKALPRRRLRRSPRLSPVTSHAENAGSNKLKRLPQKCLRR
SPRLSPIPSCPQIEWEGLHTRVKGERTTDGPKLKRIKSCVEELNSNVVTEKCTKKSSARVQH
GNGNCEKLAVPALSSAETVKDDFIPNGDTARPCGLEFHVRKKEDSEVKSTSIRTHDKKAAS
VGNIDFGYNKAPPVRCKTSVSSTKQQSSTKKQRKLKRASFFVGEPVPEDEAQERWHWRY
ELKFFAVSKIRVTSEMGFEEEVNGVFQRKLGAETDLNKGKTGQRRKGQSWILNAGEEDEL
HLNVMCHYLQANVDGCIYRLGDCARIKLHGENNNYKDKDEHRTYTSESASKNRARAQL
LIHGEGKREHVGRIVEFFKTSDDENYFRVQWFFRAEDTVMKQAAAFHEKKRLFSSTLMN
DNLLDCILSKVKITEKAPALGLQSAIQPSEYYCDMEYSVKYSTFRSLATATNSLVARCDLTL
PSSLDANNVTITTTPLELSSCEPYKAELALLDLYAGCGGMSTGLCLGAKISGVKLVTRWAI
DYHKSACDSFKQNHPETQVRHITAEDFLELLKEWEKLCKLYVLNDTDRGLETSSNRTKKS
IIRESSLLDAEVAPGEYEVSSLVDICYGDPSSTGKHGLKFKVRWKGYSPSEDTWELIQDLSD
CQGHIKDFVRSGYMSKILPRPGEVDVICGGPPCQGISGYNRFRNTDDPLTDERNQQIIVFFD
IVNFLKPKYALMENVADILRYDKASLGRYAISRLVHMNYQSRLGIMAAGSYGLPQFRLRVF
LWGALSSETLPQFPLPTHEVIVRYFPSAEFEQNTVAYDEGQPRELKEATVLRDAISDLPAVT
SHEDREEMAYDMPPETEFQKYIRLTKDEINGSTLKGVTDWRSSVLTDHRPYKLSEDDFHR
VCHVPRRKGANFRDFPGLVVGADNLVRRDPTKEPVLLPSGRPLVPDYVFTFEKGKSKRPF
SRIWWDENVPTVVTFPNLHSQRAIHPEQDRVLTIREYARLQGFPDHYRFCGTVKERYCQV
GNAVAVFVSKTLGYALGMAFQKLSGDEALMTLPPDFAFQVPPLDQFSAQL

>CcCMT3_like1
MAKRVKRDLKQSEEAIDSSVPSSDSKSSPPSKKSKTSPAAAAADDDARFIGKPVPADQARA
KWPHRYESKNKVKVIASSNGELDGKEIIQAKCHYTKAVVDGIAFDLNDDAFVKAEEGKP
DFIARIVEMFETVDRELYCSAQWFFRAEDTVIKSQAHLIDKRRVFYSDMKDDNPLDSIVSK
VKIVQLSPNVDLAEKEKALSSFDLYYDMKYSMPVTFTTLHTEKSITESDESSVISGDASSD
GVVEKSNKKAKSTEVKECVESQMTLLDLYSGCGAMSTGLCHGTNMSGVKLVTKWAVDI
NKHACESLKLNHTETEVRNEAAEDFLSLLKEWKNLCKEFCLLGSQHAEDTSIKSEESDSQ
EKEGNPDPSDGEFEVGKLLAVCYGDPNKVNNKKLHFKVFYFPIVVEVRWKGYGPSYDTW
EPFDGLSNCTDAMKEFVSRGYQSRILPLPGDVDFICGGPPCQGISGHNRFRNYTDPLKDPK
NHQLVVYMDIIEFLKPKFVLMENVCDIVKFADGILGYHAVGRLVSMNYQTRMGIMAAGS
YGDCIVGSDNDKSYKLEKSILLGDAISDLPEVTNNNGKDEMEYAGAPRTSFQKYIRMRKQ
AVAKDASKRKMLYDHRPLELNEDDYARVCQIPKIKGANFRNLPGVMVGKNNKVEWDPS
VERVMLPSGKPLVPNYAMTFVRGTSRKPFGRLSMDDIVTTVVGRAEPHNQVLLHPNQDR
VLTIRENARLQGFPDHYKLSGPVKERYLQIGNAVSFSVSTALGYTLAKAVQGVCTSKPLTL
PIKFPDCLGQSTVKQAPQESE

>CcMET1_like
MKKKGKQVKATTEMSDVTVDLKTKAATKPKQKRGRSGSSEDVPVSRKMPKRAASCTNF
KSKPLRLSEKSATIENKKVQVVEEEIAAISLIAGPDDPRPNRRLTDFVFHDADGMPQPVEM
LEVDDIFISGIILPLEKASEKEKETGVRCDGFGRIEDWSISGYEDGSPVIWISTELADYDCVK
PAASYKKLFGLFYEKAHACVEVYKRLSKSAGGNPELSLDELLAALVRSMSGSKNFPHGAS
IRDLIISWGGFIFEQLVGLDEAPNGTDQPFVEIPVLAALRDESKKDEECAGMNVPPGGLMN
APLKISDGEKASKSNGPKPAVDEDEDMKLARVLQENENWQALKQKKRQRTSTSSSKLYIQ
INEDEIANDYPLPAYYKTTAQETDEYIIFDDFDTVDPDQLPRSMLHNWSLYNCDSRLISLEL
LPMKPCADIDVTVFGSGIMTADDGSGFCLDNEAGSASGSSGVQNEDGIPIYLSAIKEWMIE

FGSSMVFISIRTDMAWYRLGKPSKQYAPWYQTVLKTARLAIAIITLLKEQTRASKLSFSEVI
KRLYVVVHGQIILQQFAEFPDDTIRRSAFVSGLEDKMEERHHTKWLVKKKAILTKAENMN
PRAAMGPVISKRKAMPATTTQLINRIWGEFYSNYSPEEVKEGDSLDAKKDEEEEEQEENE
QEDCEEPEEENPILPQEPEKPSPASKQKKTRCSKTDINWVGQAVRKMCDGKALYKEAVIRG
EVVALGSSVLVETSGSEEDSIYYVEYLFEDSDSRKFVHGRLMLRGRQTVLGDIANEREVFL
TNDCMEFELDDVIQTVVVQIRTLPWGFEHRKANANFDKMDRAKAEDRKNKGLPIEFYCK
SLYWPQRGAFFCLQTDKMGLGNGVCHSCEFMEAEKKKEAFVLDESKTGFTYMATEYHV
DDFLYVGPHHFDTDERGNETYKGGRNVGLKAYVVCQLQQIEAPKTSKRADPDSVMVQV
RRFYRPEDLSSDKAYRSDIQEVYYSEQVHKLPLSAIEGKCEVRRKKDLSSLDSTYIFEHVFF
CERLYDPAKGSLKQLPVHIKLTPPKESLVSDAAIRKRKGKSKEGENDVDMIDNQESSASKN
CLATLDIFAGCGGLSEGLQKAGASVTKWAIEYEEPAGDAFKLNHPDALAFVHNCNVILRAI
MTACGDTDDCISTSEADELAAKLEEDVINNLPRPGQVDFINGGPPCQGFSGMNRFNQSTW
SKVQCEMILAFLSFAEYFRPKFFLLENVRNFVSFNKGQTFRLALASLLEMGYQVRFGILEA
GAFGVSQSRKRAFIWAASPEEVLPEWPEPMHVFAGPELKVTLNRNTQYAAARSTATGAPF
RAITVRDTIGDLPAVGNGASAATIEYKNESVSWFQKRIRGDASVLTDHISKEMNELNVIRC
QRIPKRPGADWRDLPEEKVKLSTGQMVDLIPWCLPNTAKRHNQWKGLFGRLDWEGNFP
TSITDPQPMGKVGMCFHPDQDRILTVRECARSQGFPDRYKFSGNIQHKHKQIGNAVPPPLA
YALGRKLKEAVEAKQRQLDHHSCL

>CcDRM2_like1
MDGDSSHEGSDNIDWNTDDELEIANISPSSPVSTSTNGAVISEFGEVVHNCTQIIADYPTPG
MIVIYEPLISDGSKRISWSLCYKAFGFGISIGEVAPRDVIGWTHDLRRYFRGYFVISCLTLVV
IAKAPFKPRSILQIPPTMSSSSKCPSKLQFMTMGFPEAMVTKVIAELGEDNTDAIVDTLLTY
SIDSLGENEMDVVVDETPNQERELNDPYLKSESHPESKFSDLDSIWSDEDSDESFEKDDPL
VCLIDMGYSPEEASAAISRCGKNAPLSELVDFISAANVSKEYDAEMNANVLGSCSHLLPK
DKKRKFQKDNFWSKNKKFDRKNESKSKDEDHEVLHLPNPMVGFGVPREPFHVVRRTLPE
AAIGPPFFYYENVALTPKGAWNRIKSFLYEIDPEFVDSKFFCAAARKRGYIHNLPLENRFPI
QPLPPLTIFEALPGTKKWWPSWDKREQLNCILTCIGSAQLTDRIRLALENSNTEPSLHVKNY
IIGQCRKWNMVWTGKTRVAPLEPDEIELIMGYPIYHTRGASRVERYKGLGNAFQVGTLKP
PTFYFFNRYYHLKAGLHIFQAKCLIGIQHLVIYILHLIFYTFGGKTSRVFQQEFHILEVDTVA
YHLSVLKNLYPNGMNILSLFSGIGGAEIALHKLGIPLNNVVSVEKSMVCRNILQGWWEQT
NQKGNLVHLSDVQDVTLNKLNQWIDSFGGFDLVIGGSPCNNLAGGNRRTRDGLEGGHSS
LFFDYFRILDGVKNLMKNRS

>CcDRM2_like3
MDGNASGEDYENIDWDTEDELEIQNIVPSTCSNLVTRNASIVGNGEASSSAGPSNPNLLQH
FLGMGFSEQLIVKAIKENGEANTESILESLLTYAALEDSPDELNPCHLNSPQQQQCVDNGQ
LSSDYDESFLDDISESDSWSGSEVAKPKISIGANGGLKKRKLYELELWKRKKRKGLINEED
DVLRLPNPMIGFGVPTDTMVVTHRTLPEAAIGPPFFYYENVALAPKGVWDTISRFLYDVQP
EFVDSKYFCATARKRGYVHNLPIHNRFPLLPLPPRTINDALPLTKRWWPEWDKRTKLNCL
QTVIGSAKLTDRIRKALEKWGDDPPLHVQKYVIEECRKWNLVWVGKNKLAPLEPDEFEM
LLGFPRNHTRGGGISRTDRYKSLGNSFQDMFPNGLNLLSLFSGIGGAEVALHRLGIPLKNV
VSVEISEANRDIVRSWWEQTNQKGNLIHLADVQQLNGDRLEQFMGSFGGFDLIVGGSPC
NNLAGSNRVSRDGLEGEQSSLFYDYFRILDLVKCIMNKQQ

>CcDRM3_like1
MDKTLRLLEIGFSEQEISAAIEKYGSEVSISELADSIVCDRMGGPCIKTEEDPFGANSWMTG
NKFKSSSMGAERVLDASFYSNLALRTEESSQAAASQIRDFDIGDSCKGKQPKEETADELITI
QRPKPEFDDLNSYSGPACTVPKPPVSSKVLQRQLKYKARRMAATGVPKLIQPVSCSSVDQ
MVAKAPLFFYGNVMNLSQDSWVKISQFLYAIEPEFVNTQFFSALSRKEGYIHNLPTKNRFH
ILPKPPMTIEEVIPQTKKYWPSWDTRKQLTCINSETIGISQLCDRLRNILIDSKGLLSVEQQK
DLLHQCRSLNLMWVGRNRVSPIEPELVERILGYPMYHTREDGLSLGERLQSLKHSFQTDT
LGYHLSVLKSMYPEGLTLLSIYSGVGGAEITLNRLGIRLKAVVSVEPSEIKRKILRQWWDK
SDQTGELVQIENIQKLSSSKLESLIKKFGVFDFIICQNPYTYAPKSVTMAAAETESFAGLDFS
LFYEFVRVLQRVRSAIKTR

>GmCMT1
MPSKRKTRSSASPAAAPPSKRASRSSASRVADSAPVKSEAEEVVAASSVVKEEAQASFTDV
TDGNVSDGEGTNARFVGEPVPDEEARRRWPKRYQEKEKKQSAGPKSNRNDEDEEIQQAR
RHYTQAEVDGCMLYKLYDDAHVKAEEGEDNYICKIVEIFEAIDGALYFTAQWYYRAKDT
VIKKLAYLIEPKRVFFSEVQDDNPLDCLVEKLNIARITLNVDLEAKKETIPPCDYYCDTQYL
LPYSTFVNLPSENGESGSETSSTISSETNGIGKYEVNSQPKEAFLPEESKDPEMKLLDLYCG
CGAMSTGLCLGGNLSGVNLVTRWAVDLNQHACECLKLNHPETEVRNESAENFLSLLKEW
QELCSYFSLVEKKVSHEKYVNLFSEDDDDTSSNEEVNSEDDNELNEDDEIFEVSEILAVCY
GDPNKKKEQGLYFKVHWKGYESALDSWEPIEGLSNCKEKIKEFVSRGFKSQILPLPGDVD
VICGGPPCQGISGFNRFRNKESPLDDEKNKQLVVFMDIVQYLKPKFTLMENVVDLVKFAE
GFLGRYALGRLLQMNYQARLGIMAAGAYGLPQFRLRVFLWGAAPSQKLPQFPLPTHDVIV
RGVIPLEFEINTVAYNEGQKVQLQKKLLLEDAISDLPRVQNNERRDEIKYDKAAQTEFQRF
IRLSKHEMLELQSRTKSSKSLLYDHRPLELNADDYQRVCRIPKKKGGCFRDLPGVRVGAD
NKVEWDPDVERVYLDSGKPLVPDYAMTFVNGTSSKPFARLWWDETVPTVVTRAEPHNQ
AILHPEQDRVLTIRENARLQGFPDFYKLCGPVKERYIQVGNAVAVPVARALGYTLGLAFEG
STSTSDDPLYKLPDKFPMIRDRVSSVSSEDDV

>GmCMT4
MNDNLIDCIMGKANVTHITPRVGLKLASISSSDFYYDMEYCVDYSTFRNIPTDASTVTESQ
PCSELNKTELALLDLYSGCGGMSTGLCLGAKTASVNLVTRWAVDSDRSAGESLKLNHSDT
HVRNESAEDFLELLKAWEKLCKRYNVSSTERKLPFRSNSSGAKKRGNSEVHEISDGELEV
SKLVDICFGDPNETGKRGLYLKVHWKGYSASEDTWEPIKSLSKCKESMQDFVRKGMKSN
ILPLPGEVDVICGGPPCQGISGYNRFRNCASPLDDERNRQIVIFMDMVKFLKPRYVLMENV
VDILRFDKGSLGRYALSRLVHMNYQARLGIIAAGCYGLPQFRLRVFLWGAHPSEVIPQFPL
PTHDVIVRYWPPPEFERNVVAYDEEQPRELEKATVIQDAISDLPAVMNTETRDEMPYQNPP
ETEFQRYIRSTKYEMTGSKSNGTTEKRPLLYDHRPYFLFEDDYLRVCQIPKRKGANFRDLP
GVIVGADNVVRRHPTENPLLPSGKPLVPEYCFTFEHGKSKRPFARLWWDENLPTALTFPSC
HNQVVLHPEQDRVLTIREFARLQGFPDYYRFYGTVKERYCQIGNAVAVPVSRALGYALGL
ACRKLNGNEPLVTLPSKFSHSNYLQLSKCVFGNTSNEVNSRQFRALDAEVTPGSIGQDSRV
EDSTQLQTCYNNQPGNTD

>GmMET1

MGSASLLNPSQPGVKKNDKSKQKSVVSKTEEEVMFKDKQKKRSLSESSEQTAAMRKMP
KRAAACKNLKEKSFLIYEKSCLIETEKDHIVEEESLAVRMTAGQDNGCPNRRITEFILHDET
GKSQPLEVLEVDDLFITGLVLPLEASSGKKKEKGVKCEGFGRIESWDISGYEDGSPVIWLS
TEVADYDCQKPAASYKKVYDLFLEKARACVEVYKKLAKSSGGDPDISLDELLAGMVRS
MSGSKCFSGAASIKDFVISQGEFIYKQLVGLDMTSKANDRMFADIPALIALRDESKKQVHA
QVMPSNGSLRIDSGVGDEENKNQMDSVASVNEEDEDAKLARLLQEEEYWQSMNQKKNS
RSASASNKYYIKINEDEIANDYPLPVYYKTSLQETDEFIVFDNDYDIYDTQDLPRSMLHNW
SLYNSDARLVSLELLPMKPCSDIDVAIFGSGIMTSDDGSGFHLDTEAGKSSSVGSGAQVAD
GMPIYLSAIKEWMIEFGSSMIFISIRTDLAWYRLGKPAKQYAPWYDTVLKTARLAISIITLLK
EQSRVSRLSFGDVIRKVSEFDKKDGSYISSDPLTVERYVVVHGQIILQLFAEFPDDKIRKSAF
VTGLTNKMEERHHTKWLVKKKKVVPRSEPNLNPRAAVGPVVSKRKAMQATTTRLINRIW
GEYYSNHLPEDAKEGIASELKDEDEVEEQEENEDDDNEETILLEGTPKAHSASKQTKKFS
AETEIRWEGEPEGKTSSGYPVYKQAIIRGEVISVGRSVLVEVDETDEFPDIYYVEYMFESKI
GRKMFHGRMMQRGCQTVLGNAANEREVFLTNECRDLGLHDVNQTVVVNIQNRPWGHQ
HRKDNIIADRVDRAQAEERKKKGLPTEYYCKSLYWPERGAFFSLPLDTLGLGSGVCPSCKI
QDAEKEKDVFKVNSSKSGFLLKGTEYSLNDYIYVSPFEFEEMIEQGTHKSGRNVGLKAYV
VCQVLEIVVKKEIKEAEIKSTQVKIRRFFRPEDVSNEKAYCSDIQEVYYSDETHIISVESIEG
KCQVRKKNDIPECSALGRMFQNVFFCELLYDPATGSLKKLPAHVKVKYSSGQTSDAAAR
KRKGKCIEGDDVLESPNEGKTLNEKRLATLDIFAGCGGLSEGLQQSGVSSTKWAIEYEEPA
GDAFKANHPEALVFINNCNVILRAVMEKCGDTDDCISTSEAAELAAKLDEKEISSLPMPGQ
VDFINGGPPCQGFSGMNRFNQSSWSKVQCEMILAFLSFADYFRPRYFLLENVRNFVSFNK
GQTFRLTLASLLEMGYQVRFGILEAGAYGVSQSRKRAFIWAASPEDVLPEWPEPMHVFSA
PELKITLSENVQYAAVRSTANGAPLRSITVQDTIGDLPAVGNGASKGNMEYQNDPVSWFQ
KKIRGDMVVLTDHISKEMNELNLIRCQKIPKRPGADWRDLPEEKIKLSTGQVVDLIPWCLP
NTAKRHNQWKGLFGRLDWQGNFPTSITDPQPMGKVGMCFHPDQDRILTVRECARSQGFP
DSYQFAGNIIHKHRQIGNAVPPPLASALGRKLKEAVDSKSST

>GmMET2
MGSASLLNPSQPGVKKNSKSKQKSVVSKTEEKVMVKDKQKKRSLLESSEQPAATRKMPK
RAAACKNLKEKSFLISEKSCLIEMEKDQIVEEESLAVRMTAGQDDGRPNRRITEFILHDATG
KAQPLEVLEVNELFITGLILPLEVSTGKKKEKGVKCEGFGRVESWDISGYEDGSPVIWLST
DIADYDCQKPAASYKKVYDLFLEKARACIEVYLKLAKSSGGDPDISLDELLAGMVRSMS
GSKCFSGTASIKDFVISHGEFIYKQLIGLDMTSKANDRTFADIPALIALRDESKKQANYVHA
QVMPSNGSLRIDSGVGDEENKNQMDSVASVNEEDEDAKLARLLQEEEYWQSMKQKKNS
RPTSVSNKYYIKINEDEIANDYPLPAYYKTSLQETDEFIVFDNDYDIYDTQDLPRSMLHNW
SLYNSDARLVSLELLPMKPCSDIDVAIFGSGIMTSDDGSGFHLDTEAGQSSSVGSGAQVAD
GMPIYLSAIKEWMIEFGSAMIFISIRTDLAWYRLGKPAKQYAPWYDTVLKTARLAISIITLL
KEQSRVSRLSFGDVIRKVSEFNQKDGSYISSDPLTVERYVVVHGQIILQLFAEFPDDNIRKSS
FVTGLTNKMEERHHTKWLVKKKKVVPRSEPNLNPRAAVGPVVSKRKAMQATTTRLINRI
WGEYYSNHLPEDSKEGIASELKDEDEVEEQEENEDDDNEEETILLEGTPKAHSASKQTKKI
SADTEIRWEGEPEGKTSSGYPVYKQAIIRGEVISVGRSVLVEVDETDEFPDIYYVEYMFESK
IGRKMFHGRMMQCGCQTVLGNAANEREVFLTNECRDLGLHDVKQTVVVNIQNRPWGH
QHRKDNIIADRVDRTQAEERKKKGLPTDYYCKSLYWPERGAFFTLPHDTLGLGSGVCPSC
KIQDAEKEKDVFKVNSSKSGFLFNGTEYSLDDYVYVSPFEFEEKIEQGTHKSGRNVGLKA

YVVCQVLEIVVKKEIKQAEIKSTQVKIRRFFRPEDLSNEKAYCSDIREVYYSDETYIISVESI
EGKCQVRKKNDIPECSALGGIFQNVFFCELLYDPATGSLKKLPAHIKVKYSSGQTSDAAAR
KRKGKCIEGDGVSESTKEGKTLNDKRLATLDIFAGCGGLSEGLQQSGVSSTKWAIEYEEPA
GDAFKANHPEALVFINNCNVILRAVMEKCGDTDDCISTSEAAELAAKLDEKEISSLPMPGQ
VDFINGGPPCQGFSGMNRFNQSSWSKVQCEMILAFLSFADYFRPRYFLLENVRNFVSFNK
GQTFRLTLASLLEMGYQVRFGILEAGAFGVSQSRKRAFIWAASPEDVLPEWPEPVHVFSAP
ELKITLSENVQYAAVRSTANGAPLRAITVRDTIGDLPAVGNGASKGNMEYQNDPVSWFQK
KIRGDMVVLTDHISKEMNELNLIRCQKIPKRPGADWRDLPEEKIKLSSGQVVDLIPWCLPN
TAKRHNQWKGLFGRLDWQGNFPTSVTDPQPMGKVGMCFHPDQDRILTVRECARSQGFP
DSYEFAGNIIHKHRQIGNAVPPPLASALGRKLKEAVDSKSST

>GmDRM1
MQFHNYVGSEGDDSSPSDYDWNTDDELEVFGIPPSDPTISSQESCEDSVGESSASCSSAKH
SKLIHLFVGMGFSRETVIKAIDENGRDNEEDIMEALLTLTAEKPLTVEKDEALSVLVNMGY
PFEEALTAIDKCGPKAHISELADFISASQLEKGLHSPQESPNNKHDASDYTHEKPCQPSGEY
YLHTSKKVKLGLGIFNEASQVISRKFPREVANKPYFYFENVALAPKGVWKTISRFLYEIEPE
YVDSKYFCAATRKRGYIHNLPTHNRSPLLPIPPLTIQEAFPTTKKWWPSWDRRTKLNCLLT
RVAPGPVTERIRKLLEKFGDEPPLHVQENVLVEIRKWNLVWVGKNKLAPLEPDEYEMLLG
FPRDHTRGGGVTRTERYKSLGNAFQVNTVAYHLSVLKGRFPNGINVLSLFSGIGGAEVALH
RLGMMLKNVVSVEIAEVNRNIIRSWWEQTNQRGNLIEVEDVQKVSSNELSQWITKFGGF
DLIIGGSPCNNISGSNRVSRHGLEGEQSSLFYEYFRIVEAVMEIQRDELL

>GmDRM2
MGGDDSGLESDNFDWNTEDELEIQNYNSSSSCLTLPNGDAVTGSGEASSSAVLANSKVLD
HFVSMGFSREMVSKVIQEYGEENEDKLLEELLTYKALESSSRPQQRIEPDPCSSENAGSSW
DDFSDTDIFSDDEEIAKTMSENDDTLRSLVKMGYKQVEALIAIERLGPNASLEELVDFIGVA
QMAKAEDALLPPQEKLQYNDYAKSNKRRLYDYEVLGRKKPRGCEKKILNEDDEDAEAL
HLPNPMIGFGVPTESSFITHRRLPEDAIGPPYFYYENVALAPKGVWQTISRFLYDVEPEFVD
SKFFCAAARKRGYIHNLPIQNRFPLLPLPPRTIHEAFPLTKKWWPSWDIRTKLNCLQTCIGS
AKLTERIRKAVEIYDEDPPESVQKYVLHQCRKWNLVWVGRNKVAPLEPDEVETLLGFPRN
HTRGGGISRTDRYKSLGNSFQVDTVAYHLSVLKEMYPNGINLLSLFSGIGGAEVALHRLGI
PLKNVVSVEKSEVNRNIVRSWWEQTNQKGNLYDMDDVRELDGDRLEQLMSTFGGFDLI
VGGSPCNNLAGSNRVSRDGLEGKESSLFFDYFRILDLVKNMSAKYR

>GmDRM3
MAGNPNRREGKTVMVPKTENLDYELPPYTSFSGDVGDNVASSSGGKLRAFFIGMGFLPCL
VDKVIEENGEENSDILLEALLRYSALQKSNSQSSVSLDSLFDDKDPPEISNVNQAKEEPDEL
SGVVDDTRGSLLMMNFSVEEVEFAIHKLGDEASIPELVDFIFALQIAKKLKKEPDDITFTYY
GRGNEVTNEKLFGIMAKTLQLFEMGFSENEVSSAIDKLGSEAPISELANFIFAEQNGIDYV
MEYKFPTTSTYSVGIKEEPEMDLYGTAEVKVEGFSHEPPQSSQVNLEETYNDDMVKEEEG
IDAFPSNVSDQYLDVVENGRGKRPKYEHDDDPVNCLEPSWVEERVDAVVAEMSRHPKPN
PSRCLSSVAAKPPFFLFGNVSNISYDSWTKMSQFLYGIEPEFANAQSFSAMDRIEGYIHNLP
VENRFHILPKPPMTIEDAMPQTKKWWPPWDSRKLLSSIYCETNGIAQTCDRLGNFLADSG
GVLTSEQQKDILRYCRRLNLVWIGKFKLGPVEPEQLELILGYPLNHTRATEGNVAERLKSL

KYCFQTDTLGYHLSVLRPIFPHGLTMLSLFSGLGGAEIALHRLAIKIKAVVSVETSETKRKI
LEKWWRQSGQTGTLVQIEDIQKLTSKKLEGLISKFGGFDLVIYQNPCSYSSSRLQAGVGLS
ALDFSVFCECVRVLQRVRGMYQRK

>GmDRM4
MAGNSNRREGKPVMVPKTEDLGYELPPYTSFSGDVGDNVASSSGGKLRAFFIGMGFLPCL
VDKVIEENGEENSDTLLEALLRYSAHKSNCDSFDSLGVSHNTSRGRSAPNFYPDGHSKEA
LQKSNSQSSDSLDSLFDDKDPPEISNVNQAKEEPDELSGVIDDKRGSLLMMNFSVEEVELA
IHKLGDEASIPELVDFIFAWQIAKKLKKEPDDITFTYYGRGNEVTNEKLFGIMAKTLQLFE
MGFSENEVSSAIDKLGSEAPISELANFIFAEQNGIDYVMEYKFPTTSTYSVGIKEEPEMDLY
GTAEVKVEDFSNEPPQSSQVNLEETYNEDMVKEEEGIDAFPSYVSDQYLDVVENGRGKRP
KYEHDDDSITCLEPSWVEERVDVVVAEMSRRPKPNPSRCLSSVAAKPPFFLFGNVSNISYD
SWTKMSKFLYGIEPEFANAQSFSALDRIEGYIHNLPVENRFHILPKPPMTIEDAMPLTKKW
WPPWDSRKQLSSIYCETNGIAQTCDRLGNILADSGGVLTSELQKDILRYCRGLNLVWIGKF
KLGPVEPEQLELILGYPLNHTRASEGNVAERLKSLKYCFQTDTLGYHLSVLRPIFPHGLTM
LSLFSGLGGAEIALHRLGIKIKVVVSVETSETKRKILERWWRQSGQTGTLVQLEDIQKLTSK
KLEGLISKFGGFDLVIYQNPCSNLSSRLLAGVGLPALEFSVFCECVRVLQRIRGMYQRK

>GmDRM5
MGGDDFDWNTDDELEIENYNSSSSCLTLPNGDAGEASSSAVLANSKVLDHFVNMGFSRE
MVSKVIQEYGEENEDKLLEELLSYKALESSPQPQQRIEPDPCSSENAGSSWDDFSDTDIFS
DDEEIAKTMSENDDTLRSLVKMGYKQEEALVAIERLGPNASLEELVDFIGVAQMAKAEDA
LLPPEEKLQYNDYAKSNKRRFYDYEVLGRKKPRGCEKKILNEDDDEEDEALHLPNPMIGF
GVPTESSFITHRRIPEDAIGPPYFYYENVALAPKGVWQTISRFLYDVQPEFVDSKFFCAAAR
KRGYIHNLPIQNRFPLLPLPPRTIHEAFPLTKKWWPSWDTRTKLNCLQTCIGSAKLTERIRK
AVEIYDEDPPESVQKFVLHQCRKWNLVWVGRNKVAPLEPDEVETLLGFPRNHTRGGGISR
TDRYKSLGNSFQVDTVAYHLSVLKEMYPNGINLLSLFSGIGGAEVALHRLGIPLKNVVSVE
KSEVNRNIVRSWWEQTNQKGNLYDIDDVRELDGDRLEQLMSTFGGFDLIVGGSPCNNLA
GSNRVSRDGLEGKESSLFFDYFRILDLVKNMSAKYR

>OsMET1
MDKCGDSDDCISTSEAAERAAKLSEDKIKNLPVPGEVEFINGGPPCQGFSGMNRFNQSPW
SKVQCEMILAFLSFAEYFRPRFFLLENVRNFVSFNKGQTFRLTLASLLEMGYQVRFGILEA
GAYGVAQSRKRAFIWAAAPGETLPEWPEPMHVFASPELKITLPDGKFYAAVKSTAAGAPF
RSITVRDTIGDLPAVENGAGKPTIQYGSGPVSWFQKKIRSDMASLNDHISKEMNELNLIRC
KHIPKRPGCDWHDLPDEKVKLSTGQMVDLIPWCLPNTAKRHNQWKGLYGRLDWEGNFP
TSVTDPQPMGKVGMCFHPEQDRIITVRECARSQGFPDSYRFAGNIQNKHRQIGNAVPPPLA
YALGRKLKQAIDAKR

>OsMET2
MDKCGDADDCISTSEAAEQAAKFSQDNIMNLPVPGEVEFINGGPPCQGFSGMNRFNQSP
WSKVQCEMILAFLSFAEYFRPRFFLLENVRNFVSFNKGQTFRLTVASLLEMGYQVRFGILE
AGTFGVAQSRKRAFIWAAAPGETLPDWPEPMHVFASPELKINLPDGKYYAAAKSTAGGAP
FRAITVRDTIGDLPKVENGASKLLLEYGGEPISWFQKKIRGNTIALNDHISKEMNELNLIRC

QRIPKRPGCDWHDLPDEKVKLSSGQLVDLIPWCLPNTAKRHNQWKGLYGRLDWEGNFPT
SVTDPQPMGKVGMCFHPDQDRIITVRECARSQGFPDNYQFAGNIQSKHRQIGNAVPPPLAF
ALGRKLKEAVDAKRQ

>OsCMT1
MVPEPAPAAATEPRRSTRRRLMTAAAMEAEAEAVADLDEIDREMSRAESRKRQRRTAKEK
PGARKGATEWKPEDVEKAAAAEGVAELDEIDREMPRPELRKRQRRTAKEKPSAHEGATE
WKPEDVEKAAAQEPEGTELDSGLSPAESRGKRQRGVEKVKRRTRKKTAKEKTKETTEKS
AAQAPEKMKVNDAGGALAEDVCADEPDAEQMAMEEEEEAADVLEAEERMGKCVGEGS
AEKAATRKRVARPSTARRVEDSDDHFVGDPVPDDEARQRWPVRYSRKGSDSLLKQEPDE
DEEMKARCHYLAANVDDEIYHLDDDVYVKAGPDEENYIGRITEFFEGVDRGSYFSCQWF
FRTADTVISSKLLKVHDHRHNHKRVFLSKEKNDNLIECIVSKVKIAHVDPNMTPQARAHAI
SDCDLYYDMSYSVAYSTFANLPADNDGALGSEATSNISCDDADNSSKGKLSADIVAPYSEQ
TETASLLDLYSGCGAMSTGLCLGFAFSGINLETRWAVDINKYACACLKHNHPYSQVRNEK
TEDFLALIQQWDALCRKYVVHKNDTLEPSIDMPLNDADDVNEPLPEDIFDVEELLEICYG
DPSNTGKNGLWFKVRWKGYDPSYDTWEPIDGLSDCPERIKEFVEKGHKENILPLPGAVDV
ICGGPPCQGISGFNRFRKHNDPLEDEKNKQLVVFMDIVKYLRPKYVLMENVVDILKFADG
FLGRYAMSCLVAMNYQARLGMMAAGYYGLPQFRMRAFLWGALPSMVLPKFPLPTHDAV
VRGIVPTTFSQSVVAYNEVDTRCLRKALLLADAISDLPKVGNDQPKDVIEYSVAPKTEFQR
YIRNNRKDIQDYSFRGDDPSEEGKLFDHQPLKLNKDDYERVQRIPVKKGANFRDLKGVIV
GPDNTVRLDPNISRERLSSGKPLVPDYAISFVKGKSTKPFGRLWWDETVPTVVTRAEPHNQ
IILHPSQDRVLTIRENARLQGFPDYYRLIGPLKEKYIQVGNAVAIPVARALGYALGLAYRGE
SDGDRAVLKLPESFIYADQETVVKSSAGTPGSEIADSEQLFEYFINPNFFCMMFAFLKVNV
VIPLILDSKMGLSRRGAEEGEARRAVPFQNILSWEGWNRLDHRRGEIRLDVRREMDDSPL
DNLFDGNGLDSSAGADSSSSTGSPPSSTSSSPPSSQSPPPGSSPPPASPPPSTPSAPPTNSSGSA
PSPPSPSQSAPPANTGGGGSPPPSHGSPPAPKAVQSQPAPKRSGDGGSSSDSGSSKEGGSSSD
RGKSESNGNRPGPEAAIIAGMVIGFFTFALLLAIVACVCCSKKKKRPPHMHMPYYTDENG
KVYYANSMPRWQNSVDQGGGWHAQYSPGQAPPSSEMSGSHGAGPLPPPSPGMALGFSK
SSFSYDELALATGGFSSANLLGQGGFGYVYRGVLAGSGKEVAVKQLKAGSGQGEREFQA
EVEIISRVHHRHLVSLVGYCIAGSSQRLLVYEFVPNDTLEHHLHGLHIFPALFPAIVTRHSEG
RAGDGLDDEARHRARLSQGPRVPSRRFLQGQILSVRPSNDLICCIVVVTYEGHPRIIHRDIK
AANILLDENFDAKVADFGLAKLTTDTNTHVSTRVMGTFGYLAPEYASSGKLTDKSDVFSF
GVMLLELITGRRPVDPTNYMEDSLVDWARPLLARALSEDGSFDELIDQRLENKFDRLEME
RMAACAAAAVRHSAKRRPKMKQIVRALEGDASLDDLNEGVKPGQSMMFSTGSEYDSGN
YASDINRLRKVAFESSIEDSSEYGTHSSADSGEPPRRQQHR

>OsCMT2
MEASVSQIRRSPPRIEKRKKKNHNSLSGKAPPTRGNCDSLPPVWNAKSASECHWTRRLTS
NYLSLVVVVVGEELPPCFAPPPMETPPPDPVSPPPPAADEGSPGGDDGAEDAGGFSAGLDS
LWTALFGSPEELEPMWSPPRGFGVGAEFAAAEVEPEIMDVAGGPWDGAPWRSSGVVAGE
GAATALVPPTAAAGFAEFEPAAPIDSYPAGAAAASLGDVPEVSALDSGVDCSPDPPPSSSPP
VDFDARGFDPVADSAPAMESPLPPSVASSEANLDGRMLDCTLNSVPSPPLASPYEVGLGAE
DPIKDSSPSVAWGTTMDAKDPEVDATCANGTALRRSRRIMKIKSAASSMPLNQNGDSSRA
SKRRVADSRKSRSSEGSKLPAFTGPISVNTVDLINGVKVQGLQEIVAVENVSSSYDNNQKA

GGLYNQVVVALPAASNSLLKDKGASVLPRRKTRLASKVLVNSDRVSAISPVVNGGPPVQK
SDVCIPTKKHKLAVEECLTSLDGVDGGGIVLCNSKLKSAKSRVVSKTPQGRGRRSPQPPKT
QRARTLSVKYLEKLKRAENNNNNGSMSKSPRVPMIPENNGSMSKSPRVPIIPELSTKHELV
LDKHMVDSVMLETDDGSCFFVGDAVPDDEARKQWPHRYEINDQIMKKDKRTSSQTFAKL
VTVSFCVVFMYVYLQMLDFRGPEGKPNYIGRLLEFFETKTGECYFRVQWFFTAEDTVIGE
QAQSHDPRRLFYSDLTDDNLLDCIVSKVTIVQVPPSVDGKSKSVPSSDYYYDMKYSIDYST
FSTIEMEDTDDLMQSCYTSRINDKMKKIDVNKKHKSPVLEKMELSLLDLYCGCGGMSTG
LCLGARGGGVNLSARWAIDDDEIACESFRNNHPETRVRNETTDDFLELLKEWEKLCKTYV
KHSRTKACVDSTTESNNETPDCSTVPPEEFEVWKLVDICFGDPNKVSKHGLYFKVRWKGY
GPHHDTWEPVEGLRNCKEAIRDFVIEGHRQRILPRPRRNIAVFLLRPSKFPLGDVDVVCGG
PPCQGISGYNRNREFEAPFKCEKNKQIIVFMDVVQFLKPKYVYMENVLDILKFADATLAR
YALSRLVAMHYQARLGIMAAGCYGLPQFRMRVFLLGCHSKEKLPPFPLPTHEAIVKNGCP
LAFERNLVGWPNDTPMQLARPIVLEDILSDLPEVANGESRDEMLYVKGPQTEFQRYIRSFN
VEVHGPRAHVTKDSKSSKLYDHRPLVLDNDNYQRILQIPKRKGANFRDLSGVIVGPDNVA
RLDPTKERVLLPSGRPLVLDCILAYENGKSLRPFGRVWWDEVVGTVLTVPNARMQALIHP
AQDRLLTIRESARLQGFPDNYRFRGTVKDRYRQIGNAVAVPVGRALGYALAMAYLKKSGD
DPLMLLPPNFAFSHDLRGFA

>OsDRM1a
MRIASSSGILMDANGKANGSAPSALVAYFLGMGFSREMVFRAIKEIGDTDSEQILELLLTY
QAIGSDPSVGNSSHSACDPQILEEEDEEEDVNWDEDDTVDNFDRATYSDGSGDEDFLQEM
SEKDEKIKSLVSMGFPEDEDTEFSSFGGRKKTKLIDGSKKKRERYRSRPQWNQVPFDGSHE
EPMPLPNSMVGFSLPNDGLRSVHRNLPDQALGPPFFYYENVALAPKGVWTTISRFLYDIYP
EFVYSKYFCAAARKRGYIHNLPIKNRNYTRGVSRTARYRALGNSFQVDTVAYHLSVLRDI
FPNGMNVLSLFSGIGGAEVALHRLGICMKTVVLVEISEVNMTLLRSWWDQTQTGTLIEIA
DVQNLTAERIELFIRRFGGFDLVIGGSPCNNLAGSNRYHRDGLEGKHSALFYHYYRILDSV
KTIMASIFGAKGKLFRHVRKALLLKQSSSLTLKTEQDPSNNSDKDSMDK

>OsDRM2
MQAIGGDASVGNCSASACAPQTLEVDEEEDDTNWDEYDTAGNCDRTPHSDGSGDEDFFQ
EMSEKDEKMKSLVNMGFPEDEAKMAIDRCLDAPVAVLVDSIYASQEAGNGYSANLSDYE
DTEFSSFGGRKKTRFVDGSKKRKRYGSGPSGNQVPFDGSHEEPMPLPNPMVGFSLPNERL
RSVHRNLPDQALGPPFFYYENVALAPKGVWTTISRFLYDIQPEFVDSKYFCAAARKRGYIH
NLPIENRSPVLPMPPKTISEAFPNTKRWWPSWDPRRQFNCLQTCMASAKLTERIRCALGRF
SDVPTPQVQKYVLDECRKWNLVWVGKNKVAPLEPDEMEFLLGYPRNHTRGVSRTERYR
ALGNSFQVDTVAYHLSVLRDLFPNGMNVLSLFSGIGGAEVALHRLGIHMKTVISVEKSEV
NRTILKSWWDQTQTGTLIEIADVRHLTTERIETFIRRFGGFDLVIGGSPCNNLAGSNRHHRD
GLEGEHSALFYDYIRILEHVKATMSAV

>SlMET1
MASPQPNSESVLELPNNDKSGHKKNKRKQDSVSKRKASATGKKEKKQAVSETIEEPTAGR
KRPKRAAACSDFKEKSVHLSKKSSVIETKKDHCVDEEDVAIRLTAGLQESQRPCRRLTDFV
FHNSEGIPQPFGMSEVDDLFISGLILPLEDSLDKVKAKGIRCEGFGRIEEWAISGYEDGTPVI
WISTETADYDCLKPSGSYKKFYDHFLAKATACVEVYKKLSKSSGGNPDLSLDELLAGVVR

AMTGIKCFSGGVSIRDFVITQGGFIYKELIGLDDTSKKTDQLFVELPVLASLRDESSKHETL
AQPETISSGNGLRIGPKAGNGGDKIVESGLANGPAPEDEDLKLAKLLHEEEYWCSLKQKK
DRNTSSSSSKIYIKINEDEIASDYPLPAYYKTSNEETDEYIVFDSGVETYHIDELPRSMLHNW
ALYNSDSRLISLELLPMKACADIDVTIFGSGVMTADDGSGYNFDTDANHSSSGGSRSAEID
GMPIYLSAIKEWMIEFGSSMIFISIRTDMAWYRLGKPLKQYAPWYEPVIKTARLAVSIITLLK
EQNRVARLSFGEVIKRVSEFKKDHPAYISSNVDAVERYVVVHGQIILQQFSEFPDVSIRNCA
FAVGLSRKMEERHHTKWVIKKKKVMQRLEQNLNPRASMAPSVKRKAMQATTTRLINRI
WGEYYSNYSPEVSKEVADCEVKDDEEPDEQEENEEDDVPEENLDVPEKAHTPSSTRRHIK
SRSDSKEINWDGESIGKTASGEQLFKKARVHGHEIAVGDSVLVEHDEPDELPSIYFVEYMF
EKLDGSKMLHGKMMQRGSDTVLGNAANEREVFLINECMNLQLGDVKESIAVNIRMMPW
GHQHRNTNADKLERAKAEDRKRKGLPTEFYCKSFYRPEKGAFFRLPFDKMGLGNGLCYS
CELQQTDQEKESFKFDMSKSSFVYLGTEYSVDDFVYVSPDHFTAERGGNGTFKAGRNVG
LMAYVVCQLLEIVGPKGSKQAKVDSTNVKVRRFFRPEDISSDKAYSSDIREIYYSEDIHTVP
VEIIKGKCEVRKKYDISSEDVPAMFDHIFFCEYLYDPLNGSLKKLPAQINLRLSKIKLDDATS
RKRKGKGKEGVDEVGELNETSPQNRLSTLDIFAGCGGLSEGLQHSGVTDTNWAIEYEAPA
GDAFRLNHPKTKVFIHNCNVILRAVMQKCGDSDDCISTPEASELAAAMDESELNSLPLPG
QVDFINGGPPCQGFSGMNRFNQSTWSKVQCEMILAFLSFADYYRPKFFLLENVRNFVSFN
QKQTFRLTVASLLEMGYQVRFGILEAGAYGVPQSRKRAFIWAASPEEVLPEWPEPMHVFA
VPELKIALSETSYYAAVRSTASGAPFRSLTVRDTIGDLPVVGNGASKTCIEYQGDPVSWFQ
KKIRGSSITLSDHISKEMNELNLIRCQRIPKRPGADWRDLEDEKVKLSNGQLVDLIPWCLP
NTAKRHNQWKGLFGRLDWDGNFPTSITDPQPMGKVGMCFHPDQDRIVTVRECARSQGFP
DSYQFAGNILHKHRQIGNAVPPPLAYALGRKLKEAVESKNRLT

>SlDRM7
MDNNFSGEDNDSIDWDTEDELEIQEMPDATFSSCTNLRSVGYHTVSGHREARSSSEPCQS
KFIQQFIVMGFPEESIAKAIEQNGENEGLVLDALLTFKALEDSPEEQPSTSTQMEPCITSDDS
SSQYNENFLDDVSEDDSWSLDSDNCVNSAKQSYLNDDNCSLSENEKTLLFLANMGYPAE
EASIAMERCGPEAPFPELIDFMCAAQMAREEDVHLPEDEKPKLNSGGYKRKMYNEVRVK
KKQRAITDEETIHLPRPMIGFGVPTESLPAVVKRTLPEQAIGPPFFYYENVALAPKGVWDT
MTRFLYDIEPEFVDSKYFCATARKRGYIHNLPIEDRFPLLPLPPRTIHEAFPLTKKWWPSWD
TRTKLNCLQTSIGSARLADRIRKAMKAMENFDSEPPLMVQKYVLDECRKWNLLWVGRN
KVSPLETDEFEMLMGFPRNHTRGGGISRTDRYKSLGNSFQVDTVAYHLSVLKDLYPNGIN
VLSLFSGIGGAEVALYRLGIPLNNVVAVEISEVNRNILRSWWEQTNQKGNLIDFHDIQQLN
GDVLEQMIDSIGGFDLVIGGSPCNNLTGSNRVTRDGLEGKDSSLFYDYVRVVDLVKSIMSN
RRV

>SlDRM6
MDKNLSGEDNDDIDWDTEDELEIQEIQDTVFSSCTDLRTTGQHVVCCDVEASSSSVPFRS
KFIQQFVVMGFPEESIAKAIEQNGENSDLVLDSLLTFKALDDSPEEQPSVSPPLEPSISSDDS
ASEYNKIVLDNVYEDDSWSSDSDNYINTVKQCYLNDEGSSLSEKEKMLLFLGNMGYPAE
EASIAMDRCGPKASLPELVDFICAAQMSRAEDPYLLEDVKPNLKDILNDCGGYKKRKMY
NELCKRKKQREISVEEPIRLPKPMIGFGIPTESVPRMVQRILPEKIIGPPYFYYENVALAPKG
VWDTIKRHLYEIEPEFVDSKYFSATARKRGYLHNLPIENRFPLFPLPPRTIHEALPLSKRWW
PSWDTRSKLNCLQTAIGSARLTDKIRKAVEKYDGEPPMEIQKYVLYHCKKWNLVWVGRN

KVAPLEPDEVEMLLGFPKNHTRGGGISRTDRYKSLGNSFQVDTVAYHLSVLRDLFPNGINV
LSLFSGIGGAEVALYRLGVPLNNVVSVEKSEVNRNIVRSWWEQTNQRGNLIHFDDVQLLS
RDRLKKLIESVGGFDLVIGGSPCNNLAGSNRVSRDGLEGKESSLFFDYVRILDDVKSIMSR
HR

>SlDRM5
MDKHLSEEDSDNIDWDTEDELEIQDTTFSSCRDLRTNGQYAISGDGEASSSSVPGQSTFIQ
KFLVMGFSEESIAKAIEQNGENSDLVLDALLTLKAIEDSPEEQPSASPHLEPCINSDDSSSEY
NENFLDDVYDEDSWSSDSDYCTNSVKQCYVKEESNSLSEKEQTILFLANMGYPVEEVSIA
MERCGPEASVSELTDFICAAQMAREEDPYLPEDVKPKLNHGSGGYKKRKMFNQLCKSKK
PRAIFDEETIRLPKPMIGFGVPTESVSAIVRRTIPEQAFGPPFFYYENVALAPKGVWDTISRF
LYDIEPEFVDSKYFCATARKRGYIHNLPTENRFPLLPLPPRTINEALPLTKKWWPSWDPRTK
LNCLQTAIGSARLTDRIRKAVEAFDGEPPMRVQKFVLDQCRKWNLVWVGRNKVAPLEPD
EFEMLLGFPKNHTRGGGISRTDRYKSLGNSFQVDTVAYHLSVLKDMFPNGMNVLSLFSGI
GGAEVALYRLGIQLNNVVSVEKSEVNRNIVRSWWEQTNQRGNLIDFDDVQQLNGDRLEQ
LIDSCGGFDLLIGGSPCNNLAGSNRVSRDGLEGKESSLFYDYVRILDLVKSIMSRQR

>SlCMT4
MAKKQNDSSRFSTNLTPSPQKDKPISSSSELALFYPQNEEPVPLIVFYPSSVRRRSSRFTTNK
FSTSATEFMTKNRDTISPEKTVFLLPSPPTLAGKKTPAEVTRRSPRLVSLSARTTTAKEKGK
KVNSRKSEGGIKQVELSRKRKPASCKMDEETRKSPRFNSDSSNGVQLALPEMSACGALSA
GGRTGTKRELLALMTTTPANSSASRKRAARGSDSVNVGNNSRGSRRKDPVFAESPGTSVK
ITAESNSAGEKNLRSRKAQGSVNYNESKGSETKRIKSSAEKSVRKQKSNACFIGEPIDTEEA
QQQWQWRYELKNRKTQRQGWKLNSGEEDEIILNVECHYAQAKVAGFIFNIGDCAFVKGE
GKKKHIGRILEFFKTTEGEDYFRVQWFFRAEDTVLQGAASFHDPKRVFYSTLENDNLLDCI
VSKVNVVELPTRHDLNKKDVPPAHFYYDMEYCVDYSTFRTLHNVKSSVSPSLVDVSYKPI
TTYPLEVSPSCEPMKVELSLLDLYAGCGGMSMGLCLGTKLSGLNLVTKWAVDFNKAACD
SLKLNHPQTHVRNEGVENFLELLKRWEKLIKSYGCSDIKTSSNIELDDRDEGENNDDSQS
GSNASSGEYEVLRFVDICYGDPNNDGKSGLHFKVRWKGYGPSEDTWEPIENLKNCGDSIK
DFVRRGQQLKILPLPGDVDMICGGPPCQGISGYNRHRNTDDPLSDEKNRQIIIFMDVVEFL
RPKYVLMENVADILRFDKASLGRYALSRLVHMRYQARLGTMAAGCYGLPQFRLRVFFWG
ALPSERLPPFPLPSHDVIVKYWPSPEFERNTVAYEEGQPRDLEEALVLRDAISDMPAVTWH
ETREERPYEMPPETEFQKYIRLSKHEILSCTSTGVKETKEPVLSDHRPCQLNEDDYLRVCLV
PRRKGANFRDLPGVIVGGDNVARRDTKDPKVLPNGKPMVPDCAFNFEHGKSKRPFARLW
WDETVATLVTFPNHRAQAILHPEQDRVLTIREYARLQGFPDFYRFTGTLKERYCQVGNAVA
VPVGRALGYALGLAYQRLAGNEPLIKLPSNFSFLTPPIDDIVVLQT

>SlCMT3
MSSKRKASPADSSSDSSKRHALEVVKTVDIASDEVAEGFRDDDEFVEDRDIVCDSSIGESS
GQKEVRRVAVRANEEQEGEFYGEIVLDSEARKKWPHRYILKDNVNINSASMSLNCQHDSD
ELIQAKCHFAQALVDNVIYKLGDDAYVKAAEDEDDYICKIVEFFQGVDDMKYFTAQWFY
RAKDTVIKAHDQFIDKKRVFLSDIKDHNPLDCLVKKIKIVPISSNVSLQFKESLRLECDYYY
DMKYLVPFSSFISLPSDVLSPDSESNSTISSDGDVVEVKEQKQEKKLLDLYSGCGGMSTGL
CLGADVCDVKLVTKPSNTPTHPLLKVGDANVEDDDEGADDDDGGSGDEDEGEIFEVEEI

LEVCYGDPKEIKKPGLYFKVRWKGYGPDEDTWEPIEGLDGCQNKIKDFVTDGFKRSVLPL
PGQVDVVCGGPPCQGISGFNRFRNSANPLQDPKNKQLEVFMSIVEFLKPRFVLMENVVDL
LRFAHGYLGRYALSRLVGMNYQARMGMMVAGAYGLPQFRMRVFMWGALPSEKLPQYP
LPTHNVIVRGGIPTEFELNAVDFEEGLKVKLKRELLLEDALSDLPPVENNEPRDEMPYIDEP
KSVFQRFIRSRRDGTLGTVLYDHRPLQLNEDDYQRVTQIPKQKGANFRDLPGVRVRADNV
VEWDPDMERVKLPSGKPLVPDYAMTFVRGTSQKPFGRLWWDEIVSTVVTRAEPHNQAIL
HPVQDRVLTIRENARLQGFPDYYKLTGPIKERYIQVGNAVAVPVARALGYSLALALKGLSR
DQPLLTLPPNFPCLEELVSNDESLDKV

>SlCMT2
MPSKRKSSPATKPESSSGSRKSKRLVVERPDPVVAQPSDSDFEPEPVLSSKKKSTRRTTAES
SVVACQSESNNKKLKKPTVEKAESGVASPADRDFVSESDSETPSKKSTRRAAVKVEPLVDS
VAGSDFVEEEEVDGMELGSLKKSLSISPSKRKPKRAEKVKDEECVLAGDPVPDAEARLK
WPHRYNKGKENGTKSLNGQDDPDQLIQAKCHFSRADVDGQIYYLEDDAHVKAADGEDD
YICKIVEFFEAVDGVQYFTAQWFYRAKDTVIKSHDQFIDKKRVFLSEIKDDNPLDCLVTKL
KIVPVPSNATSQFKENVKSNCDFYYDMKYLLPYSSFISLPPDTTSPVSSSSTISSDIDAGEVK
EHNLEKKLLDLYSGCGAMSTGLCLGANSKGVKLVTKWAVDLNKHACDSLRLNHPETQV
RNEYASDFLSLLKEWVQLCVSCSLIKGSVPPHPHLKVTDEVDEDEENDDEGEDSGDDKEG
EIFEVEELLEVCYGDPKENNKPGLYFKVRWRGYGPEEDTWEPIDGLSDCPKKISEFVVKGF
KANLLPLPGDVDVVCGGPPCQGISGFNRFRNKENPMQDPKNKQLDVYMDIVDFLKPRFV
LMENVVDLVKFSNGFLGRYALSRLVGMNYQARMGMMAAGAYGLPQFRMRVFMFGALS
SEKLPQYPLPTHKVIVRGVIPVEFESNTVAYDSVRDLELKKELFLGDALSDLPLVENNEPRD
EMPYTDEPKSDFQHFIRMGRDGLLGSVLYDHRPLQLNEDDHQRVCQIPKRKGANFRDLPG
VRVRPDNKVEWDPDVERVKLPSGKPLVPDYAMSFVGGSSSKPFGRLWWDETVPTVVTRA
EPHNQTIVHPLQDRVLTIRENARLQGFPDYYKLIGPIKERYMQVGNAVAVPVARALGYSLA
MSIKGLSGETPLFTLPKNFPSHEDQNCNEVSQ

>FaMET1a
MGSIAAADDAAAAAAATAPLNDAESAPSALSSGTTKKKGRKATQKEAPAGGRGRKRNVP
PTGEEQSQSRKMPKRAAACKDFKERSVRVSEKSAFIESKEEQVVPDESLAVVMTCGKDPD
DEKPNRRLTQFILHNEDGVAQPLEMVQHGDLFITGTLLPLDASSDNGKGKGKEKGIICEGF
GRIEAWDISGYDEGHPIIWLTTDVADYCCVKPASSYKKHYDNFCEKARACIEVYKVLSKC
NSDCSLDELLAGIARSMSGNKFFSGSASIKEFIISQGVFIYNQLEGLEESSKKNDRVLAQLP
VLAALRQECIKQGYYVTSNPAASSGTLKIASDGGNSSSKVETEEDEDAKLARLIQEEEYFK
SMKQKKRQGVTSMSKKFYIKINEDEIANDYPEPAYYKTAIEETDEFIVVDSEDGDLPTHML
HNWCLYNSDSRLISLELLPMKACADIDVTIFGSGVMSEDDGSCGFDLDSIQSSSSGSVAPVA
EDAYGMPVYLSSIKEWVIEWGASMIPVSIRTELAWYRLGKPSKQYAKWYAPILKTARVGR
SIITMLKAESRVARLSFADVIKRLSEFPNSNGGYISSDPASVERYVVVHGPILLQLFTEFPDG
KIKTCPFVHGLRDKMEKRHHTKWEVNKKILQKSESNLNPRASMGPVVSKRKAMQATTT
KLINRIWGEYYSNYSPENSSLVETCEKIEEDEADEEVQEDLEEDDAEENSSVVKEAQRPSPI
LRKIKSSSSSKKGFVDEEPVGKTSSGEALYKHAVLRGEEISVGGAVMVEVDGSDELPVIYF
VEYMYESSDGEKMLHGRMMQRGSDTVLGNTANEREVFLTNECINLALKDVKQTVVVGI
KSMPWGHKHQKENADAERIDRANAEERKKKGLPTEYYCKSLYCPEKGAFLSLSCDTIGL

GSGFCFSCKANEEEKAKEVFEVNSSKTGFIYSGVEYSVHDYVYVSPYHFSVQTIETELFKA
GRNLGLKPFVVCQVLGIITKGSKQSEMKSTQVKVRRFFRPEDISVEKAYCSDVREVYYSEE
LHILPVDSIEGKCEVRRRSDLPECNAPALFQHIFFCEHLYDPSNGSLKQLPANIRVKYSTVG
GDTESRKRKGKCKEGEDVPEVEQQRVDSEHMRLATLDIFAGCGGLSEGLHQAGVSITKW
AIEYEEPAGQAFQLNHPESKVFINNCNVILKAVMDKCGDTDDCISTTDANDLASALDEKE
KSDLPLPGQVDFINGGPPCQGFSGMNRFNTSTWSKVQCEMILAFLSFADYFRPKYFLLEN
VRNFVAFNKGQTFRLTLASLLEMGYQVRFGILEAGAYGVSQSRKRAFIWAAAPDEILPEW
PEPMHVFGVPELKINLSSNSYYAAVRSTASGAPFRPITVRDTIGDLPAVGNGASKVNMEYE
SDPVSWFQKKIRGNMAVLTDHISKEMNELNLIRCKRIPKRPGADWKDLPEEKVKLSTGQL
VDLIPWCLPNTAKRHNQWKGLFGRLDWEGNFPTSITDPQPMGKVGMCFHPDQDRIITVR
ECARSQGFPDSYQFYGNTLHKHRQIGNAVPPTLAYALGRKLKEAIDSKKRSSSQE

>FaMET1b
MGSIAAADEAATAPLNDAESAPSAPSSGTTKKKGTQKEAPAGGRGRKRNAPPTGEGQSQS
RKMPKRAAACKDFKERSVRVSEKSAFIESKEEQVVPDESLAVVMTCGKDPDDEKPNRRLT
QFILHNEDGVVHPLEMVQHGDLFITGTLLPLDASSDNGKGKGKEKGVICEGFGRIEAWDIS
GYDEGNPIIWLTTDVADYCCVKPASSYKKHYDNFCEKARACIEVYKVLSKCNSDCSLDEL
LAGIARSMSGNKFFSGSASIKEFIISQGVFIYNQLEGLEESSKKNDRVLAQLPVLAALRQECI
KQGYYVTSNPAASSGTLKIASDGGNSSSKVETEEDEDAKLARLIQEEEYFKSMKQKKRQG
VTSMSKKFYIKINEDEIANDYPEPAYYKTAIEETDEFIVVDSEDGDLPTHMLHNWCLYNSD
SRLISLELLPMKACADIDVTIFGSGVMSEDDGSCGFDLDSIQSSSSGSVAPVAEDAYGMPVY
LSSIKEWVIEWGASMISVSIRTELAWYRLGKPSKQYAKWYAPILKTARVGRSIITMLKAESR
VARLSFADAIKRLSEFPNSNGGYISSDPASVERYVVVHGPILLQLFTEFPDGKIKTCPFVHGL
RDKMEKRHHTKWEVNKKILQKSESNLNPRASMGPVVSKRKAMQATTTKLINRIWGEYY
SNYSPENSSLVETCEKIEEDEADEEVQEDLEEDDAEENSSVVKEAQRPSPILRKIKSSSSSK
KGFVDEEPVGKTSSGEALYKHAVLRGEEISVGGAVMVEVDGSDELPVIYFVEYMYESSDG
EKMLHGRMMQRGSDTVLGNTANEREVFLTNECINLALKDVKQTVVVGIKSMPWGHKHQ
KENADAERIDRANAEERKKKGLPTEYYCKSLYCPEKGAFLSLSCDTIGLGSGFCFSCKANE
EEKAKEVFEVNSSKTGFIYSGVEYSVHDYVYVSPYHFSVQTIETELFKAGRNLGLKPFVV
CQVLGIITKGSKQSEMKSTQVKVRRFFRPEDISVEKAYCSDVREVYYSEELHILPVDSIEGK
CEVRRRSDLPECNAPALFQHIFFCEHLYDPSNGSLKQLPANIRVKYSTVGGDTESRKRKGK
CKEGEDVPEVEQQRVDSEHMRLATLDIFAGCGGLSEGLHQAGVSITKWAIEYEEPAGQAF
QLNHPESKVFINNCNVILKAVMDKCGDTDDCISTTDANDLASALDEKEKSDLPLPGQVDFI
NGGPPCQGFSGMNRFNTSTWSKVQCEMILAFLSFADYFRPKYFLLENVRNFVAFNKGQTF
RLTLASLLEMGYQVRFGILEAGAYGVSQSRKRAFIWAAAPDEILPEWPEPMHVFGVPELKI
NLSSNSYYAAVRSTASGAPFRPITVRDTIGDLPAVGNGASKVNIEYESDPVSWFQKKIRGN
MAVLTDHISKEMNELNLIRCKRIPKRPGADWKDLPEEKVKLSTGQLVDLIPWCLPNTAKR
HNQWKGLFGRLDWEGNFPTSITDPQPMGKVGMCFHPDQDRIITVRECARSQGFPDSYQF
YGNTLHKHRQIGNAVPPTLAYALGRKLKEAVDSKKRSSSQE

>StMET 1
MAIRLTAGLQDSQRPCRRLTDFVFHNSKGIPQPFGMSEVDDLFISGLILPLEDSLDKVKAQR
IRCEGFGRIEEWAISGYEDGTPVIWISTEIADYDCIKPSGSYKKFYDHFLAKATACVEVYKK
LSKSSGGNPDLSLDELLAGVVRAMTGIKCFSGGVSIRDFVITQGGFIYKQLIGLDDTSKKT

DQLFVELPVLASLKDESSKQETLAQPEHISSGKALHIGPKAGNGEDKIDESGLANGPAPED
ENLKLAKLLHEEEYWCSLKQKKGRNTSSSSSKIYIKINEDEIASDYPLPAYYKTSNEETDEY
IVFDSGVDTYHIDELPRSMLHNWALYNSDSRLISLELLPMKACADIDVTIFGSGVMTADDG
SGYNFDTDANHSSSGGSRSAEIDGMPIYLSAIKEWMIEFGSSMIFISIRTDMAWYRLGKPLK
QYAPWYEPVIKTARLAVSIITLLKEQNRVARLSFGEVIKRVSEFKKDHPAYISSNVDVVERY
VVVHGQIILQQFSEFPDASIRNCAFAIGLSMKMEERHHTKWVIKKKKVMQRLEQNLNPRA
SMAPSVKRKAMQATTTRLINRIWGEYYSNYSPEVSKEVVDCEVKDDEEADEQEENEEDD
VPEENLDVPEKAHTPSTRRHIKSCSDSKEIKWDGESIGKTASGEHLFKRARVHGHEIAVGD
SVLVEHDEPDELPSIYFVEYMFEKLDGSKMLHGRMMQRGSDTVLGNAANEREVFLINEC
MNLQLGDVKESIAVNIRMMPWGYQHRNTNADKLDRAKAEDRKRKGLLTEFYCKSFYSP
EKGAFFRLPFDKMGLGNGLCYSCELQRTDQEKESFKFDMSNSSFVYLGTEYSVDDFVYV
SPDHFTAEREGSGTFKAGRNVGLMAYVVCQLLEIVGPKGSKQAKVDSTNVKVRRFFRPE
DISSVKAYTSDIREIYYSEDIHTVPVETIEGKCEVRKKYDISSEDVPAIFDHIFFCEYLYDPLN
GSLKKLPAQIKLRFSKIKLDDATSRKRKGKGKEGEDEVGELNETSPQNRLATLDIFAGCGG
LSEGLQHSGVTDTNWAIEYEEPAGEAFRLNHPKTKVFIHNCNVILRAVMQKCGDSDDCIST
PEASELAAAMDENELNSLPLPGQVDFINGGPPCQGFSGMNRFNQSTWSKVQCEMILAFLS
FADYYRPKFFLLENVRNFVSFSQKQTFRLTVASLLEMGYQVRFGILEAGAFGVPQSRKRAF
IWAASPEEVLPEWPEPMHVFAVPELKIALSETSHYAAVRSTASGAPFRSLTVRDTIGDLPVV
VNGACKTCIKYQGDPVSWFQKKIRGSSITLSDHISKEMNELNLIRCQRIPKRPGADWRDLE
DEKVKLSNGQLVDLIPWCLPNTAKRHNQWKGLFGRLDWDGNFPTSITDPQPMGKVGMC
FHPEQDRIVTVRECARSQGFPDSYQFSGNILHKHRQIGNAVPPPLAYALGRKLKEAVESKK
RLT

>StCMT2
MPSKRKSSPATKPESSSGSRKSKRLVVERPDPVVAQPADSDFEQEPVLSSKRKSTRRTTAES
SVVACQSESNNKKLKKPAVEKVESGVASPADSDFVSESNSATPSEKSTRQTAVKVEPLVDS
VAESDFLEEEDVDGMELGSLKKSLSISPSKRKPKRAEKVKDEECTLAGDPVPDAEARLKW
PHRYNKGKSNGTKSLNGQDDPDQLIQAKCHFSRAEVDGQIYYLEDDAHVKAADGEDDYI
CKIVEFFEAVDGMQYFTAQWFYRAKDTVIKSHDQFIDNKRVFLSEIKDDNPLDCLVTKLKI
VPVPSNATSQFEENVKSNCDFYYDMKYLLPYSSFISLPPDTTSPVSSSSTISSDIDAGEVKEH
NLEKKLLDLYSGCGAMSTGLCLGANSKGVKLVTKWAVDLNKHACDSLRLNHPETQVRN
EYASDFLSLLKEWIQLCVSCSLVKSSVPPHPHLKVIDEVEEDEENDDEGDDSDDDKEGEVF
EVEELLEVCYGDPKENNKPGLYFKVRWRGYGPEEDTWEPIDGLSDCPKKIKEFVAKGFKA
NLLPLPGDVDVVCGGPPCQGISGFNRFRNKENPMQDPKNKQLDVYMDIVDFLKPRFVLM
ENVVDLVKFSNGFLGRYALSRLVGMNYQARMGMMAAGAYGLPQFRMRVFMFGALSSEK
LPQYPLPTHKVIVRGVIPVEFESNTVAYDSGRDLELKKELFLGDALSDLPLVENNEPRDEM
PYTDEPKSDFQHFIRMGRDGLPGSILYDHRPLQLNEDDHQRVCQIPKRKGANFRDLPGVR
VRPDNKVEWDPDVERVKLPSGKPLVPDYAMSFVGGSSSKPFGRLWWDETVPTVVTRAEP
HNQTIVHPLQDRVLTIRENARLQGFPDYYKLIGPIKERYMQVGNAVAVPVARALGYSLAMS
IKGLSGEAPLFTLPKNFPSHEEQSCNEVSQ

>StCMT4
MEYCVDYSTFRTLHNVKSSVSPSLVDASYKPITTYPLEVSPSCEPMKVELSLLDLYAGCGG
MSMGLCLGTKLSGVNLVTKWAVDFNSAACDSLKLNHPQTHVRNEGVENFLELLKRWEK

LIKSYGCSDIKTSSNGELDDRDEGENNDDSQSGSNASSGEYEVLRLVDICYGDPNDEGKSG
LHFKVRWKGYSPSEDTWEPIANLENCGDSIKDFVRRGQQLKILPLPGDVDMICGGPPCQGI
SGYNRHRNTDDPLSDEKNRQIIIFMDVVEFLRPKYVLMENVADILRFDKASLGRYALSRLV
HMRYQARLGTMAAGCYGLPQFRLRVFFWGALPSERLPPFPLPSHDVIVKYWPSPEFERNT
VAYEEGQPRDLEEALVLRDAISDMPAVTWHETREERPYEIPPETEFQKYMRLSKHEIMSCS
STGVKETKEPVLSDHRPCQLNEDDYLRVCLVPRRKGANFRDLPGVIVGGDNVARRDTKD
PKVLPNGKPMVPDCAFNFENGKSKRPFARLWWDETVATLVTFPNHRAQAILHPEQDRVLT
IREYARLQGFPDFYRFTGTLKERYCQVGNAVAVPVGRALGYALGLAYQRLAGNEPLIKLPS
NFSFLTPPIDDIVVLQT

>StDRM5
MRVQKFVLDQCRKWNLVWVGRNKVAPLEPDEFEMLLGFPKNHTRGGGISRTDRYKSLG
NSFQVDTVAYHLSVLKDMFPNGMNVLSLFSGIGGAEVALYRLGIQLNNVVSVEKSEVNRN
IVRSWWEQTNQRGNLIDFDDVQQLNGDRLEQLIDSFGGFDLLIGGSPCNNLAGSNRVSRD
GLEGKESSLFYDYVRILDLVKSIMSRQR

>StDRM6
MDKNLSGEDNDDIDWDTEDELEIQEIQDTVFPSCTDLRTTGQHVVCCDGEVSSSSVPFRS
KFIQQFVVMGFPEESIAKAIEQNGENSDLVLDSLLTFKALDDSPEEQPSVSPPLEPSISSDES
ASEYNENVLDNVYEEDSWSSDSDNYINTVKQCYLNDEGSSLSEKEKMLLFLANMGYPAE
EASIAMDKCGPKASLPELVDFICAAQMSRAEDPYLLEDVKPNLKDILNDCGGYKKRKMY
NELCNRKKQREISGEEPIRLPKPMIGFGIPTESVPRMVQRILPKKTIGPPYFYYENVALAPKG
VWDTIKRHLYEIEPEFVDSKYFSATARKRGYLHNLPIENRFPLFPLPPRTIHEALPLSKRWW
PSWDSRSKLNCLQTAIGSARLTDKIRKAVEKYDGEPPMEIQKYVLYHCKKWNLVWVGRN
KVAPLEPDEVEMLLGFPKNHTRGGGISRTDRYKSLGNSFQVDTVAYHLSVLRDLFPNGINV
LSLFSGIGGAEVALYRLGVPLNNVVSVEKSEVNRNIVRSWWEQTNQRGNLIHFDDVQLLS
RDRLKKLIESLGGFDLVIGGSPCNNLAGSNRVSRDGLEGKESSLFFDYVRILDDVKSIMSR
HR

>StDRM7
MDNNFSGEDNDNIDWDTEDELEIQEIPDATFSSCTNLRNVGHHTVSGHGEASSSSVPCQSK
FIQQFIVMGFPEESIAKAIEQNGENEGLVLDALLTFKALEDSPEEQPSTSAQMEPSISSDDSS
SQCNENVLDDVYEDDSWSSDSDNCMNSAKQTYLNDDNSSLSENENSLLFLANMGYPAEE
ASIAMERCGPEASFSELIDFMCAAQMAREEDVYLPEDEKPKLNSGGYKRKMYNELPVRK
KQRAITDEEPIHLPRPMIGFGVPTESLPAIVQRTLPEQAIGPPFFYYENVALAPKGVWDTMS
RFLYDVEPEFVDSKYFCATARKRGYIHNLPIEDRFPLLPLPPRTIHEAFPLTKKWWPSWDTR
TKLNCLQTSIGSARLADRIRKAMKAMENFDGEPPLMVQKYVLDECRKWNLLWVGRNKA
SPLETDEFEMLMGFPRNHTRGGGISRTDRYKSLGNAFQVDTVAYHLSVLKDLYPNGINVL
SLFSGIGGAEVALYRLGIPLNNVVSVEISEVNRNILRSWWEQTNQKGNLIDFNDIQQLNGD
VLEQMIDSFGGFDLVIGGSPCNNLTGSNRVTRDGLEGKDSSLFYDYVRIVDLVKSIMSNRR
V

>SmMET1
MFAPMGHGLSFIEVKLPPAFTLPHLPTHTQTPENAHSSATSTQPPPLLHRRDRSSSFAFASFR

KRKQISSASTKDMVTNQKEKKKRASQTVEEPTVSRKMPKRAAACSDFKEKSVRISDKDS
VIETKKDGVVEEEVLAVRMTAGKEDGRPCRRLIDFTFHNSDGISQPFEMLEVDDIFISGLIL
PLEESADKEKCKGIRCEAFGRIEEWAISGYEDGSPVIWVSTEVADYDCIKPLGSYKKFYDR
FYAKASACVEVYKILSKSSGGNVSLDELLAGVVRSMSGMKCFSRGVSIRDFILSQVLTALK
DESSKLVDFAEAQPVSLPGNLRIGPKIEDENKNDLLAKPCLTEEDEDSKMARLLQEEEDW
RSMKQKKGRGSSSSSKYYIMINEDEIANDYPLPAYYKMSNEETDEFIIFDSGIDVLHIEDLP
RSMLHDWALYNSDARLVPLELLPLRPCDEIDVTIFGSGIMTADDGSGYICDGDSTQSSNGS
GASAVEGIPVFLSAIKEWVIEFGSSMISISIRTDMAWYRLGKPSKQYAPWFERVLKTARLAI
SIITMLKEQIRVSRLSFADVIKRISELAKDHPAFISSTLQVVERYVVVHGQIILQQFSEYPDDII
KKCAFVIGLAKKMEERHHTKWLVKKKKLLHRNEQNLNPRAAIEPVVSKRNAMQATTTR
LINRIWAGYYSNYSPEARDGVNGEVKEVHEIEEQEENEEDDALEEKLVVFEQTQTTKSAP
RQTKLSSCVEEVKWGGESVGKLLNGEDLYKRAIVHGNEIVVRGAVLLKDDEMDDFPPIYF
VEYMFEKSNGTKMFHGRLMQRGCQTVLGNTANEREVFLTNECMDFQLQEVKESIFVDLR
SIPWGHQHRKENANAEKIDRARAEERKKQGLPTEYYCKSLYWPERGAFFTLPYSSLGLGS
GSCEACNIKESERQKEKFILDASLTSFIYQGTKYSINDYIYVSPSYFSSEEMEAEIYKAGRNV
GLKAYVICQLLEICDLKKSKRSEANSVQVKVRRFFRPEDISSEKAYSSDIREVYYSEETHTIP
LDVIEGKCEIRRKKDLGPQDIPFTSHHVFFCEHLYDPSRGSIKQLPSHIKLQYSTGQLNDDA
TFRKKKGKCKEGENDDKEPEKVVSDEHRLTTLDIFAGCGTSLTKWAIEYDAAAGDAFKLN
HPGSLVFVNNCNVILRAIMQKCGDSDDCISTPEAAELAASMDQVEVENLPLPGQVDFING
GPPCQGFSGMNRFNQSTWSKVQCEMILSFLSFADYYRPKYFLLENVRTFVSFNQGQTFRL
TLASLLEMGYQVRFGILEAGAYGVPQSRKRAFIWAAAPEELLPDWPEPIHVFSAPELKISLS
KNLQYSAVRSTSKGAPFRPLTVKDTIGDLPPVVNGASNTSLEYQSDPISWFQKKIRGNLNIL
TDHISKEMNELNLIRCQKIPKRPGADWRDLPEEKVKLSTGQMADLIPWCLPNTAKRHNQ
WKGLFGRLDWEGNFPTSITDPQPMGKVGMCFHPDQDRIVTVRECARSQSANSVSLTTYSL
VRSGLFFYTRRSSETELEHNTEDVASLPSTPNKLGFPDSYKFFGTIHHKHRQIGNAVPPPLA
YALGSKLKEAVEKKRTRNASPTSTATGATPFIRFTFAGEIWQIPYDLGKFTVSFIRFIRHFHPS
RSGAAPCFFDEKRLFDINRDRGNVVQS

>SmCMT1
MGRGKKRAEATSSPPPPISASPKKPKRSTPVDTELGFISSPVPSHEAKSRWPHRYQTKNVK
TTVTVTLSDGSTEEKELFQARSHYTRAVVDAVVFELFDDAYIQASEGELDFIAKIVEMFET
TNGEMYICTQWFYRAKDTVIKNQDYLIDAKRVFYSDIRDDNPLSCISRKAKIKQLPPNMA
AAAREKAMDSYDLYVDAMYTFPHAFSNLSKDINNSPLTNGVMETKTEDVKKSPEDSQRS
EITVLDLYCGCGAMSTGLGIGASLLGEKIVTKWAVDFNSYACESLKYNHPNTEVRNEDAE
NFFDLLKEWENLCTKFNVVGSKDIELEPQDSDSEASEVDDGVVIPSEGEYEVQKLLAICY
GDPNKSKKTGVYFKVRWKGYGRADDTWEPIDGLSNCEERIKEFVLRGYRARVLPLPGDV
DFICGGPPCQGISGFNRFRDALAPLKDKKNHQLVVYMNIVEFLKPRYILMENVTDILKFAK
AQLTSYAVGRLVSMNYQARLGIMAAGAYGVPQCRMRFFLWGAGPTEILPQFPLPTHEVLK
KGVVINEYKELIIGHGEEHCQLHKAALLGDAISDMPEVTNNTDQDEMPYGNAPRTEFQKL
IRLSRQKIFGYSDAKKKNSHKSILHDHRPYQLNQDDYERVCHVPKFKGANFRNLPGVLVG
PDRVVKLDESIDRPRCKSGKPLVPDYALSFEDGKSCKPFGRLGMDDIVPTVVTRPEPHNQV
LLHPNQDRVLSVRENARLQGFPDCYKLFGPVRERYMQIGNAVSFAVSIPLGYCLAKALQG
AQLTTPLSLPFKFPDCLGQLQSLRQEPEELVSE

>SmCMT2A
MKEQQKLPDQLPLRKSSSISSSPSARLCNGAKLEPEARPVDALALSLYVAEAEQQQQPLSI
CYPSHEEEEEDNSVRRSPRFTSNKYKYLSPERSRSQKHTALVVSKLSMSPRIFFSSSAPALTL
TDGGNVEAGPKKRRRLLALPGPAGSGVCLSERCLRSRTVLMRVAESPEASGAKRLRSVGG
SPQIEMLALPQATPISSKNRTRPSASLNKECSVTPEKESSLAGVRRNGESAVKSKKKEKSIL
CCFIGEPIPEEEARERWQWRYDLKSRIKGKNWKINAEEDEIILNVASHYAQAKVGNFVLNI
GDCAFVKGEGNKKHIGRILEFFKTTEGEDYFRVQWFFRAEDTVLKDTASSHDKRRIFYSIL
MNDNVLDCIISKVKIVNVPPMPCLKRNRVQSAAFYYDMEYSVQYSTFRTMVTEKSMGNE
SFLSNSKHPDDGPITLTPLELLPGYDSPKQELTLLDLYSGCGGMSTGLCLGAKLSSVNLMT
KWAVDYNSSACESLRLNHPETNVRNESAEDFLDLLKQWESLCKRYVSDIERTLKCELEEL
PEAEAEDCLIPSDTEESSGEYERIEDFVRRGFNAKILPLPGDVGVICGGPPCQGISGYNRHR
NFASPLEDERNHQIVVFMDTVEFLKPKYCLMENVVDIIRFDKGSLGRYALSRLVLMRYQA
RLGTVASGCYGLPQFRLRVFIWGAHPGETLPQFPLPTHDVVARYWPPIEFERNIVAYDEGQ
PRALHGAIVLGDAISDLPPVTNDEDREEMAYGKPPETEFQRRIRTGAGDVLYDHRPLKLSE
HDYMRVCRVPHRKGANFRDLEGVVIGEDNVARRDPTREAVMLPTGRPLIPECVFKLELGK
SRRPYGRVWWDETVSTVVTFPHIRAMAILHPEQDRRLSIRECARLQGFPDYYRFCGTIKER
YSQVGNAVSIPVARALGYALGIAYRKLAGEDPLMTLPPDFSSST

>SmCMT2B
MADSAQLDSADPPLAPPSPPPSLPLQLNGNSDPATPLPSSTDGGSDILDNYPQSNGVAMKK
SANRRVETLRTSEDHFRRRSPRLTGNVAGERSVREVSSLKLDGPDEIYPPLKKPKGGKQVS
FFIGGPVPDDEARRRWTWRYEGAEGKQNNELSPKHKVVVDDDDDGDKLVSNVKCHYSQ
AEISKIVFDLGDCAYVKGPKGRPNYVGKILEFFETMDGENYFSVQWFFRAEDTVIKNDGR
SHDKKRLFYSTLVNDNLLDCIVSKVKVVQIKPNVCLKFVTLKDNTIPPCDYYFDMKYDVD
YSTFSSILTDGDSELFPHPLMMSSKGKYATSKISTKCSLDENCKSELALLDMYSGCGGMST
GLCIGAKACGLDLVARWAVDIDEAACESLKLNHPETQIRNEAAEDFLDLLKEWDCMCRK
YGKYDQKELRLKTVREADEVKLSKKNQESSCEYEVARLIDICYGDPSNSGKRGLKFKVR
WVGYGPSDDTWEPIENLGKCPEHIRDFVQKGIKAQILPRPGDVDVICGGPPCQGISGYNRF
RNFDSPLDDERNRQILIFMDIIEFLKPKFVLMENVIDILRFANGCLARYAISRLVSMHYQAR
VGIMAAGCYGLPQFRLRVFLWGAQPLEASILNPYLGTSDIPLRRNLVLPQFPLPSHDVVIQ
YGFPSDFERNVVAYDEGKFRDLEKKVLLSDALSDLPPVANNEKRDSIPLKTVPEAEFQKYI
RAAKCDMTASPSSVTTTHKKPVLYDHRPYPMNEDDYLRVCKIPKRKGANFRDLPGIVIGA
GNVIRRAKEQDLMPSGKPWVPDYCLSYRDGRSHKPFGRLWWDETVPTVFCFPDHHSRAI
LHPEQDRILTLRECARLQGFPDYYMFSGSLKQRQVFMSYSQVGNAVAVSVGRALGYSLG
MAVQRLSGDEHLLTLPPKFSHSTTVELLSLLNQ

>SmCMT3
MANKRKSESATKDPAAATAEGSRRSKKPASAESTGAVDEETRIPTVIDDGSEDAGEALFLG
EPVAGEEARKRWPHRYQKDNQSTEIVQAKCHFLQAKVDGLVYCLEDDAHVKAEEGKES
YICKIVELFQAVDGTQNFSAQWYYRADDTVIKTCSNLIDNRQVFFSEVKDDNPLDCLVKK
LRIVLLPLKVEDQVKEKLRSTHDYYYDKMYLLPYTSFVNLSSESTTNSNESGSTISSDADG
NSVTHVFSELEGPSGEKSLLDMYSGCGAMSTGLCLGANSNGVKLVTKWAVDLNPYACES
LKLNHPETQVRNEKADDFFHLLVEWEKLCTSYLSSENKGTSSEHVKEEVEDDSEEDENGD
EDDSEVFEVEEILEVCYGDPNEKESPELHFKMGSNDKEPCIHCSSSCPQKLKEFVLRGYKS

RILPLPGAVDVVCGGPPCQGISGFNRFRNKEAPLDDPKNKQLVVFMDIVSHLRPKFVLME
NVVDLVKFAGGFLGRYAMGSLVGMGYQARMGMMAAGAYGLPQFRMRVFVWGALSTE
KLPSYPLPTHNVVVRGVTPLEFEANTVAYEEGCKLDLKKALVLGDAISDLPHVENDEGRD
EIPYGSEPQTEFQRFIRQRRDEMPGYLNVKSEVFEHLLCDHRPLQLNQDDYERVCQIPKKK
GANFRDLPGVRVRPDNKVEWDPDVPRQKVSSGKPLVPDYAMTFVNGSSSKPFGRLWWD
ETVPTVVTRAEPHNQAILHPSQDRVLSIRENARLQGFPDYYKLVGPIKERYIQVGNAVAVP
VARALGFSLARSLRGLSHNEPVFTLPEDYPMMRDVASPTIDMI

>SmDRM1
MILMFLVPIQDENVSGGDVSDVDWTTDDESEINLNPVTTIVGNEEACSSRSASRSKMFDHF
VGMGFSDKLVSKAIEENGEEDSDSILNWLLTFSSLEDSPPQQLSTNFDPCSSDYNEIFPNDL
SDMDSWSDDEIEDCLKPNGAPNGAANGVYRSEKHKMLLSLAASGSLTEREEKLLSLADM
GYPLEEAEIALERCGPKALIGELSDFLCAAQMAREEDCYLPDDDLKPKHLLSGSSKSKKR
KFLEMKKNVIEESIRLPNPMIGYGVPSMQLERVHRSLRDDARAPPYFYYENVALTPKGVW
DTISRFLYDIEPEFVDSKFFSACARKRGYVHNLPIENRFPLIPLPPLTIQQAFPLSKRWWPRW
DPRDKLNCIQTAIASAKLTERIRSALERVESEADPPESVQRYVMEQCRKWNLVWVGRNKV
APLEPDEVEMLLGFPKNHTRGGGISRTDRYKSLGNSFQVDTVAYHLSVLKDIYPNGVNVL
SLFSGIGGAEVALHRLGIKLKNVVSIEKSKVNRDIVRSWWEQTNQTGMLIDFDDVQQFDD
TRIEQIIGSIGGFDLVVGGSPCNNLAGSNRVSRDGLEGKESSLFYDYFRILGSVKSVMRNNR
FA

>SmDRM2
MCDVVDISDDEDSFLLENDAGVIPKDENLDYDLPPPKTFSMPREEDGASSSGTNLRSSFIG
MGFAPALVDKAIKEKGEENSELILEALFSYDALQKPKNEVLDNSFSGECSDSLAGNLRAEE
DFHKSESSDSLGRLFAEGTKKNAYVEADIPLKVELDVGDSVIDEKTASLLKMNFSLDEVGF
AMQRLVANLQDCNNEVLFGIMEKSLQLLEMGFTENEISAAFERCGSEAPVTLLAESIVTGG
TCPLPDKYSSTLLSTSGVRRSFQSSKRKMEDYNSICIKTEEYSSDVASQVRTSDLLEKLKGK
MPEVDIDEPNNFKKPKEEFMEDYPSVGNSAAVLQRRGGCFASIDDDRKPTISLPNACRSLS
DGVAKPPYFLYGNVTSLSPGSWAKICKFLYSVQPEFVDSHLFSALNRKEGYVHNLPSEDRF
HIFPKGPMTIAEAIPYAKKCWPSWDNRKQLSSITCDTTGLPYLCEKLGRMLAECNGPPSAE
LQTRILQQCVAKNLVWVGKQKLGPLEPEHLEHIMGYPQHHSRIAGYSLTERLKSLKLSFQT
DTLGYHLSVLKRLFPSGVTVLSFFSGIGGAEVALHRLGIRLKGVVSVEPCETKRRIMKKW
WEKSAQSGELIQLESINKLT

>SbCMT3a
MAPSSPSPAAPTRVSGRKRAAKAEEIQEEEVVVASSAKRSRKAPSSGKKPKPTPKQAKPAK
AARKKKGEAERKEPVEDDVCAEEPDEEELAMGEEEAEAAADEQAMQEEVAAVVAGSPG
KKRVGRRSAAAGGDHEPEFVGNAVPAAEARSNWPKRYERSTAAKKPEEDEELNAKCHYR
SAKVDNVVYCLGDDVYVKAGENEADYIGRITEFFEGTDRCHYFTCRWFFRAEDTVINSLV
SINVDGHKHDPRRVFLSEEKNDNVLDCIISKVKIVHVDPNMDPKAKAQLIEHCDLYYDMS
YSVAYSTFANISSENGQSGSETASGISSDDVDLEAPSSMPERTATLLDLYSGCGGMSTGLCL
GAALSGLKLETRWAVDLNSFACQSLKYNHPQTEVRNEKADEFLALLKEWAVLCEKYVHQ
DVDSNLAGSEDQEDEGSPLDKDEFVVEKLIGICYGGSGRKDGIYFKVQWEGYGPEEDTW
EPIDNLSDCPLKIREFVQEGHRRRILPLPGDVDVICGGPPCQGISGFNRFRNRDEPLKDEKN

KQMVTFMDIVAYLKPKYVLMENVVDILKFADGYLGKYALSCLVAMKYQARLGMMVAGC
YGLPQFRMRVFLWGALSSMVLPKYPLPTYDVVVRGNTPNAFSQCMVAYDEKQKPHLKK
ALLLGDAISDLPKVENHQPKEVMEYGGSPKTEFQRYIRLSRKDMLDWSFGEEAGPDKGK
LLDHQPLRLNNDDYERVQQIPVKKGANFRDLKGVKVGANNIVEWDPEIERVKLSSGKPLV
PDYAMSFIKGKSLKPFGRLWWDETVPTVVTRAEPHNQIILHPTQARVLTIRENARLQGFPD
YYRLFGPIKEKYIQVGNAVAVPVARALGYCLGQAYLGESEGSHPLYQLPPSFTALGRTAVQ
ARASPVGTPVGEVVEQ
>SbDNMT2
MEAPAPWRVLEFYSGIGGMRYSLMASGVRAEVVEAFDINDVANDVYEHNFGHRPCQGNI
QTLTASDLDKYKAHAWLLSPPCQ
PYTRQGLQKHSADARAFSFIKILNLMQDMSYPPQMLFVENVVGFEVSDTHDQLLEVLSSL
NFNTQEFILSPLQFGVPYSRPRYFCLAKREPMRFRNASVN
NKLLQTPMCLSMTLNSTSQGSDHQTEEELEPVCKPIKDFLVKEADGGTRNESVLQAYMVP
VNLIERWGNAMDIVYPESKRCCCFTKSYYRYVKGTGSLLA
TSENIKWVPEEKLQVSSLKELGLRYFTPREVANFHSFPSSFYFPDHISLKQQYAMLGNSLSI
AVVGPLLCYLFAEA*
>SbCMT3b
MAPSSPSSAEPTRVSGRKRAAKAVEIHQSQEEEEEQKVVAASSAKRSRKAASSGKKPKPTP
KQAKAGRKKKGEAERKEPVEDDVCAEEPDEEELAMGEAEAEAEAEADEQAMQEEVAAV
AAGSPGKKRVGRRSAAAGGDHEPEFVGNAVPAVEARSNWPKRYERSTAATKPEEDEELK
ARCHYRSAKVDNVVYCLGDDVYVKAGENEADYIGRITEFFEGTDRCHYFTCHWFFRAE
DTVINSLVSINVDGHMHDPRRVFLSEEKNDNVLDCIISKVKIVRVDPNIDPKAKAQLIEHCD
LYYDMSYSVAYSTFANISSENGQSGSETASGISDGDVDLETSSSMPERTATLLDLYSGCGGM
STGLCLGAALSGLKLETRWAVDLNSFACQSLKYNHPQTEVRNEKADEFLALLKEWAVLCE
KYVQDIDSNLAGSEDQEDEGSPLDKDEFVVEKLIGICYGGSGRKNGIYFKVQWEGYGPEE
DTWEPIDNLSDCPLKIREFVQEGHRRKILPLPGDVDVICGGPPCQGISGFNRFRNRDEPLKD
EKNKQMVTFMDIVAYLKPKYVLMENVVDILKFADGYLGKYALSCLVAMKYQARLGMM
VAGCYGLPQFRMRVFLWGALSSMVLPKYPLPTYDVVVRGGAPNAFSQCIVAYDETEKPSL
KKALLLGDAISDLPKVENHQPNDVMEYGGSPKTEFQRYIRLGRKDMLDWSFGEEAGPDE
GKLLDHQPLRLNNDDYERVQQIPVKKGANFRDLKGVKVGANNIVEWDPEIERVKLSSGK
PLVPDYAMSFIKGKSPKPFGRLWWDETVPTVVTRAEPHNQAILHPTQARVLTVRENARLQ
GFPDYYRLFGPIKEKYIQVGNAVAVPVARALGYCLGQAYLGESEGSHPLYQLPPSFTALGQ
TAVQARASSVGTPAGEVVEQ

>SbCMT1
MPIHSGPDSSSPVPSPSCSPIPVALEERAPVVIPGSEPPLPLPVASSEAILEEGTLECTFNSVPS
PPALPSSDRPDLGAEDGPNDWSSLLKVLSGSMPVIDMAATERPGDNAANCSMCMSDIATG
DGTPLRSSPRIMAVKTKANAASLEHEIESSRASEHRLAVSRQISTSEGSQQPVSARLFEETT
VASGKPVHLPDGVKPQGSPDIIDIYTSSTDVVVALPVVSKRSLKNKVVSIKTKANAASLEQ
EIESSRASKHRLAVSQQSTVSEGSQQPVSARLFEETTVSSGKPVHLPDGVKPQGSPEIVAIH
CSSTDVVVALPVVSKRSLKNKVVSSSPRKTRSSSKVVVNSNRVSAVSQVMNCVPAVHKAS
SSIPPRKHKLASEKCLPNLERVDAMLHNSGLISANKAVHGTPLEVEAALSQPPKAKQPRIC
LGKCSLNLKSAENSSSSICELPMVTVTSAPEDKPISAPENKPLDTHCTDSEMVDSMDGSYF
FVGDAVPDEEAQKRWPHRYQNNHCLLKKDKRSNTQTVSNVGKAVLDVKCHYLEASICG

STLCIGDCAFIKGLEGKPHYIGRLLEFFETTADEYYFTVQWFFRAEDTVMEDQAQSHDPRR
LFYSDLKDDNLLDCIVSKVTIMPVSPSLNEKPRLIPSFHYYYDMKYSLDYSTFSTMEMGDP
NDTLHSHYTSSNNVKRIDFTEKQKSPTSEMRELSLLDLYCGCGGMSTGLCLGARGGGVN
LVARWAVDGDEVACETFRLNHPETRVRNETTDDFLELLKEWKKLCKTYVKPCSKVKSQS
NVPTQSSNGTPDCSTFSTEEFEVWKLVDICFGDPNGVRKRALYFKVRWKGYGPNDDTWE
PMEGLKNCKDAIRDFVIEGHNKKILPLPGDVDVICGGPPCQGISGYNRNREFDAPFNCERN
KQIIVFMDVMQFLKPKYIYMENVLDILKFADATLARYALSRLVSMHYQAKLGVMAAGCY
GLPQFRMRVFLVGCHPKEKLPPFPLPTHEAIVKNGCPLAFERNLVGWPDSMPMQLEKPIVL
EDALSDLPEVENGEKREEMLYMKGPQTEFQRYIRSFNSEVLGSRAHVIKVSKSKLFDHRP
RALDVDNYLRVLQIPKKKGANFRDLPGVIVGPDNIARLDPTKERILLPSGNPLVVDCVLAY
KNGKSLRPYGRLWWDEVVGTVLTCPNAHMQALIHPAQDRLLTIRESARLQGFPDSFRFRG
TVKDRYRQIGNAVAVPVGRALGYALAMAYLNKTGDDPLMVLPPKFAFSHDVQGLSSVNG
LHGRPIG

>SbMET1
MTHELSGDMVKTPRSPVTTGTRRCRAKPQKKEKESTENSKLENGSQDATEEVHHGVEKG
DGHVTRKRPRRAAACSDFKEKSIRLSEKKSVVMVKKNRMEEEEIDAVSLTKLGPEDPPPC
RKLIDFILHDAEGNPQPFEMSEIDDFFITALIMPMDDDLEKERERGVRCEGFGRIEDWNISG
YDEGTPVVWVSTDVADYECVKPATNYKSYFDHFYEKAQVCVEVFKKLAKSVGGNPNQG
LDELLASVVRSINAMKGYSGNMSKDLVISIGEFVYNQLVGLDETSSNDDEKFATLPVLLSL
RDQCRSRVELTKLPSNISNTSLKIKDTVCEGTAEEEDDDAKLARLLQQEEEWKMMKKQR
GRRGTPSQKNVYIKISEAEIANDYPLPAYYKPFSQEMDEYIFDSDDSIFSDDVPVRILNNWT
LYNADSRLISLELIPMKSGAENDVVVFGSGFMRDDDGSCCSTAESVKSSSSSSKADQQDA
GVPIYLSPIKEWLIEFGGSMICITIRTDVAWYKLRQPTKQYAPWCEPVLKTARLAVSIITLLK
EQSRASKLSFADVIKKVAEFDKGNPAFISSNIALVERYIVVHGQIILQQFADFPDETIRRSAFV
SGLLLKMEQRRHTKLVMKKKTQVMRGENLNPSAAMGPASRRKAMRATTTRLINRIWSD
YYAHHFPEDSKEGDGNEAKEVDDEQEENEDEDAEDEGQIEEDKISKTPPSTRSRKLLSQTC
KEIRWEGETSGKTSSGETLYKCAYVRELRIPVGGTVVLEDDSGDTVMCFVEYMFQKSGG
AKMVHGRILQKGSHTILDNAANEREVFLTNDCLEFKLDDIKELVSVDIQSRPWGHKYRKE
NSETDKVEQAKAEERKKKGLPMVYFCKSLYWPEKGAFFALPRDKMGLGSGVCSSCEHIE
PDSDELKVLSKTSFVYRKVTYNVNDFLYIRPDFFSEDEDRATFKAGRNVGLKPYAVCQILA
IPEGAGSKKLNPASANISARRFYRPDDISTAKAYASDIREVYYSEDVIDVPVDMIEGKCEVR
KKNDLASSDLPVMFEHVFFCELIYDRASGALKQLPPNVRFMSMVQKATIASKKNKGKQIC
ETDQIDSGKWLDVPKENRLATLDIFAGCGGLSEGLQQAGVSFTKWAIEYEEPAGEAFSKN
HPEAVVFVDNCNVILKAIMDKCGDTDDCISTSEAAEQAAKLPELNINNLPVPGEVEFINGG
PPCQGFSGMNRFNQSPWSKVQCEMILAFLSFAEYFRPRFFLLENVRNFVSFNKGQTFRLAV
ASLLEMGYQVRFGILEAGAFGVAQSRKRAFIWAAAPGEMLPDWPEPMHVFASPELKITLP
DGQYYAAARSTAGGAPFRAITVRDTIGDLPKVENGASKLTLEYGGEPVSWFQKKIRGNM
MVLNDHISKEMNELNLIRCQHIPKRPGCDWHDLPDEKVKLSNGQMADLIPWCLPNTAKR
HNQWKGLYGRLDWEGNFPTSVTDPQPMGKVGMCFHPDQDRIITVRECARSQGFPDSYEF
AGNIQNKHRQIGNAVPPPLAYALGRKLKEAVDNKRQEAGVGVPAP

>SbMET2
MTHELSGDMVKTPRSPVTTGTRRCRAKPQKKEKESTENSKLENGSQDATEEVHHGVEKG

DGHVTRKRPRRAAACSDFKEKSIRLSEKKSVVMVKKNRMEEEEIDAVSLTKLGPEDPPPC
RKLIDFILHDAEGNPQPFEMSEIDDFFITALIMPMDDDLEKERERGVRCEGFGRIEDWNISG
YDEGTPVVWVSTDVADYECVKPATNYKSYFDHFYEKAQVCVEVFKKLAKSVGGNPNQG
LDELLASVVRSINAMKGYSGNMSKDLVISIGEFVYNQLVGLDETSSNDDEKFATLPVLLSL
RDQCRSRVELTKLPSNISNTSLKIKDTVCEGTAEEEDDDAKLARLLQQEEEWKMMKKQR
GRRGTPSQKNVYIKISEAEIANDYPLPAYYKPFSQEMDEYIFDSDDSIFSDDVPVRILNNWT
LYNADSRLISLELIPMKSGAENDVVVFGSGFMRDDDGSCCSTAESVKSSSSSSKADQQDA
GVPIYLSPIKEWLIEFGGSMICITIRTDVAWYKLRQPTKQYAPWCEPVLKTARLAVSIITLLK
EQSRASKLSFADVIKKVAEFDKGNPAFISSNIALVERYIVVHGQIILQQFADFPDETIRRSAFV
SGLLLKMEQRRHTKLVMKKKTQVMRGENLNPSAAMGPASRRKAMRATTTRLINRIWSD
YYAHHFPEDSKEGDGNEAKEVDDEQEENEDEDAEDEGQIEEDKISKTPPSTRSRKLLSQTC
KEIRWEGETSGKTSSGETLYKCAYVRELRIPVGGTVVLEDDSGDTVMCFVEYMFQKSGG
AKMVHGRILQKGSHTILDNAANEREVFLTNDCLEFKLDDIKELVSVDIQSRPWGHKYRKE
NSETDKVEQAKAEERKKKGLPMVYFCKSLYWPEKGAFFALPRDKMGLGSGVCSSCEHIE
PDSDELKVLSKTSFVYRKVTYNVNDFLYIRPDFFSEDEDRATFKAGRNVGLKPYAVCQILA
IPEGAGSKKLNPASANISARRFYRPDDISTAKAYASDIREVYYSEDVIDVPVDMIEGKCEVR
KKNDLASSDLPVMFEHVFFCELIYDRASGALKQLPPNVRFMSMVQKATIASKKNKGKQIC
ETDQIDSGKWLDVPKENRLATLDIFAGCGGLSEGLQQAGVSFTKWAIEYEEPAGEAFSKN
HPEAVVFVDNCNVILKAIMDKCGDTDDCISTSEAAEQAAKLPELNINNLPVPGEVEFINGG
PPCQGFSGMNRFNQSPWSKVQCEMILAFLSFAEYFRPRFFLLENVRNFVSFNKGQTFRLAV
ASLLEMGYQVRFGILEAGAFGVAQSRKRAFIWAAAPGEMLPDWPEPMHVFASPELKITLP
DGQYYAAARSTAGGAPFRAITVRDTIGDLPKVENGASKLTLEYGGEPVSWFQKKIRGNM
MVLNDHISKEMNELNLIRCQHIPKRPGCDWHDLPDEKVKLSNGQMADLIPWCLPNTAKR
HNQWKGLYGRLDWEGNFPTSVTDPQPMGKVGMCFHPDQDRIITVRECARSQGFPDSYEF
AGNIQNKHRQIGNAVPPPLAYALGRKLKEAVDNKRQEAGVGVPAP

>SbDRM3
MQVKIEDHAEDGGIDADVRGLGEAAVDPLPGSVHATFKEEEGEPSSSSGKLRSEFIGMGFS
SKLVDKVLQRHGDDDSNTILESLLSYSDLKQSGSESSGSLGSLFDSDNEENNSSLESRKVIN
QDIKPELDSFSEKWSFLLRTMNFSQQEVDLAFKQLGDEAPLEQLVDFIVNAQSGVSSGGPE
NGDATNEGKTELLYGVMEKTLSLLQMGFTEEEVSSVLENSDQRATIQELADSILARRIANS
IEQKEVKIESDFLGEAEPDYSSYQPSYSAASYYDDDNNNIRVKRAKHVFIDDTGASSSHLG
NPWSMGTSDMPVKVELEAMTPGRRANVQGDLAKPPFFLYGNMVDIPKDTWYQLTQFLY
NVEPEFVNSQSFSALTRKEGYIHNLPVEKRRVVVPKSPMTIEEALPFTRQWWPSWDTRKHI
SVVTIEVGEIEQTCERLGSMVRESRGVLSEARQMQIIHQCRVSNLIWVGQNKLGPLEPRQV
ERILGYPHNHTNLFELNQSDRFGAMKFAFQTDTLSYFLSVLKGMYPDGIRVLSIYSGIGGA
EVTLHRLGIPLKCVISVEESEVNRKILRRWWSKTEQTGVLRQHPGIWKLKTHVIEDLVKEF
GGFDLIIGGNYTSCKGGNTVNTTMGLDSNRFYEYARVVKRVRTAVGLS

>SbDRM1
MADRVGGSDDGDKSEWNSDGEVARSLDGAGSPIAPSRNKLKDAPGPSLLVNGAGPSASL
VEDFVGMGLIKGNGDGGAESLLELLLAYKAIGNDPSLDKSSASVCGPRTICDDKDILANW
DAHDASTSSDRDHASDDSGDEDFLQGLSQKDEKIESLVRMGFPKDEAEMAIVRCGQDAP
MSVLIDLIYSSEASEDGYYGNFSGHEDDSFGGRKEKRKRSEDAEQGSSHDEPMPLPNPMV

GFNLPNVSLRSVDRSLPSKAIGPPFFYYENVAIAPKGVWTTISQFLYDIQPEFVDSRFLCAA
ARKRGYVHNLPIENRSPLLPLPPKTIFEAFPHTKKWWPSWDPRKQFNCLLTSMAKPKLTE
QIHHALAKCKDPPPRRVQKYVLETCRTANLVWVGLNKVAHLEPDEMEFLLGFPKDHTRGI
GRPERFKSLGNSFHVDTVAYHLSALRDMFPHGMNVLSLFSGIGGAEVALHRLDIRMKTVV
SVEISETNRFVLRTWWNQTQTGTLIEITDVQSLTTERLESCIRRIGGFDLVIGGSPCNNLAGR
NRFHRDGLEGEQSSLFYHYYRILDTVKSIMGRM

>SbDRM2A
MAHWVSDGDGSDSFEWDSDGNGEEAGSFNTAGASSSAMASTNTDAPGPSRRVSQVANG
NGKAGPSASLVQRYIDMGFAEEIVVKAIKDNGDNGADALVELLLTYQELGNDLNVDNGF
ASGCVPQTVDDSGDDDFLENWDDLDAGGRSTRVANSVDDSGDEDFLHEMSQKDNKIDS
LVKMGFPEDEAALAITRCGQDASISVLADSIYASQTAGDGYCGNLSDYEDGGRNKGRFM
DGNNKKRKRYGSQALGSRGPLDGSADEPMLLPNPMVGFSLPDQWPRPVNRDLPALAMG
PPYFYYENVALAPKGVWTIISRFLYDIQPEFVDSKYFCAAARKRGYIHNLPLENRSPLLPKP
PRTITVAFPHTKRWWPSWDPRQQFNCLQTCVSSAKLLEKIRVTLTNSSDPPPPRVQKLVLEE
CRKWNLAWVGLNKVAPLEPDEMEFLLGFPKDHTRGICRTERFRSLGNSFQVDTVAYHLSV
LKDMYPEGMNVLSLFSGIGGAEVALHRLGIRMKTVISVEKSEVNRTILRSWWDQTQTGTL
IEINDVQTLTSERIEAYIRRFGGFDLVIGGSPCNNLAGSNRHHRNGLEGEHSSLFFQYVRILE
SVKSIQRL

>SbDRM2b
MAHCVGDSDGSDNFEWDSDGDGEEAASFNAAGASSSAMASTNADAPGPSTRVANGNGK
AGPSASLVHKYMDMGFPEEIVLKAMKDNGDNGADSLVELLLTYQELGNDLKVDNGFAS
GCVPQNVDDSDDDDILENWDDEDAGGRSTGVANSVDDSGDEDFLHEMSQTDEKVYSLV
KMGFPEDEAALAVTRCGQDASISVLVDSIYASQTAGDVYCGNLSDYEDNSYGGRNKGRF
MDANKKRRKRYEGQAHGSRGPLDGSSDEPMPLPHPMVGFSLPHQCTRPVDRSLPSQAM
GPPYFYYENVALAPKGVWTTISRFLYDIQPEFVDSKYFCAAARKRGYIHNLPLENRSPLLPI
PPKKISEAFPHTKRWWPSWDPRRQFNCLQTCVSSAKLLERIRVALTNSSDPPPPRVQKFVL
EECRKWNLAWVGLNKVAPLEPDEMEFLLGFPKDHTRGISRRERYRSLGNSFQVDTVAYHL
SVLKDMYPQGMNVLSLFSGIGGAEVALHRLGIRMKTVISVEKSEVNRTILKSWWDQTQT
GLLIEICDVQTLTSERIEAYVRRIGGFDLVIGGSPCNNLTGSNRYHRDGLEGEHSSLFYHYV
RILDSVKSIMERL

>BdCMT3
MAPSSPSSAAAAAPARASSRKRTASAKAELAQEATKRPRKAAASSGKKKVTKPKAKAEK
APRKKKEVEVLGEDEVCAEEPDEEELALGEEDESAASGEQQPEEPAAARKRVAQPIRARN
QAGGDKDHGFVGAPFPAGEARSKWPQRYQPTKPRRPEEEEEDPKARCHYRSAKVDEAIY
NLGDDVYVMAGENEPHYIGRITEFFEGIDKKCYFTCRWFFRPEDTVISTAKFVNDHTHDP
KRVFLSEEKNDNVLECIVLKVNIVHVDPNMDSEAKAQLVAESDLYYDMSYSVAYSTFANI
TSDTNDNSGISSDVDSEAASPVRTAVLLDLYSGCGGMSTGLCLGSALAGLKLETRWAVDL
NSFACKSLKYNHPGTEVRNEKAEDFLALLKEWAILCDTYVHGNNSDPASPSEDEEEDDEP
LGKDEFVVEKLLEICYGGSGREKAIYFKVQWKGYGPEEDTWEPIGNLSDCPLKIKEFVQE
GHKRNILPLPGDVEVICGGPPCQGISGFNRFRNRKEPLKDEKNQQMVTFMDIVSYLKPKF
VLMENVVDILKFADGYLGRYALSRLVSLNYQARLGIMVAGCYGLPQFRMRVFLWGALPA

MVLPKYPLPTHDVVVRGGAPNAFSQSIVAYDETQKPTLKKALLLGDAISDLPKVDNFQPH
EVMEYGAQPKTEFQSYIRLSRKDMLDYSFGDNTCPEEGKLLDHQPLRLNQDDYDRVQQI
PVKKGANFRDLPGVKVGANNIVEWDPEVQRVYLKSGKPLVPDYAMSFIKGRSLKPFGRL
WWDETVPTVVTRAEPHNQIILHPNQARVLTVRENARLQGFPDYYRMNGPIKEKYIQVGN
AVAVPVARALGYSLGRAYQGEMEGSNPLFTLPDSFTNVGQTVVLARASSVGTPAGEVVEQ

>BdDNMT2
METPPPWRVLEFYSGIGGMRYSLTASGVQAEVVEAFDINDVANDVYEHNFGHRPCQGNIQ
TLTASDLDKYKAHAWLLSPPCQPYTR
QGLQKHSADARAFSFIKILNLMSNMRFPPQVLFVENVVGFEVSDTHDQLLEVLSILNFNTQ
EFILSPLQFGVPYSRPRYFCLAKQESMCFQNASANKKLL
WTPTCLKFNSTTQNSYDQNEDELEIACRSIKDFLETQSINIGDQDCSGTISACNLKEADGCT
PSETVSQDYIVPLNLIERWGNAMDIVYPESKRCCCFTK
SYYRYVKGTGSLLATSKNLKPIPKENLEISSLNELRLRFFTPREVANLHSFPSSFCFPDHISLR
QQYAMLGNSLSVAVVAPLLHYLFSEK*

>BdCMT3b
MAPSSPPSAALRASSRKRTASAKAAPDQEPSAATKRPRKGAASSSAKKKTADPKAKALKA
PRKKKEVAAAEEKLSEDEVCAEEPDEEELELGEEDESAASGEQGQATARRRVAQPTRERN
LAAGDKEPEFVGEPFPADEARSKWSQRYQRAAPRRPDEEPELKARCHYRSANVDGTVYA
LGDDVYVKAAENEADYIGRITEFFEGTDRHCYFACRWFFRPEDTVISTAKFVDDHTHDPK
RVFLSEETNDNVLDCIIKKVKIIHVDPNMDPEGKAQLVADSEAELYYDMSYAVAYSTFANIP
SDTNENSGISSDADLEAGTPPVRTAALLDLYSGCGGMSTGLCLGAALAGLKLETRWAVDL
NSFACKSLKYNHPGTEVRNEKAEEFLALLKEWAILCDTYVHVNNSESDSPIEDEEEDDEPL
AKDEFVVEKLLEICYGGSGRGKGIYFKVQWKGYGPEEDTWEPIGNLSDCQLKIKEFVQEG
HKRKILPLPGDVDVICGGPPCQGISGFNRFRNRKEPLKDEKNQQMVTFMDIVSYLKPKFV
LMENVVDILKFADGYLGRYALSRLVALNYQARLGMMVAGCYGLPQFRMRVFLWGALPD
MVLPKYPLPTYDVVVRGIVPNAFSQSVVAYDETQKPTLKKALLLGDAISDLPEVDNYQLH
EVMEYGTKPKTEFQRYIRLGRKDMLDHSFGDNTCPEEGKLLDHQPLRLNQDDYDRVQQI
PVKKGANFRDLPGIKVGANNIVEWDPEVPRVYLKSGKPLVPDYAMSFIKGRSLKPFGRLW
WDETVSTVVTRAEPHNQIILHPNQARVLSVRENARLQGFPDYYRMNGPIKEKYIQVGNAV
AVPVARALGYSLGQAYQGEMESTGPLFTLPASFTNVGQTEELARASSVGIPVGEVVEQ

>BdCMT1
METPPHNPISPPSPPPVTDGSPAPYAGADGDEAAGGFDGSLESMLERIFGPPELEPWSPPRE
FDFAAEFAAVAATDPDPLEDLGGGPWDAAPWMSTGLVACEGAAATTRAPSPPTAAPGFAP
APTPAFDSSEGAPEVRSLDHLDNYSPMPESPSTPVAVDMREKLVFVSDSAPVLESSPSPPVA
CPDLSTTEPMKETTNAAPERSDLPLPNRVEQSTSSLSNQVVLALQTPQVVREMPQEVEGK
SSRPLKAKRVRGSTDKCLPNLKRAKKNSGSIGRLPNVTTMPDPGNTHEVILDKHLTDSEM
VESDDGSCFFVGEAVLEEEAKQRWPHRYKKNHHFVEKFLSLKDKRSGNQPFANAGNDVL
DVKCHYLQASVSGSTLCVGDCAFVKGPEGRPNYISRILEFFETVAGECYCRVQWFFRAED
TVMEDQAQSNDPRRLFYSDLQDDYSLDCIVSKLTIVQVPPCVDKESKSIPSSQYYYDMKY
SLDYSTFSTLEMEDTNAILQSSHASSIEMKKVDFSKKQKSPVPNKKDLSLLDLYCGCGGM
STGLCLGAHGAGVNLVKKWAVDDDLVACESYRLNHPETRVRNETTNDFLLLLKEWQKLC
KKYVEQSEVKDHADGLTETINEIPDDSVDPTEELEVWKLVDICFGDPNGIENHCLHFKVR
WKGYGRNDDTWEPIDGLGNCNEAIRDFVIEGHKRKILPLPGDVDVVCGGPPCQGISGYNR

NREFDAQFKCEENKEIIFFMDVMQFLKPKYVYMENVLDILKFADATLARYALSRLVAMRY
QAKLGIMAAGCYGVPQFRMRVFLLGCHPEEKLPPFPLPTHEAIRKNGCPLAFERNLVGWS
DGTTVQLAKPIVLEDILSDLPEVGNEESRDEMPYVKDPQTEFQRYIRAFNSEVRGPKSRAA
KSKSRKAKPKLYDHRPFVLDNDNYLRVLQIPKKKGANFRDLPGVVVGPDNVAKLDPTKE
RILLPSGNPLVLDCILKYEDGKSLRPFGRLWWDEVVGTVVTCPNAHTQAFIHPAQDRLLTI
RESARLQGFPDSYRFHGEVKERYCQIENAVAVPVGRALGYALAMAYLNKTGSDPLMVLPP
KFAFSQNIEGTL

>BdMET1
MVKTKSPRSPVTTGTKRCRAKPQKKKEESTGEGQLETEPKDSAEVMHNEVENGAGSAAR
KRPRRAAACSDFKEKSVRLSEKSNVVMIKKNRMEEEEIDAINLTKLGPEDSPPCRKLIDFIL
HDADGNLQPFEMSEIDDFFITALIMPVDDDLEKERERGVRCEGFGRIEDWAISGYDEGTAV
VWLSTELADYECVKPASNYRSYYSHFYEKAQVCVEVYRKLMRSVGGNPNLSLEELLATV
VRSINAIQGYSGTMSKDFVIATGEFVYNQLIGLDQTAGNDDEKLATLPVLLALRDECKSRV
EFNKMPPKISNGSLKINDAECNEVAEDDDEKLARLLQEEEEWKMMKKQRGKRGVPAQK
NVYIKISEAEIANDYPLPAYYKPSTQEMDEYIFDGDDGMFSDDVPVRILNNWVLYNADSR
LISLELIPMKSGTENDIVIFGSGFMRDDDGSCCSTAESANSSSSSSKAEHQDAGVPIYLSPIK
EWLIEFGGSMICITIRTDVAWYKLRQPIKQYAPWCEPVLKTARLAVSIITLLKEQSRASKLSF
VDVIKKVAEFDKGDPAFISSNISLVERYIVVHGQIILQQFADFPDETIRRSAFATGLLMKMEQ
RRHTKLFMKKKAQVTRGENLNPIATMGTSSKRKAMRATTTRLINRIWSDYYAHHFPEDSK
DGDENEAKEIDDEQEENEDEDAEEEVQIEEEKVSETPPSTRSRKLVSQTSKEIRWEGKSTG
KTASGEALYKCGYARELRIAVGGTVTLEDDSGEIVICFVEYMFQKPDGEKMVHGRMLQK
GSETVLGNAANERELFLTNDCLEFELKDIKELVSVNLQSMPWGHKYRKENSEADKIERAR
VEERKKKGLPMEYLCRSLYWPEKGAFFSLPHDKLGLGNGVCSSCEHREPERDELRILSKT
SFIYRKVTYSVHDFLYIRPEFFSQEEDRGTYKAGRNIGLKPYAVCHLLDVCEPVGSKKINPA
SAKVSVRRFYRPDDI
SSDKAYTSDIREVYYSEDIINVPVDMIEGKCDVRKKIDISNSDLPVMVEHVFFCEHIYDPMT
GALKQLPPNVKLTSMVQKAAGALKKNKGKQICENDQVDSDKRKEVPKENRISTLDIFAG
CGGLSEGLQQAGASFTKWAIEYEEPAGEAFRQNHPEAAVFVDNCNVILKAIMDKCGDAS
DCVSTSEAAEQAAKLAEENIKNLPVPGEVEFINGGPPCQGFSGMNRFNQSPWSKVQCEMI
LAFLSFAEYFRPRFFLLENVRNFVSFNKGQTFRLAVASLLEMGYQVRFGILEAGTFGVAQS
RKRAFIWAAAPGEILPDWPEPMHVFASPELKITLPDGKYYAAAKSTAGGAPFRAITVRDTI
GDLPKVENGANKLILEYGGEPTSWFQKKIRGSTIALNDHISKEMNELNLIRCKHIPKRPGC
DWHDLPDEKVKLSSGQMVDLIPWCLPNTAKRHNQWKGLYGRLDWEGNFPTSVTDPQPM
GKVGMCFHPDQDRIITVRECARSQGFPDSYQFAGTIQSKHRQIGNAVPPPLAFALGRKLKE
AVDAKRQQA

>BdMET2
MEKVPHTAVTTGTKRRRAKPREMADESIEDDRLETEFANDKKESNGATENGDRPIAHKRR
KRAAACSNFKEKAFDLSEDDSLVAIKESRVEEEIGAVRLTKTGPEDKKSCRKLIDFILHDEN
GNAQPFEMSHSDGISITALVMPLDDNMEKDREKGIRCLRFGPIKNWAISGYKQGTAAIWLS
TELADYKCVKPASGYRSYFDHFSRKADVCVEVYKKLARSVGGNLQLGLEELLASVVRSI
NSNRSFNGTVNKDFVISIGEFIYNQLSGLDNTSDKDDETLSELPVLVALRNECTSRIEFNKL
PGMTSSETLKIKDGQCKEEVTRNEDEDEKLARLLHEEEEWKLMKQRSKRQASKKNVYIK

ISETEIANDYPLPAYYKPYSLEMDEYMFDSDMGMFSDDVPVRILNNWALYNSDSRLISLEL
IPMKSGAENDIVIFGSGFMREDDGSFFSTAEPTQLSSSSSKSDQEDQGVPIYLSPIKEWVIEF
GGSMICISIRTDIAWYKLHQPLKQYAPWCDTVLKTARLAVSVITLLKGQSRASKLGFADVI
KKVAEFESGHPAFISSNATLVERYVVVHGQIILQQFANYPDPSIRRSAFVTGLTAKMEDRRH
IKLVMKKKSQPTRGENLNPSANIGPIIRRKLMRATTTRLISKIWGDYYATHFPEDSKEGAED
EPKEIEEEQEENEEDAEEEVKVEEEHVLSTPPSARSRKSSSNNCKEIEWEGQTVGKTRSGD
DLYKCARVRELNISVGGAVKLEDDSEDALMCFVEYMYEKQDGTHMIHGRILQKGSQTVL
GNAANEQEVFVTNDCLEFEIGDIKELVSVNVQLIPRGHKYRKENLQANRIERAKAEERKK
KGLPMEYICKSLYCPEKGAFFSLPYDKLGTGTGICSSCQQREAVGDEFKILSETTFVFKNST
YAVHDFLYVRPEFFSRVDGHETYKAGRNVGLKPYVVCHLHSINAPAGSKKVNPESTKVSV
RRLYRPDDISSARAYSSDIREVYYSEDMLSVPVAMIEGKCEVTTKNNLPDSNLPVVVDHVF
YCEYLYDPDTGALKQLQSNVKLMTIARKAPTSKKNKGKQICDSDQAGSDKQKAAASENS
LATLDIFAGCGGLSEGLQLSGASHTKWAIEYEEPAGQAFGENHPEAAVFVENCNVILKAIM
DKCDDTDDCISTSEASEQAAKLSDEKIKNLPVPGEVEFINGGPPCQGFSGMNRFNQSPWS
KVQCEMILAFLSFAEYFRPRFFLLENVRNFVSFNKGQTFRLTLASLEMGYQVRFGILEAGA
YGVAQSRKRAFIWAAAPGETLPDWPEPMHVFASPELKITLPDGKYYAAAKSTAGGAPFRSI
TVRDTIGDLPPVENGASKPTIQYGSEPISWFQKKIRGDVLSLNDQISKEMNELNLIRCKHIP
KRPGCDWHDLPDEKVKLSSGQMVELIPWCLPNTAKRHNQWKGLYGRLDWEGNFPTSVT
DPQPMGKVGMCFHPDQDRIITVRECARSQGFLDGYRFAGNIQSKHRQIGNAVPPPLAYAL
GRKLKQAINARN

>BdDRM1
MVDLISDSDDSVAFEWESDGDAEPSSAPVLRDFDAPGPSTLVRQDTNGRPNGEALPASLVE
EYVGMGFPKDMVLKAIKQIGNLSSNNFHFICLVLFAPCVSTDSFAFRSRDANSLVELLLTY
KVLAESDASVGNPSSSGCAPHSVENDDDDNLDSEDWDDDDDSDGSSDEDFLQEMSKED
DKAKSLVDMGFPVEEAKMAITRCGADAASIVLVDSIYASQAADSGNVFDHEVSDRSFRSF
GERKRAISIEGSKKKAKRYGGGAQGNRTPLDGSDDEMPLPNPMNRRLPVLATAPPFFYYE
NVARAPKGEWREMSRNLYDIHPEFVDSVHLCAAARKRGYIHNLPIVNRSPILPLPPKTILEA
FPHYSKWWPSWDSRKKLNCLQTSGASAKLTERIQLTLANSTNPPPPHVQKYVLNQCKRW
NLVWVGKNKVAPLEPHEVEYLLGFPKDHTRGICKKEREKSLGNSFQVDTVAYHLSVLRD
KFPNGMNVLSLFIGIGGAEVALHRLGIRLKTVVSVEISPANRRILKGWWDQTQTGTLIEIED
VKTLKSDTIASFVSKYGGFDLVIGGSPCNNLAGSNRHHRVGLEGEHSSLFYHYPRILGDVK
RAMDGR

>BdDRM2
MDCISDSDDSAKFEWESDNEAEPSSAPVLRNFDAPGPSTDANGWANGDAPSTSLVEEYVG
MGFPKEMVLKAIKEIGHNDANALLELLLTYKVLGEDPTVGNCSTLGCAPQSVEDDDDGD
LDSEDWDDEDDADGGEPNFDSSGDEDFLQEMSEHDKKIKSLVDMGFPEDESNMAIVRCG
VDAALTVLVDSIYASQAAGDCNSRNSSHHEVCDSFGGRRNKKKRKQYGGGAQGNRPSEC
HEELMPLPNPMVGFSLPTARLPSVSRRLPKQATGPPFFYYENVALAPKGVWTIISRNLYDIA
PEFVDSKYMCATARKRGYVHNLPIENRSPLLPLPPKTIFEAFPHYKKWWPSWDPRRQLNC
VQTCVSSAKLTERIQCALARSGDPPPLHVQKYVMHECRKWNLVWVGKNKVAPLEPDEM
EYLLGYPRDHTRGIGKTARYKCLGNSFQVDTVAYHLSVLRDIFPNGLNVLSLFTGIGGGEV
ALHRLGIHMKTVVSVEISEVNRRILRGWWDQTQTGTLIEIPDVQSFTSDKIRSFIRRFGGFD

LIIGGSPCNNLAGSNRHHRDGLEGEHSSLFYHYPRILDTVKDVMAGM

>BdDRM3
MARTKRPAKRKPKGAANLKLPVNSRHCGSSSRFMEYAALEADLIKMVKTEDQDEDEGVS
ASVGALRDGTVDPQPDTAHAGVKAEEDGQPSSSSSYSKFIGMGFPPTLVDKMLQKHGDK
DFNAILESLLFHSSLTKSGSESSSSLGSLFDSDSEENISRLGSMKEPHEEIKPEPDSFSERRSY
LLSAMNFSQQEVDWAFNQLGEEAPLDQLVDTIVTAQVAGFTGGNENVDATTEGKAESLF
GVMEKTLHLLQMGFTEEEVSGAIDSSGQEATVQELADSIFARRIDNSIKQTEVKIESDFLGG
TENQHSTCHQRLRYYDDEDDKIGVKRAKSIFTDNSSGASSSRPGNQPSLTPWLSGCTGSVS
NGYVKEEFDAMASGPRPDVRPEIAKPPYFLYGNVVDIPKGTWHQLSDFLFNVEPEFLNSQ
YFSAVMRREGYLHNLPMETRHIVVPKSPMTIEDALPFTRQWWPSWDTRKHIGVVTLDVA
EIEQMCEKLGKIMTDSRGVLSHEKQAHIMQQCKMSNLIWIGKDRLSPLEPHQLEHILGYP
RNHTEQFELNTPDRLAAMKYTFQTDVLAYLLSVLKSKYPNGLRLLSIYSGLGGAEVALDR
LGIPIKCIVSVEESDVNRKILRKWWRRTNQAGELRQFVGIWKLKTIVLEDLVKEFGGFDLII
GGNYSSCRGGRTINATMGMDSNKFYEYARVVKIVRALHNS

>RcCMT1
MGRSAKRPNREPEVEEEDGATLSDQKGPITLLSARPKETKKAKLDADLSFIGNPISATEAR
KKWPQRYKSQISKVKNGSEPQNGVLSKDDDVTQAKCHYKQAMVDGILYDLGDDAYVK
AEDGKPDYIARIVEMFESIDGEPLFTAQWFYRAEDTVIKDYVKTAESRRVFLSEIRDDNPL
DCIVSKVKIALVEPNLDLAEKERNLPPCDLYYDMKYTLPFLTYETIKTDDSGRDSGSSSTIS
SENDSNNSIDDVKVTTAKPLKVLSKVHSSEKSELYLLDLYSGCGAMSTGLCMGASLSGVK
LVTKWAVDINAFACKSLKTNHPETEVRNEAAEDFLSLLKEWEKLCRKFSLFGSEKHPEQSS
NSASDEEEEDEEEEEEEKGKDKDDDDDEIFEVEKLLAVCYGDPNKVNKRGLYFKVRWKG
YGPSEDTWEPIEGLSDCKDKLKEFVTKGFRSKILPLPGDADFICGGPPCQGISGFNRFRNTK
APLDDPKNHQMVVYMDIVEYLKPKYVLMENVVDILRFAGGFLVRYALGRLISMNYQARL
GMMAAGSYGLPQFRMRVFMWGSQPSESLPQYPLPTHEVVVKGGIPNEFEEITVAYNKLDP
CQLEKALYLGDAILDLPPVNNDESQDERKYGTTPQSDFQKYIRLKKSDVVGFATDKNASH
PQMLYDHRPLKLNDDDYQRVCRVPKKKGANFRDFPGVLVGTDNKVEWDPAVERVLLPSG
KPLVPDYAMSFVRGTSCKPFGRLWWDETVATVVTRAEPHNQIVIHPMQDRVLSIRENARL
QGFPDCYQLHGPVKERYTQVGNAVAVPVATALGYSFGIASQGFSDNKPLTTLPFKYPNCLQ
KSS

>RcMET1
MVSMREVSESKEAPFSDNNKKTGAAEDTSNIQESKPENGDGAASKVKLKRGLSKNVENV
YSSRKMTKRAAACSDFKEKPLDFSEEYSFTEIKKEVIVEHEAVALGLTAGRDDLRPNRRLK
DFIFHDAYGNIQAVEMLEFFDLFISGLIVPLEESSDKDKEKGIRCNGFGRVESWAISGYEEGS
PVIWVSTDMADYDCIKPANRYKKLYNLFFEKAHACVEVYRKLSRMHGGDPNISFDELIAG
VVRAMSGSKNFPQGMSIKDFIISQGEFIYNQLIGLDATSKKNDQMLTGLPVLLAMRDEKK
KREGLFMHEATTTGRTVNRNLTIDGKLTNQLLSSSLAEEDEDMKLASLRQEVENWLSMK
QNKRRFSVPSSHFYIKINEDEIVNDYPWPTYYKTSAEEIDEFLVICDDEIFNPPENVLHNWT
LYNSDARLISLELLPMRYCADADMTIFASGIVTKDGDLSGRYLDVNSDLPSSASSDVQDVG
LPIYTSAIKEWKIEFGSSMIFISLRTDLSWYRLGKPSKQYAPWYKPVVKTARLAISIITLLQE
QRRVSRLSFADVIQRVSKFNEGHHAFISSLAAEVERYVVVHGQIILQLFSEFPVKAIQNCPF

VTDLHSKMQQINHLKLRVKEKRVLKKGLNLNPREAVATISERKAMAATTTKLINRIWGDY
YSNYSPEESNGADGLEIKEDDEVVEEQEESEEEDSEEIEEQKMEIEKKSEKTRSTEKLNTW
QCSNQEIRWVGGLLGKSCTGKAFYKKAFVHGGIVAVGDFVMLGTTDADQLSPIYFVEYM
FENSDMRKMAHGKLMLYGSQTVLGNTANKRELFLANDCLEFELSDVKQNVVVEFKLTP
WGYRHRKASIIKDRTDRERTEERRCKGLPMEYFCKSQYLPERGAFICLSKDTMGLGNGVC
HSCKTKEIQMGKESFRLNLSSDSFVYKGTEYCIHDYVYRAPHHFTVDVMDLQTFKGGRN
VRLRPYVVCQLLEIQVGKGLLQVTPESILVKVRRFFRSEDISAENAYCSDIREVYYSEHIET
FPILDIQGKCEVRKKHDLPSFDKLAIFEHIFFCEHIYDPEKGTIKQLPAHIKLSSSKERMNDD
ILYRKKKGKCKEGENDFDDNVKRSEALSRNQLATLDIFAGCGGLSEGLERAGISVTKWAIE
YEEPAGEAFKQNHPEALMLINNCNVILRAIMSACGDADDCICTSEASELAEKLDEKEISNF
PRPGEVEFINGGPPCQGFSGMNRFNQRSWSKVQCEMILAFLSFADYFRPKFFLLENVRNFV
SFNKGNTFRLTLASLLEMGYQVRFGVLEAGAFGISQSRKRAFIWAASPEEILPEWPEPMHV
FAGPELKIKLSGNSQYAAVRSTANGAPFRAITVRDTIGDLPAVGNGASLTTMQYKNEPVSW
FQKTIRGDALVLTDHISKEMNELNLIRCKRIPKHPGADWRDLPDKKVKLSNGQLVDLVPW
CLPNTAKRHNQWKGLFGRLDWEGNFPTSVTDPQPMGKVGMCFHPEQDRILTVRECARSQ
GFADSYQFLGNIQQKHRQIGNAVPPPLAFALGRKLKEAVEMKRSLSVRCLCNYCTAIVVQ
NSLPSTYP

>RcCMT2
MNKFYGNDLHYDQLLQGEGGQKHVGRIVEFFKTTDGEDYFRVQWFYRAEDTVIKEEAG
FHDKKRLFYSTIMNDNPIDCIISKVRIVQLPSMIGSKSSIRSTDFYFDMEYNVDYSSFRTLLI
GKHFCLRDNSSSPNVVEAVSTTGGNTSLENMRSSESNKAELALLDLFSGCGGMSTGLCLG
AKVSCVDLVTRWALDSNKSACESLKLNHPETNVRNEAAEDFLELLKVWEKLCKRYAVKD
IKSMQQSRSIASRVAEENDNSLSDNDITPGEYEVSKLVDLCYGDPDNIGKRGLKFKVHWK
GYSTSEDTWEPVEGLRNCQECIRDFVRKGFKSKILPLPGDVDVICGGPPCQGISGYNRFRN
VNAPLSDERNRQIVVFMDIVQFLKPKFVLMENVVDILKFDKASFARYALSRLVHMKYQAR
LGTIAAGCYGLPQFRLRVFLWGAHPSEKLPQFPLPTHDVIVRYWPPNEFERNTVAYDEGQP
RELEKAAVLQDAISDLPPVTTHETREEMAYEKPPETEFQRFIRSTEHEMTGSSQRGTTQMN
NLLSDHRPYALTEEDCARVCLIPKRKGANFRDLPGILVGNDNVVRRDPTKEQVLLPSGKPL
VPDFAFTFEQGKSKRPYARLWWDETVPTVVTYPSIHSQAMLHPEQDRALTIRECARLQGF
PDYYRFSGTVKERYRQIGNAVAVPVGRALGYALGMAFLKLSGDGPLMTLPPKFSHSTNLQ
LAKSLFQKTD

>RcMET2
MKKNKGKPKSSVSKANKEQINNIKEKKRSSSQKNEEPAGSKKRPKRAAACTDFKEKAVR
LSDKSSVLESRRDQFADDEILAVHLTHGQDDGRPNRRLTDFVVHDENGTPQPLEMIEVDD
MFISGLILPLDENPDKEKEKRVRCEGFGRIEGWDISGYEDGFPVIWLTTDIADYNCLKPAN
NYKKFYDHFFEKARACIEVYKKLSRSSGGNPDLTLDELLAGVVRSMSGSKCFSGAASIKD
FVISQGNFIYKQLLGLDETSKNNDQKFADLSALLALRDKSEEHGNFVLAKAVNTSGNLTIY
QKFGDSVSNVNQSISSTAAGEDEDAKLARLLQEEEYWQTTKKQKKIHGSASSSNTIYIKIN
EDEIANDYPLPVFYKHSDEETDEYIAIDTEEHIMVDPDELPKRMLHNWSLYNSDSRLISLEL
LPMKPCEDIDVTIFGSGRMTEDDGSGFSLDDDPDQSSSAGSGAQDDVGLPIFLSAIKEWMI
EFGSSMVFISIRTDMAWYRLGKPSKQYTSWYKPVLKTAKLARSIITLLKEQSRVSRLSFGD
VIRRVSEFKKDDHGYISSDPATVERYVVVHGQIILQLFAEFPDEKIKKCAFVVGLTSKMEER

HHTKWVVNKKQILQKNQPNLNPRAAMSSMAPVVSKRKAMQATTTRLINRIWGEYYSNY
SPEDLKEATNCEAKEEDEVEEQEENEDDAEEEKLLLSDKTQKACSMSSRTKSYSKDEVLW
DGNPVSKTHSGEAIYNSAIVRGEVIKVGAAVYLEVDESDELPAIYFVEYMFETSGGSKMFH
GRVMQHGSGTILGNAANEREVFLTNECLNYELQDVKQAIAVEVRKMPWGYQHRNDNAT
ADRIDRAKAEERKKKGLPLEYYCKSMYWPERGAFFSLPFDSMGLGSGICHSCKVKEVEM
EKYIFRVNSSRTGFVHMGTEYSIHDFVYVSPCHFTIEREAETYKGGRNVGLKAYAVCQLLE
IVVPKEPKQAEATSTQVKIRRFSRPEDISSEKAYCSDIREVYYTEETHLLSVETIEGKCEVRK
KNDIPPCGSAAIFDHIFFCEHLYDPSKGSLKQLPAHIKLRYSTGTQESDAASRKRKGKCKEG
EDEVENKREATQGRRLATLDIFSGCGGLSEGLQQAGVSSTKWAIEYEEPAGEAFKLNHPES
LVFINNCNVILRAVMEKCGDTDDCISTSEAIELAASLDEKIINDLPLPGQVDFINGGPPCQGF
SGMNRFSQSTWSKVQCEMILAFLSFADYFRPKYFLLENVRNFVSFNKGQTFRLALASLLE
MGYQVRFGILEAGAYGVSHSRKRAFIWAASPEEVLPEWPEPMHVFSAPELKISLSGNSHY
AAVRSTANGAPFRAITVRDTIGDLPVVGNGASATNMEYKNDPVSWFQKRIRGNMVTLTD
HISKEMNELNLIRCQKIPKRPGADWRDLPDEKVKLSTGQLVDLIPWCLPNTAKRHNQWK
GLFGRLDWEGNFPTSITDPQPMGKVGMCFHPEQDRILTVRECARSQGFRDSYKFAGNIQH
KHRQIGNAVPPPLAYALGIKLKEALDGRLK

>RcDRM3
MQLISFSNSNAVLSWLTAEASGHDKMRKVHNGETSSNQKTTVPKPEMLDFDFPSDFIYSS
QLGETAASSSASDVRSFLTGMGFLPSLVDKVIQENGEDNVDLLLEILMECSDPQKSNSQSS
DSLDTLFNDKDASNPPEYSKFGQPKEEPDVFGFDDSKRASLLHMNFSAEEVDFAMEKLGT
DAPVDEIVDFITAAQVAANFDEERDDELEHDVEQNKYCSRHSRMNPSHVARGGESFNMEI
RDEQRRDSYGRKKPENLSQEAVSRSSNLNLEEIHRGKRPKQEYTEGYSNTQDEHFGFEEN
YEEEGPKPEYTADSSFSFESAWVEEKVDTEITGFRIPREPKFKSCKSVDRMVAKPPYFLYGN
VATVSLDTWGKISQFLYAVEPEFVNTQFFSALSRKEGYVHNLPTENRFHILPKPPMSILDAM
PHIKKWWPSWDTRKQLSFINYETNGLSQLCDRLGRMLTDSRGLISSQQKRDILHHCHKLN
LVWVGLHKVRPIEPEHLELILGYPLNHTQTTEGSLTERLHALRYSFQIDTLGYHLSVLKSM
FPEGITVLSLFSGIGGAEVALHRLGIHMKGVVSVETSETKRKILRMWWRNSGQTGELEQIE
DIQKLTTKKIDRLIERFGGFDFVICQSPCTCASGNPKMEQRADARSALDFSLFCEFVRVLQR
VRTQVQRKR

>RcDRM2
MGVIVYCFRITTCKPVYMEHKTLYLEILGIAVASKEMDGDSFCGEGDNFDWDSEDEREIEN
FGLTNSSCLAVPAVEATARSVEKCLMGFSCILRHASSSVRSSGTKVMDHFVGMGFPEKMV
AEAIQENGEENADLILETLLKFSTISSPSSSGSKLIDHFVGMGFDVEMVDRAIQENGEEKTD
SILETLLTYSVIEKSHQEQPHVDSDHWSSEYEGSFLNDFSDIDSSENEENGKSQPVEGNTLR
FLARMGYTTDEASIALERCGPDANIAELTDFICAAQMAKAADASFPEEKPKIRHFDDDYP
KNKKRNYAEYDMWKKKKQMKLDKKLLNGDDELIRLPNPMLGFGVPTDPAIVTHRKLPE
AALGPPFFYYENVALAPKGVWQTISRFLYDVEPEFVDSKYFCAAARKRGYVHNLPIQNRY
PLLPLPPNNIHEALPLTKRWWPSWDTRTKLNCLQTCVASAKLTDRIRKALEDYEGEPPLSIR
KYVLDECRKWNLLWVGRNKLAPLEPDEVEMLLGFPRNHTRGGGISRTDRYKSLGNSFQV
DTVAYHLSVLKDMFPEGISLLSLFSGIGGAEVALHRLGIRLKRVVSVEISEVNRNIMRCWW
EQTNQTGTLIDIADVHDLNADRLEQLMSSFGGFDLVVGGSPCNNLAGSNRHHRDGLEGK
ESSLFFDYCRILDLVKCIMTRN

>RcDRM1
MDRFLSLVNVNVLLKHVTLKRQNSILRSQANSLTGCSSSRMVHQFVNMGFSEKLVMQAI
DENGEDEASILDSLLTYSLRKEDFPCKKRKLHQNRSSKSQESKRKAKNFLERNGRTLHLPN
PMIGFGIPHEPWKITERKLHPIAVGPPYFYYENAALTPKGVWSKISSFLYDIQPEFVDSIYFC
AAARKRGYVHNLPIQNRFPLLPRPPLTIHEVLPSTEKWWPSWDKRTKLNCLLTSYGNAKL
TECIRKALEDCDGEPTLRAKKYVLEQCKRWNLVWVGKNKVAPLEPDEMEMLLGFPKDH
TRGGGINRNDRYKALGNSFQVDTVAYHLSVLKNLFPYGITVLSLFSGIGGAEVALHRLGIP
LNSVVSVEISEVNRNIPRSWWEQTNQKGYLIEIGDVREVDGDSIKRWIKLFGGFDLVIGGS
PCNNLAGANRRNRDGLEGEQSSLFYDYYRILKLVRDTFGEIMDSEILNRILSYPDL

>PtCMT3
MPRKRQRSTASKPETSSSSPPKTRKMMSTTTAPKQSRLNEKKAEEEQSTASTRSRLKEKKA
EKEQSKAAARSCLKEKKAAEEQLTTATRSRLMEKEAEEEQSTAPNRSRLREKKVKKEETE
EVFLDAEEDDTNSIDEVGAKMGTDNGDSRSETKEKEKEKRGSGKTKKISTPEKAKEEDGT
PARFVGNQVPDAEARKKWPHRYANKIKNKTPISKPSNSLDDSEEIIKARCHYKRAEVDGII
YNLYDDAHVQASDGEADYICRIIEMFESEDRTPHFTAQWYYRSTDTIIKDKYISDPKCVFFS
EIRNDNPLDCLTRKLKIVRLALGVDSETRRAKTLNCDFYCDMLYLLPYSTFVKLPSENNTT
APESSSTISSDIDAAGVKFECDEVCESSGRRKSEVALLDLYSGCGAMSTGLCLGANLSGLN
LVTKWAVDLNKHACESLRLNHPETQVRNETAEDFLMLLKEWEKLCIRFSLVKNDDPEKQ
QTYSFDMDDEDDDDDEEEEDDDNNDVSDNNDDSEVFEVEKILEVCHGDPKEIGGQRDLY
FKVSWKNYGPDYDTWEPISGLSNCREAIKKFVMHGYKSNILPLPGDVEVICGGPPCQGIS
GFNRFRNVKNPLEDPKNKQLVVFMDIVDFLKPKFVLMENVVDLLKFADGFLGRYAMGCL
VSMKYQARLGMLAAGAYGLPQFRMRVFLWGACATEKLPQYPLPTHDVLVRGVVPLEFE
GNTVAYEEGVKPQLERKLFLEDAISDLPAVANDEKRDEMPYGESPKTEFQRMIRLKKMGL
ELNDLLFDHRPLELNDDDYQRVCQIPKRKGGNFRDLPGVRVRPDKKVEWDPEVPRQYLS
SGKPLVPDYAMTFVNGSSSKPFARLWWDETVPTVVTRAEPHNQAIMHPEQDRVLTIRENA
RLQGFPDYYQLCGPIKERYIQVGNAVAVPVARALGYALGRAFQGFAGDDPVFSLPKKFPRI
TEDPSSSSEAQC
>PtDNMT2
MEEKKGLLPKKEEEPWRVLEFYSGIGGMRYSLMKAGVNAKVVEAFDINDKANDVYEYN
FGHRPYQGNIETLTAADLDNYGAH
TWLLSPPCQPYTRQGLQKQSGDARAFSFLKILELIPHTKQPPNMLFVENVVGFETSDTRAK
MIEILASSEYITQEFILSPLQFGVPYSRPRYFCLAKRKP
LSFHHEVCNNKLLWSPSPLFEHNENNVTDGWDQPPVNWDTLIHSCEPVERFLEFKSSSNR
VTGGTGTDVSADCSGALENIDERNLNTMSQYIVPLSLIER
WESAMDIVYPDSKRCCCFTKSYYRYVKGTGSLLATIQPNIKGKESSLKKQGLRYFTPRELC
IIGKQFKRSSGCALTPLSIFSTIMIFQSKSPIYRFMAFS
FNFCTTPNFPCKGS*
>PtCMT2
MKSSSSPTDSRKLTPLQVYESTSTSRRSPRLAPPNNNVPSKTPKSNKPKTTHISLRRSPRLEP
TPASPQSVSRKKHSRNVSKTKCSARNVSVDRKIPSRSSRVGNSRMQNVAANRTVLLDSSA
LRRSPRLSSADMEIVVSERNASVVVADTARLRRSPRLANGYAEVAVSKVRTFLKRKRVSDE
MSRSPRNQNGRLDVQFALVQSEKCSRFIVNEENERNCLKVECSSKDLSKGSPVACSSANA

KLGCCEKRGCQSVLLTISDSEVGDSGEFEAKPLLLPWHGDNESQTGMVKCDDGSSVFEEE
PLRMSPSFDFANENAVINESPIKSCKWSSALDGDGNFEVISCSSGIFDSNDACPSKRVKICEE
NSVSGMSDEKLLRRSPRTNSIVVTENGENKSGKKQSPKTNSGKELSLEKASSGKKQKEHR
GNCSLIGDPVAHDEAQERWHWRYEMKSKRTKHPRLALDDDDEDKVVWNVECHYTQANI
EGRIINLGDCVYVKGEGAKNHIGSILEFFKTTDREDYFRVQWFYRAEDTVMKEAADFHD
NKRLFYSTVMNDNPIDCIISKVTVVQISPRVHLKFHSTPASDFYFDMEYCVDYSTFRTLLTD
CSLRGHELSPLPFCDSRSATPSDISMENMSTCGSYKAKLTLLDLFSGCGGMSTGLCLGAKV
SCVDLVTRWALDSDESACQSLKLNHPETHVRNEAAEDFLELLKEWQKLCKRYAVNDVGR
THKSRSMASSMSKQNKNSSNDDDIASGEYEVARLVDICYGKTDKRGKRGLKFKVHWKG
YSTSEDSWEPIEGLSNCEHSIRDFVREGFKSKILPLPGDADVICGGPPCQGISGYNRYRNVD
SPLADERNIQIVVFMDIVQFLKPKYVLMENVVDILRFDKASFARYALSRLVHMKYQARLG
TVAAGCYGLPQFRLRVFLWGAHPKEKLPQFPLPSHDVIVRYWPPPEFERNTVAYDEDQPR
DDLEKATVLRDAISDLPDVTSHETREEMAYDKPPETDFQQFIRSTRNEMTGSELSGTRMIN
LLYDHRPYSLTEEDFARVCQIPKKKGANFRDLPGVVVGADNVARRDPTEEQMLLPSGKPL
VPDFALNFEGGKSRRPYARLWWDETVSTVVTFPDLHSQAVMHPEQDRVLTIRECARLQGF
PDYYRFCGTVKQRYRQIGNAVAVPVGRALGFTLGMAFQKLSGDDPLMTLPPKFSHSTNLQ
LAKSLFQKAE

>PtMET1
MRKNKKGKQKSSVSNAKKEVPEKTAKGKKRNFSDTNKEDPAGGSLTRPRRAAACKDFK
EKSLRLHEEKSSVVESKKEQVVNEEILALRLTQGQEEGRPNRRLIDFVVHDANGNPQPLE
MIEVDDMFISGVIMPLEESLDKEKEVPVRCEGFGRIEAWNISGYEDGSPVIWLTTEVADYD
CIKPSGGYKKFFDRFFQKALACIEVYKKLSRFSGGNPEFTLDELLAGVVRAMSGNKCFSG
APSVKNFLVSQGEFIYQQITGLDQTSKKNDKFFSDLPALVALRDESRNHGSVLLAKAANPG
GNLVIDPKSVDGAIVNQSNQSSTIAEEDEDAKLARLLQEEEYWHSNMRQKKSRGSASASN
TIYIKINEDEIANDYPLPVFYKHSDEETDEYVVVASDDVIDHPDDLPRKMLHNWSLYNSDS
RLISLELLPMKPCEDIDVTIFGSGRMTEDDGSGFCLDDDPDQSSSRGSEAQDDMGLPIFLSA
IKEWMIEFGSSMIFISIRTDMAWYRLGKPSKQYGSWYKPVLKTVKLARSIITLLKEQSRVSR
LSFADVIRKVSEFKKDHHAYISSDPAAIERYVVVHGQIILQLFAEFPDQKIKKCAFVVGLTR
KMEERHHTKWVVNKKAIVQKFQSNLNPRAAMDTVAPGSKRKLMQATTTRLINRIWGEY
YSNYSPEDLEEGAECEVKEEDEAEEQYENEDDDKEEVVEKTLKPRSVSERTKSHTSQKEV
RWDGNPVSKTSSGEAIYKRAIVCGEVIVVGDAVLVEVDESDELPAIYFVEYMFETRNGSR
MFHGRMMKRGSETVLGNTANDREVFLTTECMNYKLQDAKQAIILEVLKRPWGHDHRKD
NINADRIDREKAEERKKKGLQVEYYCKSLYWPERGAFFTLPLDTMGLGSGVCHSCNLKIA
EEDKDIFRVNSSQTGFSYKGTEYSVHDFVYVSPHQFASERGENETFKGGRNVGLKPYVVC
QLLEVVLKEPKQAETRSTQVNVQRFFRPDDISPEKAYCSDIREIYYSEETHLLSVETIEGKC
EVRKKNDIPTCSAPAIFDNIFFCEHMYDPSKGSLKQLPAQVKSKFSAVSRDGDVASRKRKG
KSKEGENDIEADKQREASPENRLATLDIFAGCGGLSEGLQQAGVSSTKWAIEYEEPAGEAF
KLNHAGSLMFINNCNVILRAVMEKCGDADDCISTSEAGELASSLDAKVIDGLPLPGQVDFI
NGGPPCQGFSGMNRFNQSTWSKVQCEMILAFLSFADYFRPKYFLLENVRNFVSFNKGQTF
RLTIASLLQMGYQVRFGILEAGAYGVSQSRKRAFIWAASPEEILPEWPEPMHVFAAPELKIT
LSEKSQYSAVRSTAYGAPFRAITVRDTIGDLPDVGNGASKTNLEYGNDPVSWFQKKIRGD
MVVLTDHISKEMNELNLIRCKKIPKRPGADWRDLPDEKVKLSTGQMVDLIPWCLPNTAK
RHNQWKGLFGRLDWEGNFPTSITDPQPMGKVGMCFHPEQDRILTVRECARSQGFPDSYQ

FSGNIHHKHRQIGNAVPPPLSYALGRKLKEALDSKRRK

>PtMET2
MGSSSILDTTTNDPVDNTISSSSSVGMRKNKKGKQKSSVSNVKKEVPEKNTKGKKRNSPD
TNKEEPTGGDGSLKRPKRAAACKDFKEKSVRLHEEKSYVVESKKEQVVDEEILAVRLTQG
QEEGRPNRRLIDFVVHDANGNPQPLEMVEVDDMFISGIIMPHEESLDKEKEVHVRCDGFG
RIEAWDISGYEDGSPVIWLSTEVADYDCIKPAGGYKKFFDHFFQKALACVEVYKKLSRFSG
GNPEFTLDELLAGVVRAMSGNKCFSGAVSIKNFLISQGEFIYHQIIGLDETSTKNDKKFADL
PVLVALRDESRNHGNVLIAKAANSGGNLVIGPESVDGAVVNQSNQSSTTVEEDEDAKLAR
LLQDEEYWQSNMRQKKSRGSVSASNTIYIKINEDEIANDYPLPAFYKHSNEETDEYIAVAS
DDVIDHPDDLPRRMLHNWSLYNSDSRLISLELLPMKPCEDIDVTIFGSGSMTEDDGSGFCL
DDGPDQSSSRGLEAQDDMGLPIFLSAIKEWMIEFGSSMIFISLRTDMAWYRLGKPSKQYAS
WYKPVLKTVKLARTIITLLKEQSRVSRLSFADVIRKVSEFKKDHHAYISSDLAAVERYVVV
HGQIILQLFAEFPDQKIKKCAFVVGLTRMMEERHHTKWVVNKKAIVQKCHSNLNPRAAM
DTVASGASKRKLMQATTTRLINRIWGEYYSNYSPEDLKEGNDCDVKEEDELEEQDENED
DDKEVVVEKTLKPYSVFEHCKSHTSQKEVRWDGNPVRKISSGEDIYKQAIVCGQVIVVGA
AVLVEVDEPDELPAIYFVEYMFETRNGSKMFHGRMMKWGSETVLGNTANDREVFLTNEC
MNYKLQDVKQTIILEVRKRPWGHHHRKDNANADRIDREKAEERKKKGLPLEYYCKSLY
WPERGAFFTLPFDTMGLGSGVCHSCNLKISEEDKNISKVNSSQTGFSYKGTEYSVHDFVY
VSPHQFAVESGETETFKGGRNVGLKPYAVCQLLEVVPMETKQSETRSTEVKVQRFFRPDDI
SPEKAYCSDIREIYYSEETHLLSVEVIEGKCEVRKKIDIPTCSAPAIFDHTFFCEHMYDPSNG
SLKQLPAHIKSKFSAVSKDGDVASRKRKGKSKEGENDTEDDKQLEASPEYRLATLDIFAGC
GGLSEGLQQAGVSTTKWAIEYEEPAGEAFKLNHAESLMFINNCNVILRAVMERCGDADD
CISTSEAAKMASSLDAKVIDGLPLPGQVDFINGGPPCQGFSGMNRFNQSTWSKVQCEMIL
AFLSFADYFRPKYFLLENVRNFVSFNKGQTFRLTIASLLQMGYQVRFGILEAGAYGVSQSR
KRAFIWAASPEEILPEWPEPMHVFAAPELKITLSEKSQYAAVRSTAYGAPFRAITVRDTIGD
LPDVANGASKTNLEYGNDPISWFQKKIRGDMVVLTDHISKEMNELNLIRCKNIPKRPGAD
WRDLPDEKVKLSTGQMVDLIPWCLPNTAKRHNQWKGLFGRLDWEGNFPTSITDPQPMG
KVGMCFHPEQDRILTVRECARSQGFPDNYQFFGNIQHKHRQIGNAVPPPLAYALGRKLKE
ALDSKRQK

>PtMET3
MNVGMRKNKKGKQKSSVSNVKKEVPEKNTKGKKRNSPDTNKEEPTGGDGSLKRPKRA
AACKDFKEKSVRLHEEKSYVVESKKEQVVDEEILAVRLTQGQEEGRPNRRLIDFVVHDAN
GNPQPLEMVEVDDMFISGIIMPHEESIDKEKEVHVRCDGFGRIEAWDISGYEDGSPVIWLS
TEVADYDCIKPAGGYKKFFDHFFQKALACVEVYKKLSRFSGGNPEFTLDELLAGVVRAM
SGNKCFSGAVSIKNFLISQGEFIYHQIIGLDETSTKNDKKFADLPVLVALRDESRNHGNVLIA
KAANSGGNLVIGPESVDGAVVNQSNQSSTTVEEDEDAKLARLLQEEEYWQSNMRQKKSR
GSVSASNTIYIKINEDEIANDYPLPVFYKHSNEETDEYIAVASDDVIDHPDDLPRRMLHNWS
LYNSDSRLISLELLPMKPCEDIDVTIFGSGSMTEDDGSGFCLDDGPDQSSSRGLEAQDDMG
LPIFLSAIKEWMIEFGSSMIFISLRTDMAWYRLGKPSKQYASWYKPVLKTVKLARSIITLLK
EQSRVSRLSFADVIRKVSEFKKDHHAYISSDLAAVERYVVVHGQIILQLFAEFPDQKIKKCA
FVVGLTRMMEERHHTKWVVNKKAIVQKCHSNLNPRAAMDTVASGTSKRKLMQATTTRL
INRIWGEYYSNYSPEDLKEGNDCDVKEEDELEEQDENEDDDKEVVVEKTLKPYSVFEHC

KSHTSQKEVRWDGNPVRKTSSGEDIYKQAIVCGQVIVVGAAVLVEVDEPDELPAIYFVEY
MFETRNGSKMFHGRMMKWGSETVLGNTANDREVFLTNECMNYKLQDVKQAIILEVRKR
PWGHHHRKDNANADRIDREKAEERKKKGLPLEYYCKSLYWPERGAFFTLPFDTMGLGS
GVCHSCNLKISEEDKNISKVNSSQTGFSYKGTEYSVHDFVYVNPHQFAVESGETETFKGGR
NVGLKPYAVCQLLEVVPMETKQSETRSTEVKVQRFFRPDDISPEKAYCSDIREIYYSEETHL
LSVEVIEGKCEVRKKIDIPTCSAPAIFDHTFFCEHMYDPSNGSLKQLPAHIKSKFSAVSKDG
DVASRKRKGKSKEGENDTEDDKQLEASPEYRLATLDIFAGCGGLSEGLQQAGVSTTKWAI
EYEEPAGEAFKLNHAESLMFINNCNVILRAVMERCGDADDCISTSEAAKLASSLDAKVIN
GLPLPGQVDFINGGPPCQGFSGMNRFNQSTWSKVQCEMILAFLSFADYFRPKYFLLENVR
NFVSFNKGQTFRLTIASLLQMGYQVRFGILEAGAYGVSQSRKRAFIWAASPEEILPEWPEP
MHVFAAPELKITLSEKSQYAAVRSTAYGAPFRAITVRDTIGDLPDVANGASKTNLEYGNDP
ISWFQKKIRGDMVVLTDHISKEMNELNLIRCKNIPKRPGADWRDLPDEKVKLSTGQMVD
LIPWCLPNTAKRHNQWKGLFGRLDWEGNFPTSITDPQPMGKVGMCFHPEQDRILTVREC
ARSQGFPDNYQFFGNIQHKHRQIGNAVPPPLAYALGRKLKEALDSKRQK

>PtDRM2
MEDPNRSRSSSDWNNNNKNLIKPKHEKLDFDLAFESLRSREVGDNVASSSRSYLRSFFTE
MGYSPSLVNRVIEENGEDNVDLLLEILMECSGLQKPNSQSSDSLDCLFDDRGESSPPKYST
VTGVKEEPDVFDEVYDDKRVSLLKMNFPAKEVELAMDKLGENAPINEIIDFIIAAQIANNL
DRETEDMPDIDAENKEDVNDETLYGTMDKTLCLLNMGFSENEVSLAIDKFGSEVPVTELA
NAICAHQLGETYVIKKKYSENSTASCSSAAEDSRSFGVETENNTRHHSFSWVKSETEDCR
RDAILQSRDMNTKETRKGKRPRQEHIEGYQEAQPRHDSLEENCAGEQPKQEYDYGSSSYF
EHEWVEEKVNSDTTTFGMPKPFKCNPCKILDQIAAKPPYFFYGNVATASSDTWGKISQFLY
GIEPEFVDTQFFSALSRREGYIHNLPTENRSHILPKPPITLEDLMPSTKKWWPSWDARKKM
SCRNFDSSGSSQLCDMLGRMLDDSRGLLSAEQQRDLLRHCQALNLMWVGPNKLSPLESA
HLEKILGYPLNHTLIADYPLTERMYSLRYSFQTDTLGYHLSVLKSIFPQGITVLSLFSGIGGA
EITLHRLGIHLKGVVSVETSETNRRVLKRWWYSSGQTGRLEQIEDIRKLTSSTVERLVENF
VCFDFVICQNSFTRPSKIPGVGSGLESQHFFDFTLFNEFVRVLQRVRSAIERKR

>PtDRM1
MDGDSLCGDNDDFDWDSEDEKEIENFASSSSSSLRLPQVETRSSSAEASSSVGSSSGSKMI
DHFVKMGFPEKMVAEAIQENCKGEEDEDSILETLLKYSTSSSASSSGSKLFDRFVGMGFAE
KMVAKAIKENGEGDADSVLETLLTYAAIGKSPQEQPNNDSDHCSSGHEGSFLDDFSDVDS
ADDEVITKTVSDEDNKLAFLRRMGYKEADASIAITRCGTEATISELADFICAAQIAKAEDA
FFAEDEKKPKHLDKQKKRSFLESDVLEKKRQKGLENGDDEGVRLPNPMVGFGVPTEPGI
VTRRTLSEAAIGPPFFYYENVALAPKGVWQTISRFLYDVEPEFVDSKHFCAAARKRGYVH
NLPIHNRFPLLPLPPNTIHEALPLTRKWWPAWDERTKLNCLQTCIASAKLTERIRKALEAYE
GEPPLHVQKFIMDECRKWNLVWVGRNKVAPLEADEVEMLLGFPRNHTRGGGISRTDRYK
SLGNSFQVDTVAYHLSSLKDLFPGGINVLSLFSGIGGAEVALHRLGIRLKNVVSVEISNVNR
SIMSCWWEQTNQTGNLIHIEDVQHLTADRLEQLMNMYGSFDLVVGGSPCNNLAGSNRHH
RDGLEGKESSLFFDYCRILDVVKNLTSRYS

>ZmMET1a

MLTNDFSGTRRCRAKPQKKEEESTENNKLENGSLDATEEVHHGVEKGDGHVTRKRPRRS
AACSDFKEKSIRLSEKKSVVMVKKNRMEEEEVDAVNLTKLGPEDPPPCRKLIDFILHDAEG
NPQPFEMSEIDDFFITALIMPMDDDLEKERERGVRCEGFGRIEDWNISGYDEGTPVIWVST
DVADYECVKPSTNYKSYFDHFYEKAQVCVEVFKKLAKSVGGNPNQGLDELLASVVRSTN
AMKGYSGTMSKDLVISIGEFVYNQLVGLDETSNNDDEKFATLPVLLSLRDQCRSRVELTKL
PSNFSNTSLKIKDSECDETAEDDDDAKLARLLQQEEEWKMMKKQRGRRGTPSQKNVYIK
ISEAEIANDYPLPAYYKPFSQEMDEYIFDSDDSIFSDDVPVRILNNWTLYNADSRLISLELIP
MKSGAENDVVIFGSGFMRDDDGSCCSTAESVKSSSSSSKADQLDAGIPIYLSPIKEWIIEFG
GSMICITIRTDVAWYKLRQPTKQYAPWCEPVLKTARLAVSIITLLKEQSRASKLSFADVIRK
VAEFDKGNPAFISSNITLVERYIVVHGQIILQQFADFPDETIRRSAFVSGLLLKMEQRRHTKL
VMKKKTQVMRGENLNPSAAMGPASRKKAMRATTTRLINRIWSDYYAHFPEDSKEGDGN
ETKEIDDEQEENEDEDAEDEGQIEENISKTPPSTRSRKLLSQTCKEIRWEGETSGKTLSGET
LYKCAYVRELRIPVGGTVALEDDSGDTVICFVEYMFQKVDGSKMVHGRILQKGSQTILGN
AANEREVFLTNDCLEFKLDDIKELVMVDIQSRPWGHKYRKENSEADKVEQVKAEERKKK
GQPMVYFCKSLYWPEKGAFFALSRDKMGLGSGLCSSCDNIEPDSDELKIFSKTSFVYRKV
TYNVNEFLYIRPDFFAEDEDRATFKAGRNVGLKPYAVCQILSIPEGAGSKKLNPASANISAR
RFYRPDDISSAKAYASDIREVYYSEDVIDVPVDMIEGKCEVRKKNDLASSDLPVMFEHVFF
CELIYDRASGALKQLPPNVRFMSMVQRTSALKKNKGKQICEPDQIDSGKWLDVPKENRL
ATLDIFAGCGGLSEGLQQAGVSFTKWAIEYEEPAGEAFNKNHPEAVVFVDNCNVILKAIM
DKCGDTDDCVSTSEAAEQAAKLPEVNINNLPVPGEVEFINGGPPCQGFSGMNRFNQSPWS
KVQCEMILAFLSFAEYFRPRFFLLENVRNFVSFNKGQTFRLAVASLLEMGYQVRFGILEAG
AFGVAQSRKRAFIWAAAPGEMLPDWPEPMHVFASPELKITLPDGQYYAAARSTAGGAPFR
AITVRDTIGDLPKVGNGASKLTLEYGGEPVSWFQKKIRGNMMVLNDHISKEMNELNLIRC
QHIPKRPGCDWHDLPDEKVKLSNGQMADLIPWCLPNTAKRHNQWKGLYGRLDWEGNFP
TSVTDPQPMGKVGMCFHPDQDRIITVRECARSQGFPDSYEFAGNIQNKHRQIGNAVPPPLA
YALGRKLKEAVDKRQEASAGVPAP*

>ZmMET1b

MLTNDFSGTRRCRAKPQKKEEESTENNKLENGPLDATEEVHHGVEKGDGHVTRKRPRRA
AACSDFKEKSIRLSEKKSVVMVKKNRMEEEEVDAVNLTKLGPEDPPPCRKLIDFILHDAEG
NPQPFEMSEIDDFFITALIMPMDDDLEKEREKGVRCEGFGRIEDWNISGYDEGTPVIWVST
DVADYECVKPSTNYKSYFDHFYEKAQVCVEVFKKLAKSVGGNPNQGLDELLASVVRSTN
AMKGYSGTMSKDLVISIGEFVYNQLVGLDETSNNDDEKFATLPVLLSLRDQCRSRVELTKL
PSNFSNTSLKIKDSECDETAEDDDDAKLARLLQQEEEWKMMKKQRGRRGTPSQKNVYIK
ISEAEIANDYPLPAYYKPFSQEMDEYIFDSDDSIFSDDVPVRILNNWTLYNADSRLISLELIP
MKSGAENDVVVFGSGFMRDDDGSCCSTAESVKSSSSSSKADQLDAGIPIYLSPIKEWIIEFG
GSMICITIRTDVAWYKLRQPTKQYAPWCEPVLKTARLAVSIITLLKEQSRASKLSFADVIRK
VAEFDKGNPAFISSNITLVERYIVVHGQIILQQFADFPDETIRRSAFVSGLLLKMEQRRHTKL
VMKKKTQVMRGENLNPSAAMGPASRKKAMRATTTRLINRIWSDYYAHHFPEDSKEGDG
NEIKEIDDEQEENEDEDAEDEGQIEENISKTPPSTRSRKLLSQTCKEIRWEGETSGKTLSGET
LYKCAYVRELRIPVGGTVALEDDSGDTVMCFVEYMFQKVDGSKMVHGRILQKGSQTILD
NAANEREVFLTNDCLEFKLDDIKELVMVDIQSRPWGHKYRKENSEADKVEQVKAEERKK

KGQPMVYFCKSLYWPEKGAFFALSRDKMGLGSGLCSSCDNIEPDSDELKIFSKTSFVYRK
VTYNVNEFLYISPDFFAEDEDRATFKAGRNVGLKPYAVCQILSIPEGAGSKKLNPASANISA
RRFYRPDDISSAKAYASDIREVYYSEDVIDVPVDMIEGKCEVRKKNDLASSDLPVMFEHVF
FCELIYDRASGALKQLPPNVRFMSMVQRTSALKKNKGKQICEPDQIDSGKWLDVPKENR
LATLDIFAGCGGLSEGLQQAGVSFTKWAIEYEEPAGEAFNKNHPEAVVFVDNCNVILKAIM
DKCGDTDDCVSTSEAAEQAAKLPEVNINNLPVPGEVEFINGGPPCQGFSGMNRFNQSPWS
KVQCEMILAFLSFAEYFRPRFFLLENVRNFVSFNKGQTFRLAVASLLEMGYQVRFGILEAG
AFGVAQSRKRAFIWAAAPGEMLPDWPEPMHVFASPELKITLPDGQYYAAARSTAGGAPFR
AITVRDTVGDLPKVGNGASKLTLEYGGEPVSWFQKKIRGNMMVLNDHISKEMNELNLIR
CQHIPKRPGCDWHDLPDEKVKLSNGQMADLIPWCLPNTAKRHNQWKGLYGRLDWEGNF
PTSVTDPQPMGKVGMCFHPDQDRIITVRECARSQGFPDSYEFAGNIQNKHRQIGNAVPPPL
AYALGRKLKEAVDKRQEASAGVPAP*
>ZmMET2a
MAPSSPSPAAPTRVSGRKRAAKAEEIHQNKEEEEEEEEVVAASSAKRSRKAASSGKKPKSP
PKQAKPGRKKKGDAEMKEPVEDDVCAEEPDEEELAMGEEEAEEQAMREEVVAVAAGSP
GKKRVGRRNAAAAAGDHEPEFIGSPVAADEARSNWPKRYGRSTAAKKPDEEEELKARCH
YRSAKVDNVVYCLGDDVYVKAGENEADYIGRITEFFEGTDQCHYFTCRWFFRAEDTVIN
SLVSISVDGHKHDPRRVFLSEEKNDNVLDCIISKVKIVHVDPNMDPKAKAQLIESCDLYYD
MSYSVAYSTFANISSENGQSDSDTASGISSDDVDLETSSSMPTRTATLLDLYSGCGGMSTGL
CLGAALSGLKLETRWAVDFNSFA
CQSLKYNHPQTEVRNEKADEFLALLKEWAVLCKKYVQDVDSNLASSEDQADEDSPLDKD
EFVVEKLVGICYGGSDRENGIYFKVQWEGYGPEEDTWEPIDNLSDCPQKIRDFVQEGHKR
KILPLPGDVDVICGGPPCQGISGFNRYRNRDEPLKDEKNKQMVTFMDIVAYLKPKYVLME
NVVDILKFADGYLGKYALSCLVAMKYQARLGMMVAGCYGLPQFRMRVFLWGALSSMVL
PKYPLPTYDVVVRGGAPNAFSQCMVAYDETQKPSLKKALLLGDAISDLPKVQNHQPNDV
MEYGGSPKTEFQRYIRLSRKDMLDWSFGEGAGPDEGKLLDHQPLRLNNDDYERVQQIPV
KKGANFRDLKGVRVGANNIVEWDPEIERVKLSSGKPLVPDYAMSFIKGKSLKPFGRLWW
DETVPTVVTRAEPHNQVIIHPTQARVLTIRENARLQGFPDYYRLFGPIKEKYIQVGNAVAVP
VARALGYCLGQAYLGESEGSDPLYQLPPSFTSVGGRTAGQARASPVGTPAGEVVEQ*

>ZmMET2b
MAPSSPSSARPTRASGRKRSAMAEEIHQNQEEEEEVVAASTAKRRRKAASSGKKPKPTPK
QAKPAVAGMKKKGETEKTEPVVDDVCAEEPDEEELAMGEEEAEAEEQAMQEVVAAVAA
GSPGKKRVGRRSAAASGDHVPEFIGSPVAAAEAHSNWPKRYERSTAANKPEEDDELKARC
HYRSAKVDNIVYCLGDDVYVKAGENEADYIGRITEFFEGTDRCHYFTCRWFFRAEDTVIN
SLVSINVDGHKHDPRRVFLSEEKNDNVLDCIISKVKIVHVDPNMDPKAKAQLIEHCDLYY
DMSYSVAYSTFANISSENGQSGSETASGISSDDAGLETSSNMPERTATLLDLYSGCGGMSTG
LCLGAALSGLKLETRWAVDLNSFACQSLKYNHPQTEVRNEKADEFLALLKEWAVLCEKY
VHQDVDSNLAGSEDQEDADTLDKDEFVVQKLIGIRYDGTGRKKGVYFKVQWEGYGPEE
DTWEPI
DNLSDCPLKIREFVQEGRKRKILPLPGDVDVICGGPPCQGISGFNRFRNRDEPLKDEKNKQ
MVTFMDIVAYLKPKYVLMENVVDILKFADGYLGKYALSCLVAMKYQARLGMMVAGCYG
LPQFRMRVFLWGALSSMVLPKYPLPTYDVVVRGGAPNAFSQCMVAYDETQRPSLKKALL
LGDAFSDLPKVENHQPNDVMEYGGSPKTEFQRYIRLGRKDMLDWSFGEEAGPDEGKLLD

HQPLRLNNDDYERVKQIPVKKGANFRDLKGVKVGANNVVEWDPEVERVYLSSGKPLVP
DYAMSFIKGKSLKPFGRLWWDETVPTVVTRAEPHNQVILHPTQARVLTIRENARLQGFPD
YYRLFGPIKEKYIQVGNAVAVPVARALGYCLGQAYLGESDGSQPLYQLPASFTSVGRTAVQ
ANAVSVGTPAGEVVEQ*
>ZmMET3a

MAHWVSDSDGSDNFEWDSDGNGEEPANFNAAGAGSSALRSTNTDAPGPSTRVANGNGK
AGPSASLVQKYMDMGFTEEIVRKAMKDNGDNGADSLVELLLTYQELGNDLKVDNGFAS
GCAPQTVDDSDDDDILENWDDEDADGGRSTRVANSIDDSEDEDFLNEMSQKDKKVDSLV
KMGFPEDEAALAITRCGQDASISVLVDSIYASQTAGDGYCGNLSDYEGNSNGGRNKGRF
MDGNKKKRKRYGGQAQGNRGPLDGSCEEPMPLPNPMVGFNLPDHWTRPVDRSLPTQAI
GPPYFYYENVALAPKGVWTTISRFLYDIQPEFVDSKYFCAAARKRGYIHNLPLERRSPLLP
LPPKTIFEAFPRTKRWWPSWDPRRQFNCLQTCTSSAKLLERIRVTLANSSDPPPPLVQKFVL
EECRKWNLAWVGLNKVAPLEPDEMEFLLGFPKDHTRGISRTERYRSLGNSFQVDTVAYHL
SVLKDRYPQGMNVLSLFTGIGGAEVALHRLGIRMNTVVSVEKSEVNRTILKSWWDQTQT
GTLIEINDVQTLTADRIEAYIRRIGGFDLVIGGSPCNNLAGSNRHHRDGLEGEHSSLFYHYF
RILDSVKSIMERL*
>ZmMET3b

MVHWVSDSDGSDNFEWDSDGNGEQTVSFNAAGAGSSALAATNTDAPGPSTRVANGNGK
AGRSASLVQKYVDMGFSEEIVLKAMKDNGDNGADSLVELLLTYQELGNDLKVDNDFASS
CAPKTADDSDDDDTLEIWDDEDAGGRSTRVANSVDDSDDEDFLHEMSRKDEKVDSLVK
MGFPEDEAALAITRCGPDASISVLVDSIYASQTAGDGYCGNLSDYEDNSYGGRSTGNKKK
RKRYGGQAQGSRGPLDGSCDEPMPLPHPMVGFNLPDQWSRRVDRSLPAQAIGPPYFYYE
NVALAPKGVWTTISRFLYDIQPEFVDSKYFCAAARKRGYIHNLPLENRSPLLPIPPKTISEAF
PRTKRWWPSWDPRRQFNCLQTCVSSAKLLERIRVALTNSSDPPPPRVQKYVLEECRKWNL
AWVGLNKVAPLEPDEMEFLLGFPKDHTRGISRTERYRSLGNSFQVDTVAYHLSVLKDLFP
QGMNVLSLFSGIGGAEVALHRLGIQMNTVISVEKSEVNRTILKSWWDQTQTGTLIEITDVQ
TLSSERIEAYIRRIGGFDLVIGGSPCNNLTGSNRHHRDGLEGEHSALFHHYFRILHAVKSIME
RL*
>ZmMET3c
MEKTLSLLQMGFTEEEVSSAIDNFDQRATVQELADSILARRIANSIEQKEIKVESDFLDEAE
TDYSSYQPSYSDVSYYDDDNN
NTRVKRAKHIFIDDIGASSSHLGDPWSMGQRAGTNDMSVKVELEAMTPGRRANVQGDL
AKPPFFLYGNVVEVPKDTWHQLKQFLYNVEPEFVNSQSFSALTRREGYIHNLPVEKRSVV
VPKSPMTIEEALPFTRQWWPSWDTRKHISVITTEAAGIEQTCERLGGMVRESRGVLSQAR
QMQIIHQCRVSNLIWVGRDRLGPLEPRQVEKILGYPYNHTNLFELNQPDRLAAMKYAFQT
DTLSYLLSVLKGKYQDGIRVLSIYSGIGGAEVALHRLGIPLKCVISVEESEVNRKILRRWWL
KTEQTGVLRQHAGIWKLKTHVIEDLVKEFGGFDLIIGGNYTSCKGGTTVNTTMGMDSNR
FYEYNRVVKRVRTAVGLS*
>ZmDNMT2
MEAPAPWRVLEFYSGIGGLRYSLMASGVRAEVVEAFDINDVANDVYEHNFGHRPCQGNI
QTLTASDLDKYKAHAWLLSPPCQP
YTRQGLQKHSADARAFSFIKILNLMQDMSYPPQMLFVENVVGFEVSDTHDQLLEVLSSLN

FNTQEFILSPLQFGVPYSRPRYFCLAKREPVRFRHAFVNN
KLLQTPMCLSLTLSSTSQGSDHQTEEELEPVCKPIKDFLVKEADGGTPDETVLQDYMVPV
NLIERWGNAMDIVYPEFKRCCCFTKSYYRYVKGTGSLLAT
SKNLNVPEEKLQISSLKELGLRFFTPREVANFHSFPSNFCFPDHISLRQQYAMLGNSLSIAVV
GPLLHYLFAEA*
>PhMET1
MGSLAELDKVDTDAAAGHKKDKSKQESVSKRKAPATNKKEKKRSVSETNEEPTAARKM
PK
RAAACTDFKEKSVHLSKNSSVIETKKDQCVEEEGVAIRLTAGQQDSQRPCRRLMDFVFHD
SKGKPQPFEMSEVDDLFISGLVLPLEDSFDKEKAKGIRCEGFGRIEEWAISGYEDGTPVI
WISTEAADYDCIKPSGSYKKFYDHFFAKATACIEVYKKLSKSSGGNPDLSLDELLAGVVR
AMSGLKCFSGGVSIRDFVVSQGEFIYKQLAGLDDTSKKTDQLFVELPVLASLRDESSKLE
MLAQPEPISSGKALCIGPKVGNTEDKTDQSGLTTGPGQEQEDLKLAKLLHEEEYWRSLKQ
KKGRSTSSSSSKFYIKINEDEIASDYPLPAYYKTSNEETDEYIVFDGGVDTYYIDDLPRS
MLHNWALYNSDSRLISLELLPMKPCADIDVTIFGSGVMTADDGSGYSFDADANHSSSGGS
GTAEIDGMPIYLSAIKEWMIEFGSSMVFISIRTDMAWYRLGKPSKQYAPWYEPVLKTARL
AVSIITLLKEQSRVARLSFGDIIKRVSEFNKGHPAYISSNADVVERYVVVHGQIILQQFS
EFPDESIRKCAFVLGLSRKMEERHHTKWLIKKKKVLQRNEQNLNPRASMAPSVKRKAMQ
A
TTTRLINRIWGEYYSNYSPEESKEVVGCQVKDDDEAEDQEENDEDDTTDENLDVPEKNTP
CSTRRHIKSSSDSKEINWDGESIGKTGSGELLFRKARVHGNEIAVGDSVLVEVDEPDELP
SIYFVEYMFEKLDGSKMLHGRMMQRGSETVLGNAANEREVFLINECVDLQLKDVKETV
VV
NIRMLPWGHQHRKANADADKLDRARAEDRKRKELPTEFYCKSFYQPERGAFFRLSFDK
MG
LGNGLCYSCELQQTDREKETFKLDISSSSFVYLGTEYSVDDFVYVSPDYFATERGGSETY
KAGRNVGLMAYVVCQLLGIVGSKGHKQAKVDSTDVKVRRFFRPEDISSDKAYSSDIREIY
YSEEIHTVPLETIKGKCEVRKKYDIPSEDVPAIFDHVFFCEYMYDPSNGSLKQLPAQVKL
RYSEIKLDDAASRKRKGKGKEGEDELRVGEQNRASQQKQLATLDIFAGCGGLSEGLQHSG
ITDTKWAIEYEEPAGDAFRLNHPKAKVFIHNCNVILRAVMQKSGDADDCISTPEASDLAA
KMDENELNSLPLPGQVDFINGGPPCQGFSGMNRFNQSTWSKVQCEMILAFLSFADYYRPK
FFLLENVRNFVSFNQKQTFRLTVASLLEMGYQVRFGILEAGAFGVPQSRKRAFIWAASPE
EVLPEWPEPMHVFAVPELKISLSGTSHYAAVRSTASGAPFRSLTVRDTIGDLPAVGNGAS
KTCIEYQGDPVSWFQKKIRGNSIALSDHISKEMNELNLIRCQRIPKRPGSDWRDLPEEKV
KLSNGQLVDLIPWCLPNTAKRHNQWKGLFGRLDWDGNFPTSITDPQPMGKVGMCFHPD
QD
RIVTVRECARSQGFPDSYQFSGNILHKHRQIGNAVPPPLAYALGRKLKEAVESKRST
>PhCMT2
MATTEEENKNNSSKKSPESALAPSFPRNEEPVPLEVFYPDDRTPIIRRRSSTFTTNIFTS
DILPKFTIPKQLLVPALSQNGKIVNSGKSEGDFSGKRKLTPCLDDEMQIQKSPRSNHIPN
EFDGSASVSRSAGKSGMMRGLAALMTTPDVNNKSASSAPASKGGGSSNGAGPELLALPG
P
EESASVIRSASKRGTTRELLALLTSESNNSSASSTRASKGSNSRGSPIILDEILGESNLS
GEKSLRSRKININNNNNNNSNNSIKKINGSNIKRKNNNNSSEEINSSNERKQKKSSNVCFI

GEPIPAEEAQEKWQWRYDLKALAKHGIICMEDLVHEIMTVGSHFKEANNFLWPFQLKVTF
GWTQEEEESLRGRHDGEEDEIILNVECHYAQAKVAGFIFSIGDCAYVKGEGRKKHIGRIL
EFFKTTEGEDYFRVLKGAASFHDPKRLFYSVLENDNLLDCIVSKVNVVELPTRHGMNTKD
IPPAHFYYDMEYCVDYSTFRTLHNVKSSVFPSLVDTSYKPVTTYPLEVLPSCEPMKVELS
LLDLYAGCGGMSTGLSLGTKLSGLKLVTKWAVDFNGSACDSLKLNHPQTHVRNEAVEDF
L
ELLKRWEKLIQSYGCSDFKTSSNCELDDIEEGESNDNSQAGSKGSSGEYEGDVGVICGGP
PCQGISGYNRHRNVDDPLADEKNRQIIIFMDVVEFLRPKYVLMENVADILRFDKASLGRY
ALSRLVHMRYQARLGTMAAGCYGLPQFRLRVFFWGALPSERNTVAYEEGQARGDLEEAL
V
LRDAISDLPAVTWHEVREEMPYEMPPESEFQKYIRLPKHEIMGCSSTGVTKTEVPVLFDH
RPCQLPEDDYLRVCLVPRRKGANFRDLPGVIVGQDNVARRDTEEPKHLPNGKPMVPDCA
F
NFEHGKSKRPFARLWWDETVATLVTFPNHRAQAILHPEQDRVLTIREYARLQGFPDFYKF
SGTLKERYCQVGNAVAVPVGRALGYALGLAFQRLTGNEPLIKLPPNFSFLKPPIDDIVVL
QN
>PhCMT3
MTRKRRASLESNPESSSATRKSKRLAVVPKPDPVPADSDFESEPDSSKNTKSIRRSYNKK
LKQQAAVKVESVVASSVNTDFVSESDSTIKAEEKVDFAVADSDFVDEEDELELGNLKKNL
SASPSKRKPMRSERANDEEYLFIGEPVPGDEAQQKWPNRYVTKNKGKANFTKNLNAQDD
S
DQCIQAKCHFTQAKVDGQIYNLGDDAHVQVVEFFEAVDGLQYFTAQWFYRAKDTVIKC
HD
KYIDKKRVFLSEIKDDNPIDCLVTKLKIVPATSQFKENIKSNCDYYYDMTYLLPYSSFMS
LPPDATSPVSSESTISSDIEAAEVKEHKVEKKLLDLYSGCGAMSTGLCLGANSNGVNLVT
KWAVDLSPFACNSLKLNHPETQVRNEFADDFLELLKHWVQLCVSCSLVKNGVAPHPDLEI
KDEVEEDEDDDESEDSGDDKGEVFEVEELLEVCYGDPKEVKKPGVHFKVRWKGYGPEE
DT
WEPIKGLSGCPKKIKEFVTKGFNSNILPLPGDVDVICGGPPCQGISGFNRFRNKDNPLDD
PKNKQLDVFMDIVDFLKPKFVLMENVVDLIKFSNGYLGRYALGRLLGMNYQARMGMM
AAG
AYGLPQFRMRVFVWGALPSEKLPQYPLPTHKVIKRGVIPVEFESHTVAYDEGLELDLKEA
LFLGDALSDLPSVENDERRDEMPYTDEPKTDFQHFIRLGRDGTLGSVLYDHRPLQLNEDD
YQRVCQIPKRKGANFRDLPGVRVKTDNKVEWDPDVERVKLPSGKPLVPDYAMTFVGGTS
S
KPFSRLWWDETVPTVVTRAEPHNQTILHPLQDRVLTIRENARLQGFPDYYKLTGPIKERY
IQVGNAVAVPVARALGYSLAMSIKGLSEEKPLYSLPKNFPSHVELTYNEVSQ
>PhCMT1
MPRKRKASTNSNCASASTTRKSKRHAKEKPDPVAVAESAECEFIEDEETVSNNEAFVEKR
VKRVTQVKDNEECKFSGDPLPDTEAHQRWPHRYIYKDKVRESSAPTSLNGQDDPDQLIQA
KCHYTQALVDNQFYNLGDDAYVQAAENEDDYICKIVEFFQAVDGMLYFTAQWFYRAKD
TV
IKTHDQFIDKKRVFFSEIKDDNLLDCLVKKIKIVPVPSNTGLQLKESLISDSDYYYDMMY
LLPYSSFVGLPADVSSPISESGSTISSDGEDVEVKEKKLLDLYSGCGAMSTGLCLGADIF

NVRNESAEDFLLLLQEWEQLCVSCSLLKSNVPPHPSLNVMNEDEEDNDDDTGDEDEGSG
H
DEDGVFEVEELLQICYGDPKEIKKPGLYFKVRWKGYGPDDDTWEPVDGLDGCGSKIKDF
V
TRGFKANMLPLPGEVDVICGGPPCQGISGFNRFRNKANPLQDPKNKQLEVFMNIVKFLRP
RFVLMENVVDLVRFSNGFLGRYALGRLVGMNYQARMGMMIAGAYGLPQFRMRVFMWG
ALP
SEKLPQYPLPTHNVVSRGGIPTEFELNAVHDESLKAKLKRELFLKDALSDLPPVWTLGTV
LYDHRPLQLNEDDYERVCQIPKRKGANFRVLPGVRVGANNVVEWDPDVERVKLASGKPL
V
PDYAMKFVRGTSPKPFGRLWWDEIVATVVTRAEPHNQVILHPEQDRVLTIRENARLQGFP
DYYKLIGPIKERYIQVGNAVAVPVARALGYSLALALKGLSGGNPLMSLPPNFPCLDELGS
NEVSED
>PhDNMT2
MESESCRNQPWRVLEFYSGIGGMRYSLLKAAVNATVIEAFDINDVANDVYQHNFGHRPFQ
GNIQTLNAADIDGYNADAWLLSPPCQPYTRQGLQKGSNDARASSFLKILELIPQLLRPPS
YLFVENVVGFETSDTHDILVEILEKNNFVTQEFILSPLQFGLPYSRPRYYCLAKRKPLSF
EVPEFNNQLLRTPGPLLGQTESTMEKQQLQSSEYWDELLQACKPIDDFLVFKPFGNEVDS
STYSFHVNDSAKSDRTDVDNNMYCVPSSLVERWGSAMDIVYPHSKRCCCFTKSYYRYVK
G
TGSLLATVQEKSEGRTPSLQDLCLRYFTPREWDYTGYCCCCCIKSRHLMILIVGCKFAFF
SRRLPVSTAHQSSPTGTFGPPPPTFFPPPSHRYNTLT
>PhDRM4
MQDKNLSGEENDNIDWDTEDELEIQEIQDTPFSACMNLRTTGQHAVNGDGEASSSSVPSQ
SKFIQQFVVMGFPEKSIAKAIQQNGENSDLVLDALLTFKALEDSPVEQLSVSPQQESCIS
SDDSSSEYENFLDDVDEEDSWSSDSDGCISSQRCYLKGDGSSLSQKERTLFSLANMGYLD
EEASIAMERCGPEASLAELTDFISAAQMARAEDPYLPEDIKPKMKHISSGYKKRKMYNEL
CKRKTKMTGFDEETVRLPKHMIGFGVPMEPSPISLQRTLPEQAIGPPFFYYENIALAPKG
VWDTISRSLYDIQPEFVDSKYFCAAARKRGYIHNLPIENRFPLLPLAPQTIHEVLPLTKK
WWPSWDTRTKLNCLQTSIASAQLTDRIRKVLEDPRLDGEQVQKCVLDKCRQWNLVWVG
RN
KVAPLEPDEVEMLLGFPKNHTRGISRTDRYKSLGNSFQVDTVAYHLSVLKDLFPKGINVL
SLFSGIGGAEVALHRLGIRLKNVVSVEKYEVNRNIVRSWWEQTNQMGNLIEFDDVQQLN
G
DRLEQLIESCGGFDLVIGGSPCNNLSGGNRVSRDGLEGTESILFFHFPRILDLVKSIMSS
HG
>PhDRM1
DKNLSEEESDSIDWDTESEPEVQEIQDTAFSTCMNLRTTGHHAFSGDAEASSSSGSCWSK
FIQQFQEMGFPKESIAKAIEQNGDNFDLVLNALLTFKALEETPDEQPSASPQLESCLSSD
DSSSEYYENFLDDVYEEDSWSSDSDDCKLSEKEQKLLSLRNMGYSEEDASIAIERCGPEA
LLGILIDFISAAEWAREEDRYLPEDIKPKVKHIPGGHKKRRMYNELCKRKKQRAIAKEET
IRLPKPMIRFRVPMESPLVVQRALPEQAIVFLRCKEKGYIHNLPVRNRFPLLPLPPLTIH
EALPSTKKYWPSWDTRTRLNCLQTSIGSAKLTNRIRKVLEDPRFDGEPPKEVQIGVLEKC
RQWNLVWIGRNKLAPLEPDEYELLLGYPKNHTRGISRTDRYKSLGNSFQVDTVAYHLSVL

KDLFPEGINVLSLFSGIGGAEVALHRLEIRLKNVVSVEISEVNRDVVRSWWEQTNQRGNL
IHIKDVEELNGDSLEQLIESYGGFDLVIGGSPCNNLAGSNRVSREGLVGTESILFFHYPR
ILALVKTIMSRHR
>PhDRM2
MDNNLSGEENEDIDWDTEDELEIQEIQDTTVPSCMNLRTTVPAVSSDWEASSSSVPFQSK
FIQQFIVMGFPEESIAKAIEQNGENSELVLDTLLTFKALEDSPEEQPSASPQLEPCISSD
DSSSEYYENLLDDVYEEDSWSSDSDHLINSATQCYLRDDSNSLSPKDQTLLSLVNMGYPR
EEASIAMERCPGASLPELADFMYAAQMARAEDPYLPEDIKPKLNHISGGYKKRKMYSELC
KRKKRAIFDEETIRLPKPMIGFGVPTESFPAVVQRTLPEQAKGPPFFYYENVALAPKGVW
DTISRNLYGIEPEFVDSKYFCAAARKRGYIHNLPIENRFPLLPLPPRTINEALPLTKKWW
PSWDSRTKLNCLQTAYGSARLTDRIRKAVEDFDGEPPERVQKYVLEQCRKWNLVWVGRN
K
VAPLEPDEVEMLLGFPKNHTRGGGISRTDRYKSLGNSFQVDTVAYHLSVLKDLFPNGINV
LSLFSGIGGAEVALYRLGIRLNNVVSVEKSEVNRNIVRSWWEQTNQRGNLIDFDDVQQLN
GDRLEQLIDSFGGFDLIIGGSPCNNLAGSNRVSRDGLEGQESSLFYHYPRILELVKDIMA
RYRM
>PhDRM3
DNIASSSGSNVRSSLLSMGFKAPLVDKAIKENGEDNIDLLLETLFANSDLHRAESSDSLD
SLFSDDEDTKGPAKCDGDVHIKEEPDPCVGVRDEKRASLLAMSFSLDEVEFAIGKLGEAA
PINELVDVIFAARIAANYKKDDDDDCVEIKDMKKECSTEALFGTMEKTLKLLEMGFSENE
VSAVIDKYGSEVPLEELANLIVDPNSSRRRDKHLLKSLSMNDSIGFNPLAVKKEEPCLDT
FESKDLNLLEKFKGKRPMSLADYIDERGTSKRPKPEYDEGFSSSLGPAWQEIINSNLRPS
NTTRTPVSRRTHQRTRVLDCQGTQKLSMPNSCRSLDQMVAKAPYFFYGNVMNLSQDSW
VK
ISQFLYAIEPEFVNSQHFSALSRKEGYLHNLPTENRFHIVPKSPMTIQEAVPNSGKWWPS
WDTRKHLSCINSEISVISQLCERLERSMSESQGFPSVDKQRDILHQCQLFNLAWVGRYKL
APVGPEQIERILGYPENHTRLAGFSLMERLLCLKHCFQIDTLAYHLSALKSLYPGGLTVL
SIFSGIGGAEIALHRLGIRLKAVVSIEASEKNRTILKRWWGSSGQTGELVQMEDVSKMAG
SKIEVLLKIYGGFDFIICQSPCTYSSKGNLAADNNSPASLDFMLFHEFVRVLQRVRSTVG
RN
>AtDnmt2
MAEQELQRINEKKPWQVLEFYSGIGGMRYSLMASGIVSEVVEAFEINDSANDVYQHNFK
HRPYQGNIQSLTAADLDKYNADAWLLSPPCQPYTRQGLQKHSGDARAFSFLRILELIPHTT
KPPQMLFVENVVGFETSDTHMEMIGTLTKLDYVTQEFILSPLQFGVPYSRPRYFCLAKRKP
LPFKSQHSNNKLLWSPDPLYGRDDQVEFGKCQAEEGLDKLLEFCKPVEKFLELAAHVDG
EPSSVDDSENGSKDCCGQEGDSVPDSVHQYLVPVSLIERWGNAMDIVYPDSKRCCCFTKS
YYRYVKGTGSLLATVQPKIKGKESCLKEQRLRYFTPREVANFHSFPEDFEFPKHISLRQRYA
MLGNSLSVAVVAPLLRYLFDS
>CcDnmt2_like1
MEEEGGLPAKQPWRVLEFYSGIGGMRYSVMKAGLQTEMVEAFDINDLANDVYEHNFGH
RPFQGNIQTLTAADLDRYCANVWLLSPPCQPYTRQGLQKQSADARASSFLRILEIIPQLML
PPVMLFVENVVGFETSDTHQKMVQILEESQFEMQEFILSPLQFGVPYSRPRYFCLAKRKPS
SFCNPEFNRQLLHVPELLVGCNESISISENGQLTSTSDKMLQTCLPIERFLEFGNSMNQVEN
GTSFPPDTCSDESSLKQYFVPSNLIERWGSAMGILDIKFGIDIVYPDSKRCCCFTKSYYRYV

KGTGSLLTTVMPKTRDKTSLEELCLRYFTPREVSNLHSFPKEFEFPEHVTLRQRYALPEKCT
EFGVRIVSHMYEIINTHGI
>CcDnmt2_like2
MEEEGLPAKQPWRVLEFYSGIGGMRYSVMKAGLQTEMVEAFDINDLANDVYEHNFGHR
PFQGNIQTLTAADLDRYRANVWLLSPPCQPYTRQGLQKQSADARASSFLRILEIIPQLMLPP
VMLFVENVVGFETSDTHQKMVQILEESQFEMQEFILSPLQFGVPYSRPRYFCLAKRKPSSF
CNPEFNGQLLHVPELLFGCNESISISENGQLTSTSDKMXQTCLPIERFLEFGNSMNQVENGT
SFPPNTFSDENSLKQYFVPSNLIERWGSAMGILDIKLFISSILIQSAAVASQRPKTRDKTSLEE
LCLRYFTPREVSNLHSFPKEFEFPEHVTLRQRYALLGNSLSAAVVAPLLHYLFSEP
>SlDnmt2
MESEVSRSPWRVLEFYSGIGGLRYSLLKAAVDATVVEAFDINDVANDVYQHNFGHRPFQG
NIQTLNAADLDGYNADAWLLSPPCQPYTRQGLQKGSSDARASSFLKILELVPQLLRPPSYL
FVENVVGFETSDTHAILVDILEKNNFVTQEFILSPLQFGLPYSRPRYYCLAKRKPLSFEVPEF
NNQLLRTPGPLLGQTESTMEKEQLLSPEYWDELLQACHPVDDFLVFKTFGNRKDSSTYSF
HANDSVKSDRLDEENDVCFVPSSLIERWGSAMDIVYPHSKRCCCFTKSYYRYVKGTGSLL
ATAEATFQEKPENRMPSLQDLSLRYFTPREVANLHSFPEDFQFPQHISLRQRYAMLGNSLSV
GVVAPLLQYLFINHS*
