# Supplementary material for: Exploration of the Potential Transcriptional Regulatory Mechanisms of DNA Methyltransferases and MBD Genes in Petunia Anther Development and Multi-Stress Responses
Source: Genes (Basel). 2022 Feb 8;13(2):314. doi: 10.3390/genes13020314 (PMC8872020; doi:10.3390/genes13020314)
Supplement: Supplementary file 1 [file genes-13-00314-s001.zip › Table S2.pdf]

Table S2. Sequences of MBD proteins in the 7 tested species.

>PhMBD1
MVKDTPKTPKTFKPAANSSVSIWAAQCGNCFKWRMISTQEEFEEIRSRFIEEPFYCNNKPN
VSCEDPADIEYDSSRTWAIDKPNQPKTPNGFKRELCLRKDYSKMDAYYTTPTGKRLRSSTE
VATFLQQNPEFSDLSVSDFTFKSPKIMEDTIPSSVLANSNKKSAK

>PhMBD2
MEGDHPDTVTPGKMDQNNNHGSDPANLNNVNTEPHALRVSSPDIMETPTQKPAEVTSPA
REQANQISEDKENHNKNAQSQLVLYDPAVSGNIEPFRARRKSFPNSTSRVLPSVGAFTVQC
ANCFKWRLIPTKEKYEEIRGHILEHPFYCETAREWRPEVSCDDPPDITQDRTRLWAIDKPNI
PHPPSGWERILRIRAEGGTKFADVYYVSPSGKRMRSMVEVQKHLDEHPEYVAQGVQLNQ
FSFLSPRPLQEDYVKKRRPAPSRDVPSDVTPISWVAPDGSTSSQLLGAPGPSGPSDTAPPDV
PVEKSPKKPKLMDDNDSVAK

>PhMBD3
MNVCYSDPSKGLKFFSKADVLRYLSSVGSNCSGSSNTKEMKKKDTGKFSTEKTSEGLPPG
WTKEIRVRRKGHKIRKDPYYIDHVSGQSFRSMKEVYRFLETRESGRFESKPGDQCPSTSEL
GESISSITTDREASGKVVNSNQKLKLGAEHMVHSSCLEGSVSSFEGGAVTENAREEDDGK
VASVSVSGEHQEKVDDGKVVSESARGELQEKEDDCKVGSDPVKGENQGKEDDCNIVSDR
VSGEDQEKPSRDGVEKHDQKTIQRKRKKGMNLPRRMSKRLAGVKADTSLELQTNNQAY
LATVRQEEETEAASTDNPVKFIKTSRHVATAVAGITISENVEAGSQTSKRQQDLVSLPLKER
ELPSPAEPAKTGLASCEADKKGETALESSLSDIWTDPCIEFAIKTLTGTIPLINEKKVDSTPGP
CLGMPSVNTPPSVGLPSDDIWADPCFMFAVKTLTGEIPIGDDFCSQKIVQQECISSQFQGNN
GLALANMRFQEFGQSNLSQFGVPEKAMYKKQPAVAPNTYQNSWFTKFGSHQSSPLC

>PhMBD4
MLLSLVSLVDRNPTTPKIPSQLRFRRRWSSEIHRPIGLLRALKKISKQLTIYTHVETGKKFYS
KKEVSRYMNSKDTCLGVTQVDYQDNKCSEDKVSEMNYQEKSFSDNNVSQVNDQDKSCF
EDNVSQMNNQDKSSSGNNVSQIVEGTKISHEWLPPGWIVELRTRKSGSNAGMTYKVYIDP
STGSKFYSKPEVSKYLKTMKQNNIAGEEQIHGTAEVSSSNQKASDNSKDIGGNSRISKRRK
SDTDQSSNGIEEGSKQKTSQDSEENGERSCISKRKKSGANRPSTKTFPAHGAGDDFSNEKA
SQDSKENGPHSPLSKRKKPGTDSPSAKTLYTDPVHGYVFRSKKDVFRYLETGDTSRCARR
SAKSNVASTPTTDDASLKKSECSVTGKQLFATDEPKAGKSLVTCSPAQVEKSKKQPECSVA
DANTIITSILAEINENHFDENINEDAEIGLAGATESVGLKRLSPVSTPQSDLRPAKQLPENEQ
EKRSCKAATEQSSKSRNTKSLKPGVRVSKRLAGHSPEVEADLDLGERALPAVVEKSASPSP
PLDVGGQELLKQPVPKLETGDSAQASPSSAAPSDDLPPGWKKEIKITKKASGIRKDPLYID
PVHGYVFRSKKDVFRYLQTGDINSCATRPGKKDLDTTLKESSPTTLNTSSKNSGCSVTERQ
PFASRGGKRSGTYSPVVQAKSSKKQPGSSASNPNTIITSSAADINAKHSYENGIENAEISLD
TEPEIRDTNGDRKVIVSESVDVKRVSAVSARKSDVLTKEKLPENEQEKQTNEETVEQSKRS
RKKKLLTPGHRTSKRLVGQNPEPVADLDIGERALRAVVKKSTSSGLPLKVNSQEIPAQNKY
PTVGTGNSDQASLSRETSVAEIPPGREKEILAQNKDPTVGTGNSDQAYLSRDAPVDKISSLS
RDPPVGKISPRRKKKTPAQNKDPAVPAQNKYPTEGTGNSDQASLSRETSVAEIPPGREKEIL
ALNKDPTVGTGNSDQASLSRDAPVDKVSSLSRDPPVGKISPRRKKKTPAQNKDPAVPAQN

KYPTVGTGNSDQASLSRGTSVAEIPPGREKEILALNKDPTVGTGNSDQASLSRDAPVDKVS
SLSRDPPVGKILPRRKRKTPAQNKDPAVGTGISDQASQSRVASEDELPPGWTKEILVRNGDP
TVRTGNAGQASLSRELSDDELPPGWTREMLVQNRDPTVRTGNAEQVSLSREFSDDELPPG
WTREMLAHKKDPIVRTGNIEQASLSREYSDDELPPGWTREMLAEKKDPIVRTGNIEQASL
SREYSDDELPPGWTREMLAQNKDSTRGNGNSHQASLSGEASVDELPPGWTKEMLAQNK
APTKGTGSSGQASVDELPPGWKKEIRIRKNSSRIKKDPFNLALQFYTDPVHGYIFRSRRDV
MRYLQTGDISSCAVRPTKRGPDSAVEDNSVSLHLSGSHHLRSRIKPTVATCLISPVSYLIAIQ
LAYGGNCSITYSMAPQAESSNEQPGSSGFNLNTSISSIAAEITETRSCENVNKDTEVRLDIE
AEIRDLFGEPKTIASEYVDVKRSSAVFPQSDILPEQKLPETEKEKHSNKGALAQSRKFRNK
NSLAPVRRVSKKFSHHNPDVAANLDLGEHALRAVVKKTAPSGFPLNVSGQGFPQQIDLVT
GSFDPAYLSKKASSQDDPLGEFTKCSKESQAFKEQKAAMELTSEKQDDEKRILQDSWPAEI
LHGSRNVQLERQEVREWSAAELVKEEQGSQKRFLKDWQPEELAWESRRLPVEDQSASER
STAPQVHKRRGNENMQWQDPQLAQPSQRRGELNVENQSVRGKQTGELVTENQDDQSRR
LQTQLSSHPFGDYWSDPCLEFAFKTLTGAIPVEDTLPFQGSTQQEFGSYPQVYDGCFDLPV
FNNYSFDMNEVPNPCPSEEHLPINQTFLPTGNNSIPGSSSVSSQNPGLDTQAKDYQSNFKP
RR

>PhMBD5
MAETAVSSNYPDSLNITGDFPDDCLPPDPLLQSGTYIIDGGDSNVNVDITPEKDLVDERHVS
DLAPPAEKGESTAEMQTAVTETVTPQRRVNKSVETTAERPSWLPENWKIEMRVRTSGATA
GTVDRYYFEPVSGRKFRSKIEVLYFLETGSKRKKGSTDSEATPSETPKSQKQKKGGSKTKK
ITSFYFDSRNPPQSVCWVHTDSFADTWTASCHGVMVPERRKDEWDAVFSSVSQLRQRKE
CINF

>PhMBD6
MEQDQLGLATPGKTDHNNIHGSSVASHRDNTKHEPNNLRDPASSSVIGTPNQTPIDLTSSPE
REDHSTEDEDNHGEDSQKQLVLYDPAAVGAGDIELLPDPVSSQPSRRNSFPNNTSRILPAVG
AFTVQCANCFKWRLIPTKEKYEEIREHILEEPFYCETAREWRAGVSCDDPPDLTQDGSRLW
AIDKPNIAQPPAGWERLLRIRGEGGTRYYVAPSGKRLRSMIEIQKYLQEHPDYVRQGVSMS
QFSFQIPRPLQENYVKKRPYRPALAHGENDPVHPIAWVGPADDTDLRLGMPGMSSPSDKA
PLFEPVSQSLKKKRTPSKRMRNADAASN

>PhMBD7
MDKRNSSTMMIPMPPPTRRHRSGAEYSTNNRQLMVVPPPTSTAITPYTTTSLSSFKLPPGW
GVQEVPRPHGSRVDKYYYEPGTGLKFRSLREVERRLNGEIFASRSRSSLSRKMVVYGGKV
VRMDDEQLNHWAIVPSTSAANLPYDLPDGWVIEEVPRRHKSFVDKFYYEPGTGQKFRSRI
AAQRYLAEMREDAPLSATLEELKENKPLSKMFKLHHHVK

>PhMBD8
MASSNMEKATHDDEIVSFTPKQGGTPKKNEVTFIAPTGEEVKSRKQLEQYLKSHPGNPAIS
EFDWSTGETPRRSARISEKVKAKRPPSLLESPQKRRRTSSGAKKENKETDAAKVEKETSDT
KEVESAKEENENLEKKDGEAEKVDDADDGKLDKSEDAKTEEKQASGEKLEPQNDQESS
AADVNENKPEGMTAEYAADEEFKDAHENKPEAVTGEHAVDEEFKDAQENKPEEDQAVD
EEFKDAQENHPEAATAEHAVDEEFKDAHGDDGESSLGEKKEAEGTDMVMENNKINQPG

MAHSQQQHQSPAPISC

>PhMBD9
MEKTEVLPLELPAPPSWKKLLMPKKGGKIKKNEVVFVAPTGEEIRKRKELEKYLKAHDG
NPGISEFDWSTGESLRRSARITEKIKFMPPPAVLEQTKKRRRTSSATKKDKEIDFDEGEKESL
DKREMESGKEESEGSGKKENVGKNVMQDEGKDELGDQKEDTEHKKEMEVSDREVSGR
MNEISDYTQVDAGKIAENGAHETMAIDTKVQNDSTFKDFFNGLVADAELEKTNAAEAGA
EVKEQQIDKGSSADDVNHDIPDNITPETIVAVEDINNIQEPAFIGETNDFHENLGEHRSLEEE
KDKNSRASLMMDNGKINQPEPAHTPQHQSAAPISC

>PhMBD10
MASSEVVSIELPAPPGWKKTFLPKEGGTPKKNEIIFTAPTGEEITTKKQLEQYLKSHPGGPP
VAEFDWGTGETPRRSARISGKAKAAPSPAESESAKKRSRKSSASKKDAKANEDPEETEPA
KDDDMEEAEKHEDTVAMEAEKDVEHKQVENQDGETEVEKKEEAQATEKDLQKKDEIQS
ENDVVKENPDEKKDEGQNADGKVEDVPSDEAQDEKDVKMADNVGHPEDVEVAPVDKA
LDADSKVEDVPVAEAEVEKDVKIADNVGHPEDVEEALVDKAADGPEATKMNEEIDVQV
QEKEENIPTEAEKVDSSADEGKKHQVVGEQKDEQKTSAATGSIEDQDNVMNSEISKVEGG
VTENGSNGNEAKP

>PhMBD11
MASVTLDPSSTITEGPPPPALQAESIPTVDLNLLSQSELYSLSLCSPSAFNPRLRHDDVIIPKI
DRSVFNESAGSRKQTYSRLRLAPASSAPGPGPRSRTPHLRSNFPQTSFQNNTNDPENTQIVS
LLKQLFGSGQMNPSNLIPVRVDYSNFVPTPVPELDNVGGVKRKRGPGRPRKGEDVVMKD
IVVYQNVVDDDRDKDKDKEIVNKDGEQVDLTGLGAMENPFGEELRKRTEGLVSAEELLG
FLERLNGEWGSTRRKKRIVDASEFGNVLPKGWKLLISVKKKEGRVWLHCRRYISPNGRRF
DTCKEVSSYLLSLHGERNENVPAYAKSSETVEITNTCALVSTSDLRVQDGAEKESAPSHNS
SPAVGPGELQVLLNFGELSEVQVGDLLQCDKCNVTFNNKDDLLQHQLSSHRRKRSRNGQ
SITDGVIIKDGKFECQFCHKTFEERRRYNGHVGNHVKYQGKTAADGSLSINVGKCVEPVV
SSGAMLTEVIVQDSVASARDMTENVDVRADVGNNPAPPSKIEEDHMETDDKLEGRCEAM
DTVPNKTNLCLSSEVVTLNNKDNNCCGTLADSLIVDNVTEGTNKDFHNQEGSSESCFPISL
TEKTCTNKSNVIVCSIIEEPKQESLLCSNGIVDSCGVSMEDGKFPPTLDESKVVTDRFVDNE
STNALCSNLGVLDENTLVSAEQVPCAEDYSGKNIDLLNGVSFLAETSRDTRGLKSSTGTPN
CEEEGSTVDHVQGVLSGSIGEQKPNSTCDITNDECGSLVDPNDNKLTMKEDSCSFVLPPVE
HVNIADRDVSDVSVCFLEEPERRIGDKSGLPSPARENSSGVEAQFFDGSKTTSYEPKISELQ
SVGSNKLGFPSGNNHAVQKVDECAIENEKGLALCSFFPATDKRVSHAQDHDSKVYQSTTE
VNDQQSSASALFSSTYVPQASTKEYTMNRSYSNPLNFDGLENSRHRDQNVVFGNSHVDP
NLNSTHHNRYITEKSTFQLSMDGTYAVQDNLHRRLETDRQGEVGVNLSESAFKGKTGDFE
SNFNMVFHSQSCDGGKVDEVGNSGKKIVTGFGCGNAKTNEDAVAGSIWRTGVENVMQG
GSVDNSTSAVQSSNCFQTYDVMSDKVQGLFGENEKYDGTTGFDGLRSDRNEPVEYSFMS
AHSLNSLQEEPRVLPYDVDIDQGFNSSFWLGKDDLMPNLADRNQ

>SlMBD1
MATVAPTCGPKLETLAHIDISKFSQSELHELSLCSDSAYDLRRTNEVVIPQIDRSLFNESAGS
RRQTYSRLRHQHHRSRVPGLHPSTSQPKPPCTSDPENHAILHFLKFFIHNPNSQSPPPPPITQ

PATSGVQEKTLLLMNEPDKKRKRGRKSKDNKKLKENGVEILNKNGEVVDLNNLENNGD
KLYSGELEKRTVRLQTEEEVLGFVRDLDGQWCSRRKKRKYVDASGFGDILPIGWKLLLA
LRRRDGRVWVYCRRIVSPTGQQFISCKEASSYLRSHFLSGEANQPTQQVDDTVAKSMSNF
HSDTSLVLSTDIQENPHSLQKGDMAKHNVVAHAVVPSSSTLHLHLSDVCLMEMDNLPEV
KVQDIFECYKCKLTFEEKNAYLQHLFSFHQRTTRRYRVGPSVGDGVIIRDGKYECQFCHK
VFEERRSYNGHVGVHVRNNARGTVDIAAAVAADKGVQSPHHDGLLSRTCKMDALIEIAQ
NAVVETSSARPATKDSSMPSSTSTDLDGNMSTNIDQVARSTTDIGLSDTKEFMTETCLEQG
RNQPDYTCVQVTKDKSSEILANKFNLRVISTNDSEQPEGDDAKKAGSNKVVQGPGNKQT
KENDDVRPETMELTFQEIASQNALTSSSVSMVQSLHNNFEHSEEGVGKKDGTDNVAVGK
GNSLTKANDVELETMELTFQDDATLDELTNFSLSMVQPSHSAFEHPESDDMKKDGNNQQ
AVCPGYSLTKANNDLESETMEFILQQNAIRDGLAGFSEPMVQSFHNSTGILSGSSKDNDEV
SAIGQNLDNGTGFEELRLDEIDHFEYSFDGGHESSSLPATSIGLGNDARMKEAFASVGFDS
GGIILNMEELNQLSTVCVWCRVEFKLEAYDTEAHSDSIGFMCPDCKAKISGHLESVFP

>SIMBD2
MGKKRGLPNWVPDGWKVEVRTRKGKKEKWYIDPSKGLKFCSKADVLHYLSSVDSNCS
KSSNTKEMENKGTNKCFIEKTASEGLPPGWIKELKVRKKGRKIRKDPYYIDPVSGQTFRSL
KQVSRFLETREPGRFESKPDDKCPDTLELEEPSSLFPAEADEQISLDCDASCKEVNSNQKL
KLGAEHMAHSSCVEGSMSSCEGLGDAVAENAAEEKIDKVVSDPAIEEHREKDDDGEVVS
NTVSGGHQEKEDIGKVILATASGEHQEEDDDGKVISATASGEHQEKEDDGKVVSKTVNGE
NQDKPSLDGVEKHDQKTFQRKRKKGMNMPSRASKRLAGIKADTSLKLQTNDQTQLTSV
RQQEGTQAATSDNPLNFTNTSKHVASTVTGITINKKVETGNLTIKRKQHLVTSPQTEGEKK
PSPAEPAKTVLASCDVDKKGETALESSLSDLWTDPCIEFAIKTLTGTIPVINEKKLDEIPGSSS
SKPSVNTPSSVGLPSDEIWADPCFMFAVKTLTGEIPIGDELCSQKIVQQQCTSSPNIRFEEFG
QANLPRYAAPEMSSYKQQPAVAPNAYQNRWFAKFGSHRPSPLC

>SIMBD3
MASNNMELPAPLSWKKLLMPKKGIRAKKNEVVFVAPTGEEIRNKRQLEKYLKTHNGNPG
MSEFDWTTGETPRRSARISQKVKAMPLPAVLEPAKKRQRTSSSTKKEEEMDAANAEKEN
MDKKEMESVIEATEGLENKENVVDNETVDKEVDTEHKKDEELSGGEVLQKQKSEASND
ALVEDGGKLAQNGIEDTIVEVEIEDKGDEMGAYKENIEHKIEGEISGTEVSDRKTETSNDT
QFGDGSKMAENVSVVTTAIDADFWNDSMDKDYFKGAFANAVIGDTNAAEAGIEVGEKP
GYGENLESQIDIEISGADDMNHDMPDMVASERNVAGGETFNIQDPAFVEGTGGFHEEHRP
LEEEKNKNSTGLVMDNGQINQPERAHTPQHQSSATISC

>SIMBD4
MASVTVDSSSATVPDGALQAESIPTVDLRLLSQSELYSLSLCSPAAFNPCRDDDVIIPKIDRS
VFNESAGSRKQTYSRLRLAPAATASASSAIRSRTPHLRNSPHPLQNPSPNNGPANSESSQIVT
LLKQLFGSGTQKNPTDLVPIRVDYSDSLSVPSHVPVPGLELANVGSIGQKRKRGRPRKNEN
GVRVAEVKVDEVVKDIVVYQNVDDSDKEIMNKDGIPVDLAVLGASVDPFGLELRRRTEG
LGSAEELLGFLGRLNGQWGSTRKKRRIVDADDFGSMLPKSWKLLLSIKRKEGRSWLHCR
RYISPNGRQFGTCKEVSSYLLFLRGERNENLPTYVNGSGTVEITNACALTSDLRIQDGGKK
ESSVFHNSSPAVGHGELQVLLNFGELSEVQVGDLLQCDKCNVTFNNKDDLLQHQLFSHQ
RRKSRNGGQSITDGVIIRDGKFECQFCHKTFEEKHRYNGHVGNHVKKQVKTVDGSLPIK

MGGGIEPVVPSGAMLREPIMQDSVVLPRNLTENAGVITDAGDNPAPTTKIQEDHMETDNK
LEAEGTSNGCHNQEGSSVSRSPISSNEKTCVDISKVIVGSNIEEPEQEGLLCSNDIVDSCGVS
MEDGKFFPTVDESKVENGRSVDTDSTTVLCSNPSLPGGNSLIKARQIPHTEDHSGKNIDD
MNGVSFLAETSKGNRYLKSSTGTPSCDKEGSTVDYVGVLSGCIGEHRPSSIMSDIENKECG
SLNSNDNKLIMKEDNNSIAQHLDKHVKDTAERDAVDVSACVLEELGQKKGDESSLLSPAC
DKNSEVESLIFNDLKTSAEEPKISELQSVRSNIVGFTSSDNYAVKKVAASDTEKEKSLAFCP
LFPAMNARASCAEDHDTKVYQSTLEGNDLQRSANALFSTTNVPEASTKEYTMHRSYNNS
LKESKFDGLEHPRHHDLNVVFGNSHVDLSANLNCTPFQLGMEETYGVQDNLQKRLETDK
HGEVGIDLSDSSFKGKTGDFGSNFNTVFPSQLWNEHKLNEVDNSGKKIITSFGCGDAKPNE
DVMAGSIWRVGVENVMQGGSAGNSTSVAQSSNCFQTYDVLSDKVPNLYGENEKYNGNT
GFDGLSSDRTGPVEYSFMSTQSSNSLQEEPRVLPYDVDIEQGFNSSFWLGKDNLMPNLAG
SNQVTMVIYMLPFCFAFLQKIHWMLECGWC

>SIMBD5
MERDQDAHLKNKVADEMEFVVQCSKCFKWLYIPTEERYYDSPIRKGFRSIPEDESYLKQH
SEYASQGVKLEKFSF

>SIMBD6
MERDHLDIATPGKADHNDIYGSSVASRRDKVKHEPKDSRSSASAGGIGTSNQNPKDVASL
AEEEEHNTDDEDNHSEDAQKQLVLYDPAAVGADEIALDADPVNSQPRRNSFPNYTTRLLP
SVGAFTVQCANCFKWRLIPTKEKYEEIREHILEQPFYCETAHEWRAELSCADPPDLTQDGS
RLWAIDKPNIAQPLLGGNGF

>SIMBD7
MASPMEKGSHDDEIVSVELPAPPSWKKLFTPKQGGTPKKSEVVFIAPTGEEVKNRKQLEQ
YLKAHPGSPGISEFDWSTGETPRRSARISEKVKAMRPPSLLESPKKKRRTSSGTKKDSKEK
AAAKAEMGSAETKGMESSKEENENLEKKAGEAEAEMQDKGKKEAEAVVEDERIEDAKL
PPAEKPDSESEEFHSADDGNQDKSENAEAEMEDKKKKEVEAVEKDKCLKGAELPSGDER
EPESEEVHSADVGRHKSENAGIEEKQVSEEKLESQNKLEELAAEGTNVTVGGSAGQAEH
AVDGVSKDAHSNDGEDGPKEEEKKTEGTELAMENNNINQPGLVHPQQHQSPAPISC

>SIMBD8
MAKDSPKTPKSRSATNPSVGVWAVQCEKCLKWRRIATQEQFEDIRSRFTEEPFNCPNGTCD
DPADIEYDASRTWAIDKPNLPKTPSGFKRELYLRRDYSKMDTYYFTPLGKKLRSVTDVTTF
LEQNPQLSDVKPSDFSFTSPKVMDDTIPSTALLANSHKKGVASSTK

>SIMBD9
MSSSVEMNEVVSIELPAPNGWLKRFLPKKGGTPKKNEIVFTAPTGEEITTKKQLQQYLKSH
PGGPAITEFDWGSGEAPRRSTRITGKAKTAPLAAESVTPTKRNRKSSASKKDVKDKKEQE
ETEAAKDVDMPEADKQEKDGVSVEAEKHEKDAVAVEAGKHEKDAAAVEAEKVVVQKH
DEGKDENKEEMPSTDVGIVKENQDSKMADHTAEQDVQMADHAAEKKDEMHSSAVDVV
EKNQAEKMGEGQKAGDGPSKEAEVEKDDKMSDCVAEKKDKTSDVDAVKSDDPTEDGK
AEDAPADVSAADKNVGDAFMVKEVPIGKAADEAEATDNGVNTDEINP

>SIMBD10
MERDQDAHLKKKVADEMEFVVQCSKCFKWRYIPTEERYEKIREHLLECPFYCEDAREWR
PSISCDDIPDITQKEKKLWAFDKPSIPQTPSGWKRIVKIRTRGTIFADVYYDSPTTKGLRSIPE
VERYLKQHSEYASQGVKLEKFSFKTPRSLQQEYSKKRSPTPPTPSDDINGANAGMSMCMC
M

>SIMBD11
MAVEDSPDWPPPGWTEDVKVSKGRKIKYYTNGETGKKFYSKKEVARYLKTKDTCDDVT
QAMNIQGKSFSENNVSDMDNQDGSFAKDNGSQMDNQDNCCSENNVSQTVGKPNNSHE
WLPPGWIVELKTRKSGSHAGLSYKVYIDPSTGSKFYSKPEVSKYLKTMKQNNIAGEGQTH
GDGEISTTNQKKSEDPKIIRGGGQTRKSKRLKSDTDQASPGIEEGSFSKLKASQDFEEVGE
QSCTTKRKKSGITRTTKTYPSHGAGEVFILNEKTSLGSKENGAQSPQSKRQKPGSGCPSAK
SVSSVAVDCDSADELPPGWKKESRKNDRGTRNYLLYTDPVHGYKFHSKKEVLRYLQTGD
ATSCARRPTKRNVASTTKDDSPTTDDASLKRVGGSMTGRQLFSTDELKGGESFGTCSPAQ
QVESSKKQPDCSVSDPNAIITSILAEINENHSFENVVGDAEIETEKHSSKATQQSKKSRKTE
SLNLGRRVSKRLAGQNAEAAADLDLGERALPAVADKSASLSVNACSQELLQDSDPTPETG
NSDQASLNGDPSLDDLPPGWKKEIKITKKANGIRKDPLYIDPVDGYVFRSKKDVFRYLQT
GDISSCAIRPVKRDLDAAMTDSSPTTVDTNSKKLVCSVTERQPFAAKESRGRKRSVTCSPK
VQAESSKKQPESSASNANTIITSSEADIIVKQNAEISLDTEPEIRDSSADTKVIDAESNAIKRL
SAVSTPQSDLPSEQQLPENEPEKHISKEKPEQSRRSRAKKPLTPGRRISKRLVGHSPEPVADL
DLGERAFRAVVKKSASLGIPLNVSGQEFSQNKDPTVGTGNSDQAPLSREASGLEFPPDLGK
EMLTQNEDLTEGTDPTVGSGNSDQASLRASGDELSPGLKKEILTHYKDPTVGSGNSDQAY
LSRKASLDEIPPGWEKEMLGQNKYSTVGTGNSDQASLSRKLSPDEIPPGWENQIPPGWKY
EIPAQNKDPIIGTSNSDQASLSREKASLDEIPPGWEKEILAYNKDPAIRTGNSDQASLIRELA
SADEFPPGWEKEMLTRNKHPTVGTGNSNSLDDIPPGWEKELLVQQKDHTVGTGNSYQSF
LSKEASVDGLPPGWKTEFRIRKNASVIKKDPYYTDPVHGYVFRSKKDVMRYLQTGDIRSC
AVKPTKRDPGSTMKDNSPSTHDTSSKKLRCSLTGIQLSATDESKGSKCSVTCSMALQAENS
SEHPKSSMFNLNTSISSIVADITEKHSYENLNEDTEIRLDVEPKIRDSVGNTKAAAAETVDV
RRSLVVSPQLDFLPEQQLRENEKEKHSNKGVPARLRKIRNSKSSTPVRRVSKRLSRHNPEM
VTDLNLGERALHSVVNSSASSGLPMNVSGGQSAQQTDLATGDSDQPSLSRKASSRDDPLE
VVKCLSEGQAFKENIDERIWRDSQPAGISYERGKVHLEQHALNERPTAKPAKEEEDGQNR
LLNNSQLAESASGRRVFPAEDRSVSERSTVEEVSEKRNGEYKQWQYPQLAEPSWRSVDL
NVENQQWQDVQLAPSQRFVDLNMENQEWQDLRLPPSQRSVDLNVGNQQWQASQLAEP
SHRNLDLNVDSQPVKEKQTGELVTENQDEQNRRLHAQLASYPFGDYWSDPCMEFAFKTL
TGALPVEDTLTFQGSTHQEYNTSYTQADDGCFELPLFNTSSFYLNDAPNHCAPSVEHVVK
EQPPINQTFLPTGHNSIPGHSSVVSQNPGLTFLPNGNNSIPGCSSVVSQNPGLNTQAKDYQS
KFKSHR

>SIMBD12
MEFAVQCSKCFKWWYIPTEERYEKIREHLLECPFYCEDAREWRPSISCNYLPDITQEKKYG
HLISLVFLSLLRDGNKLYYDSTTGIRLRSIPEVKRYQHSEYASQGVKLEKFSFKTPRSLQQE
YSNKRSPMPPTHSDAINGANAGMSMCMCM

>SIMBD13

MLVLKRRLQMKWSLPCSDLNVSNGATYLQRRDAPEWRPSISCNDIPDRTQKEKKLWAFD
KPSIPQPPLGWKRIVKIRTRGTNFADVYMKQHSEYASQAVKPEKFSFKTPRSLQQDYAKKR
SSTPPTPSDYINGDNAGMSMCMCM

>SIMBD14
MTKVDAADSFIQPKIDTSDDFINDGIYSDLLLPPGTYIDVELDAREDTITPNKGDVPYEGNL
SFVPRSKLKIRRKIAPIHHVNLDRPLWLSQDWKFETKVRTIGATVGRVDKLYIEPIFKSKFRS
KLEVEEFLKTGCKRTRKKYCHNHDGAT

>SIMBD15
MTEAASVSSIPPATVTGDDFRDDSLPRDPLLQSGTYIDAESNVDTTPNKGVRPNAGNVPDL
TPAPAEGTQEIQSVDTVAQTVTPLRYGSRSAEVSVERPTWLPESWRFEAKVRTSGATAGTV
DRYYYEPVTGSKFRSKTEVLYFLETGGKRKKAITGTTGSGTDATPSETPPIKKQKKSISKTK
KVTSFYFDSGNPPQSVCWVQTDTSADTWTPSCNGSVVPGTRKQEWDAVFLSVSKLKRRN
TQTGR

>SIMBD16
MENKDVAVEKTDAGPIELPAPPGWKKRFTPGKSSTPRRYAIVFVSPDGDEIKNKKQLDKYL
KSHPGGPPASEFNWGTGDTPRRSSRLGGKSEAMETPESDTPSTKRQRKSSSKKEAKEDGS
ERKAEGATEKETKANDEPALPDAEDLEAQDVEMASKNLTDGDNTKDEKEKTNDGEIVPK
EPLPSESEDKMEIVQEKEEILDEGNIEKMKNREAEDKPLDNSLKTDNKTLPLNVLEENKIE
SKPAGTDASSLSVEHSKSTSGSQEEIAEEAPAAEDALDALEENKIESKPAGADASSHSAELS
KATSVSQEEAPAAADPLVQNSNDGKVHENEQIVREIPIEEINGTESAAACVNNSVQQEATVI
NQATSS>SIMBD17MEKQNSLRRHRSGAEYPTSGQLTASSSAITPYRPLSSRFKLPPGWGIE
QVLRSSGDRVDRYYYEPGTGQKFRSLRDVERRLNGEIFAPRNRASGVRNYPKSSLSRKMV
ICDGKIVRMDEEQLNEWAIVPSTRAATLPYDLPDGWVIEEVPRTDGSSMVDKYYYEPGSG
QKFRSRIAAQRYLAQMRENVPLSATLEELKENKPLSKMFKLYHHAKVKISSM>SIMBD18
MAKSVEKNEVVSIELPAPSGWSKKFLPKKGGTPKKNDIVFTAPTGEEITTRKQLEQYLKSH
PGGPPVAEFDWGTGETPRRSARITGKAKATQSPTESEPAKKRGRKSSASKKDSKDKEVTK
ETEAAKDDDMEEAEKHEKDTAAMESEEDVEKKENENPNETQDGESEVEKKDEIQSSEKD
VVKENLDEGQNVHDKVEDAQVEKDVQMADNVGPSQDVEEAPVVKAADGPDATKINEE
EKDVQVQEVEKVPTEEAHIEKDVKMADNDVEEETPADKTAVGPEATNINEEGKDVQVQE
VEILPTEEAQVEKDVKMTDNIGHPDVVEETPADVPEATKLNEEEKDVQIQESENKPNEEA
QVEKDVNNVEEALAEKAAEEKDVQVQEAEKVDPSAAEELKHQAEGKSTDDQDNLMNIE
ISKVEGEVTENGSNANEAKP

>CaMBD1
MPVGDSSDWPPPGWTEDVKVTNGRKIKYYTNVETGKNFYSKKEVTRYMNTKETCHDVT
QAMNDQDKSCSENNVSQMNNQDKSCSEKNARQMNNQDKSCSGKNIGQMNNQDKSCSE
NNVSQTVGEPNNAHEWLPLGWIVELKTRKSGSHAGTTYKVYIEPSTGNKFYSKPEVSKY
LKTMEQNNIAEGQIHGNGEVSSSNQKIPEDSKSIKGGGQSRKSKRQKSDTDQAVHGVEEE
SVSKKKASQDSEDVGEQRRISNRKKSGTNRRSTKTSSHGAGEDFPLNEKTSLGSKENSEQ
SPLSKRQKPGSDSPSAKSVSSVAGDSDSVDEFPPGWKKEFIIQENDRGTRKDLVYTDPVHG
YKFRSKEEVLCYLQTGDASRCARRPAKRNVASTTKDDSPLTDDASLKKSVCFMTGRQLFA

TDELKGAKSFGTCSPAQQVESSKKQFDCSVSDPNTIISSILAEINENHSIENVIEDADIESVK
VGVMHPSAVSTKSDLLPEKQLLENEQEKLRHKAAKKQSRKSKTKESLNLGRRASKRLAG
QNPEAEAANLDLGERALPLIVEKSSSPSLSMNVCRQELLQDSDPIPETGNSDQLYIDPVHG
YVLRSKKDVFRYLQTGDINSCAIRPVKRDKDATMKESSPTAANADSKKLECSVTERQPFA
AKESRGRKRSDTCSPEIQAKNSKKQPESRESNANLIVTSSAADIYAKHSDENVIENAGIRDS
SADRKVIAAQSDAIKKSSAVSTPPSDLTPEQQLPENEHEKHINKEKLEQSRRSRTKKPITPG
RRISKRLVGHSPEPVADLNLGERAFRAVVKKSASLDIPLNVSAQEFSQNIDPALGTGSFDQA
SLNREASGNEFPPDREREIPIQNIDPTVGSGNSDQASLSRDASGDELSPGLEKEMLAQNKD
PTVGTGNSDQASLSREASGDERLEKETLAQNKDATVGTGDSDQASLNREASGDKLWPGL
EKEMLAQNKDLTVGAGNSDQASLSREASGDKLPRGLEMEMFAQNKDPTVGADNSDQAS
VSREASRDKLLPGLEKEMLAQNKDPTVGTGNSDRASLSRKASGNELSPGLEKEMLARSK
DRTVGTGNSDQASLSRKASGNELSPGLGKEMLAQSKDRTVGTGNSDQASLSRKASGNEL
SPGLKKEMLAESKDRTVGTGNSDQASLSRKASGNELSPGLEKEMLTQNKNWTVGTGNSD
LASLSTKASLEEIPPGWENEILGQKKYPTVGTGISGRVSLSRKLSPDEIPPGWENVIPPGWK
NEIPAHASSLGEIPPGWEKEILAYNKVPTIRTGNSDQASLSRKLASADDLPPGWEKEMITRN
KVPAVGTGNSLDDIPPGWEKELLFQTKNPKVGTGNSNQSYLSREASMDELPPGWTMEFR
MRKNARGKDPYYMNPMHEYVFRSKKDVMRYLQTGDIRSCAVRPTKRDPYSTMKDDSPS
NHDTSSKILGCSLTGSQLSATEESKGRKCSATRSLALHAESSNEHHESTVFNLNTSISSTVA
DITDKHSCENVNEDANIGVSMRSRKMKNSKSSTPVRRVSKRLSRQNQEMVADIDPGERAV
VNNSASSGLPLDASGGELAQQPDLATGDSDQPSLRRKASSRDDPLKAVKYLSEGQPFKEQ
KVVNGLTSEKQNDEIILRGSQPARISFESRKFHSEHQAVKESPTAKPGNEVQDSQYRLLNNS
RPVELASGRMVFPVEDQYVSERSTIEQVSEKRDYENRQWQDSQLAEPSWRSVDLNVENQ
QWQYSQLAPSQRSVDLNVENQQWQYAQLAPSRKSVDLNVENQQWQYSQLAPYSQPAPS
RGSVDLNVENRQRQYSQPAPSQGSVDLNVEKQHSQPAPSQRSVDLNVENQQWQESQLAP
SQRSVDLNVVNQQWQDSHLAPSQRSVDLNVVNQQWQDSQLAPSQRSVDLNVANQQWQ
DTQLAEPSQRSVDLNVANQPIREKQTGELVTENQDEQNRRLHAQLASYTFGDYWSDPCL
EFAFKTLTGTLPVEENTPFQGSAHQEYNTSYSHADDGCFELPLFNTSSFYMNDVPHHCAPS
EEHIVKEQPPINHTFLPNGNNSVPGCSNVVTQNPGLAFFPNGNNSLPGYSNVSQNSGLDPH
AKDYQSKFKSQR

>CaMBD2
MEKNQVLPVELPAPPSWKKLVLDICIRVLSILLVMSEEFCIASYLYLPGWLSSVSFEAMPKK
SVRAKKNEIVFVAPTGEEIRNRRQLEKYLKTHDGSPGISEFDWTTGEAPRRSARISEKVKA
MPPVAVLEPTMKRRRTATKKDEDVASVEKENMGKKDMEPAIENKEGLEEEENDVEDEVV
DKGKDEQTAKEVETEYIKELEMFDGEAPKRQKYKACNDPQSEDGGKLAENVTQETAVEV
EMENKEDEMGAIKGTADHKKEKEMSDREVSERKIEASNDPQSEDGGKLTENVARETAAE
VEMENTEHEMGANKEAADHKKGEEMSDREVSERKIEASNETQSQVEDGGEMAENGRG
VTIAIDANIWNDSMGQDYFMGMFPDAVIEETNVAEAGAEVGENLVSQTGKDISADDMNR
DIADKVAPEINVAVGETYNIQDPAFTGETSDYHEEHRSLEEDKNKNRAGLMMDSGKINQP
ERAHTPQHQSAATISC

>CaMBD3
MGKKRALPNWLPSGWKMEVKARKKGKKEKWYSDPSKGVKFCSKADVLHYLSSVDSNC
SKSSNMKELEEKDTEKCSTEAASEELPPGWIKELRARKKGRKIRKDPYYIDPVSGQTFRS

MKQVSQFLETRESGRIESKPDDLCSGTLELGEPSSSLPVEADKQISLDSDASDQVANSNQK
LKLGAEHIGHSSCVEGGMSSFEGDSVPENAEEKNVGKVVPNTTSGEHYKKDDGKVVSDP
ACGEHQEKEDHGKVVSDPASGERQENEDDGKVVSDPASEEHQEMVDDGKVVSDPVSGE
NQEKQSPGVKKHEMETNQRKRKQGVNLPRRASKRLAGIKADTSLKLQANNQIHLATVRQ
EGMQAATTDNPVNFINTSKHVATVVTGITISKKVETGSLAIKRQQDLVTSPQKEQEKPSPAE
PAKTFLASREGDKKGETALESSLHDLWTDPCIEFAIKTLTGTIPVINEKEVGEVQGSCPSTAS
ANTPSSVGLPSDEVWEDPCFMFAVKTLTGEIPIGDNLFSQKIVQQQCTSSQFQGNNGLVLP
NIRFDEFVQANRSQLGAPEMSSYKQQPAVAPNACQNPWFTKFGSHRPSPLC

>CaMBD4
MLKQLFGSGSSTAANPTDLVPIRVDYSDSFSVPAPAPVPVPELGNVGSIGQKRKRGRPKRN
ENSGVVEVKAAKADVEVKDIVVYQNVDERDDRDKEIMNKDGVPVDLAALGALEDPFGQ
ELRRRTEGLGSGEELLGFLGRLNGQWGSTRKKRRIVDAGEFGSTLPKGWKLLLSVKRKG
GHAWVHCRRYISPNGRHFGTCKEISSYLLFLHGERNENLPTYAKSSETVEITNACALVSTS
ADLRVQDGGEKESSLFHNSAPAVGHGEFQGLLNFGELSEVQVGDLLHCDKCNVTFNNKN
DLLKHQLSSHQKRRSRNGGQSITDGVIIKDGKFECQFCHKTFEEKHRYNGHVGNHVKYQ
LKTADGSLPIKMGGYVEPVVSSRAMLREPIMQDSVVPLRNLTENAGASVDDGDNHAPPS
KIQEDDHMETDDKLEGPAEGTNKGCHNQEDISSDRLVDAESTTALCRNASLPDENSLISAR
QVPLTEDHSGKNIDVLNGVSFLSETSKENRDFKSSLGTPSCDKEGSTVDYVGVHSGQDSY
SFVPHPDEHVNTAERDVTDVSACVLDGLAQQKGDGSSLLSPSCDKNSEIESQIFNDLKRSA
DEPKINQLQSVTSDIVGFTSSNNHAVQKVDASNIEKEKSLAFCSLFPVTNARASCAEDHDT
KVYQSTMEVNDQQRSASALFSSTNVPEASTKEYTMNRSYNNSLDESKFGLENSRHHDLN
VVFSNSHVDLGANLNCTTFQLGMEETYGVQDNLHRRLETDKHGEVGIDLSNSTFKGKTG
DFGSNFNTVFHGQLWDEQKVDEVDNSGKKIITSFGCGDAKPNEDVMAGSIWRTGVENVI
QGGSADNSTSVAQSSNCFQNYDVLSDKVHTLFGENEKYDGNTGFDGLRSDRSGPVEYSF
MGTHSLNSLQEEPQVVYIEQGFNSSFWLGKDNVMPNLAGRNPTTVVCVWCRNEFYQEPD
QLGAEAGSIGFMCPTCSGRISGQFSFM

>CaMBD5
MERNHLDFASPEKADHNDIHGSSVTSCGYSMKNEPKDSRVSAGSGVIGTSNQNPRDVISS
AEGEEHNTEDEDNNSEDAQKQLVLYDPAAVGAGEIELVADPVDSQPRRNSFPNYASRILPS
VGAFTVQCANCFKWRLIPTKEKYEEIREHILEQPFYCETAHEWRAELSCNDPPDLTQDGSR
LWAIDKPNIAQPPPGWERLLRIRGEGGTRFADVYYVAPSGKRLRSMVEVEKYLQEHPEYV
AQGVSMSQFSFQIPRPLQDNYVKKRPYRPALAHDEIEPGMPGPFEPLHPL>CaMBD6MEEF
EEIRKRFVEEPFHCNNKPNGSCDEPADIEYDSSRTWVIDKPNLPKTPSGFKRKLYLRQDHSE
MDVYYIAPSGKRLRSIVQVRAFLRQNPEFADISVSDFTFTTPKVMRNAASSNAVLSNSSNK
GAASTEQNECVD

>CaMBD7
MAKDSPKPPKTTPRAPTNPTVSIWAAQCGKCFKWRTLSTQEEFEEIRSRFAEQPFNCDNKP
NGSCDDPPDIEYDSSRTWAIDKPNLPKTPSGFKRELYLRRDYSKMDAYYVTPTGKRLRSLI
EVGNFLQNNPEFSDLSVSDFSFVSPKIMDDTIPSTAVVANSNKKGAPGTAK

>CaMBD8

MGLVVKMLEADTVITNYTRGRCRSILMTLRGKGLLKNLFTDNKPNGSCDDPADIEYDPR
WTWIIDKPNLPKTPSGFKRKLCLRNDHSKMDAYYIAPSGKRLRSLVELCSFLQQIPEFIDIS
VSDLTFTSPKVMDDATSSKVVLPNSSNKGAKENAHSFDLDPCGSKGRLDCTCMTDLYVK
VPEE

>CaMBD9
MGNALKGGQFQHRKSLWKFKEGFLNNPLTAIEKTNLPNAPSGFKRELYLKRDYSKMDAY
YFNTGKRLRSLTEMGNFNQQNPEFSDLPVSDFSFFIPKVMDDTIPSTTILSNSNK

>CaMBD10
MATVASTCGPTLESFAHIDITKLSQSELHALSLCSDSAYNLRRNNDIVIPQIDRSLFNESASS
RRQTYSRLRHKHHRSRVPGLHPSSSQPKPKPVSTSYPENHAILHFLKYFVNNPNSQSPPPPP
PPPPPPPVPLPPVHPPPVIQPDTSGIQGKTLLSRKRGRKRKDDKKVKVEMVNKKGEVVDL
NSLENNGDRLYSEELEKRTVGLQSEEELLGFVRGLEGQWGSTRKKRKYVDASGFGDALPI
GWKLLLALRRRDGRVWVYCRRVVSPTGQQFISCKEASSYLRSRLLSGEASQPSQQVDDT
VAKSASNFHNDTSLVLSTDIQENPHALQKSDVAKHDIVAHAVVPSSSTVDLHLSEICLMEM
DKLPEVKVQDIFECYKCNLTFEEKNAYLQHLFSFHQRTTRRYRVGPSVGDGVIIRDGKYEC
QFCHKVFEERRSYNGHVGVHVRSNARGAVDVAAAAVAANKGVQSPHHDGLLSRTCKMD
ALIEIAQNSVVETSSAKPVTKGSTMPSSTSMDLDAIMATNIDQVARSNTETGLSDAKEFKA
ETCPERGRNQPDNTCSEVDKDKSGEILVNKFNPRVISTNASEQPEGDDVKKAGSNKLMQG
MGNKQTKENNDVQPGTMELTFQEIASQNPSTSSSVSMVQTLHDFEHSEDGVVKKDGIDE
VVMGKGNCPTKANNVESQTMELTFQGNATLHGLTSSSASISHGAFEHPESDNMKKDAND
QLALCLGDSLTEANADAESKILEFSLQQNATRDGLASFLEPMVQSFHNFTGIISGSSKDNG
KVSAVGQNLDNGTGFEELRLDEIEHFEYNFDVGHVSPSLPPTSIGLGNDARMEEAFASVGF
DSGGIILNMEGLNQLSTICVWCTAEFKLEAYDREAHSDSIGYMCPDCKAKISGHLESVFH

>CaMBD11
MSGVFITVIGKETKWPIFWLIGVAGVEKRFSVWTTTGCLKMLELLPKRGGIPKKNAIIFTAP
TGEQITTKRQLEQYVRSHPGGPSIAEFDWGTGDSPRRSPRIEKQKATQSPAKSEPAKKRSR
KSSASSSASKTDTAVLEAQKDIEPKEDENVDGESEAEKKEEAETTEKDVQKKDEMQSSEN
DLVKENLDADGQNADDNVQDSPSEAAQVEKDVEMADNVVHTDNVEEAPVDKTADGPE
EEDARVEEVESLPTEEVQVVHPDHAEEVPADKAVDGEATKINEEDVLQVQEPENKRTEEA
QFEKDMKLADNIGHPNDVDEAAADKAAEEKDVQVEEVESIPTEAEKLDPSAAEEKKDQA
EVEQKAAAATKSTGDQDDLTNIDIINVEGELTDSGSNANEARP

>CaMBD12
MVCMDQEKLNEWAIVPSTCAATVPYDLPDGWVIEEVPRRDGSLVDKYYYEPGTGLKFRS
RIAAQRYLAEMREDVPLSTSLEELKENKPLSKMFKLHHHARKSPPCTRNIVRENSDKSSFL
SPPAKVNWVLSSPRGDAWNPFIAGTPIPDSVKQQWTKRFMLLMNDETLNAENSN

>AtMBD1
MLPFPAMNLKKSRSENSSVASSGSKIEEQTEKSAEPTTIKVQKKAGTPGRSIDVFAVQCEKC
MKWRKIDTQDEYEDIRSRVQEDPFFCKTKEGVSCEDVGDLNYDSSRTWVIDKPGLPRTPR
GFKRSLILRKDYSKMDAYYITPTGKKLKSRNEIAAFIDANQDYKYALLGDFNFTVPKVME

ETVPSGILSDRTPKPSRKVTID

>AtMBD2
MSMSQSRAVQRSSSPNEDRGENQLVVYDLKGNDDTEEEVLPVQSQPLSSRTQCPSIGAFT
VQCASCFKWRLMPSMQKYEEIREQLLENPFFCDTAREWKPDISCDVPADIYQDGTRLWAI
DKPNISRPPAGWQRLLRIRGEGGTRFADVYYVAPSGKKLRSTVEVQKYLNDNSEYIGEGV
KLSQFSFQIPKPLQDDYVRKRPARLLDSIDNTNTPVAKEANPLAWISPDDHISLQLGTPTES
GLNNSHYQPSKKKKTSTLSIFGSNDELADR

>AtMBD3
MCVVKTTLIDSYAAQCWKCLKVRSIESQEDYEEIRSKTLEKFFECKRCEEPGDMVMNFDS
LTMRWFQDEHSIPKTPQGLKRVLVVRTNCVKVDVYYESLAPRRKRFKSIKEVATFIEDKEE
FKDMTLEEVSFAAPKRLKLKKKPVDSHSSSRNTEEDGVSRDA

>AtMBD4
MKEEEEIGKPAKPKAKKDVAPGRLIDTYAAQCDNCHKWRVIDSQEEYEDIRSKMLEDPFN
CQKKQGMSCEEPADIDYDSSRTWVIDKPGLPKTPKGFKRSLVLRKDYSKMDTYYFTPTGK
KLRSRNEIAAFVEANPEFRNAPLGDFNFTVPKVMEDTVPPDPKLGSPFPSTTTTTSEKSSV
KQSHN

>AtMBD5
MSNGTDQAQPPPENPATPVDSKSRKRATPGDDNWLPPDWRTEIRVRTSGTKAGTVDKFYY
EPITGRKFRSKNEVLYYLEHGTPKKKSVKTAENGDSHSEHSEGRGSARRQTKSNKKVTEP
PPKPLNFDFLNVPEKVTWTGINGSEEAWLPFIGDYKIQESVSQDWDRVFTLVTSQNAGKT
MF

>AtMBD6
MSDSVAGDFPPDPLLASGAFISSAGDGTLDSSAKRRPIQGGIGISGSGESVRIGMANGTDQV
NHQTESKSRKRAAPGDNWLPPGWRVEDKIRTSGATAGSVDKYYYEPNTGRKFRSRTEVL
YYLEHGTSKRGTKKAENTYFNPDHFEGQGSNRVTRTATVPPPPPPPLDFDFKNPPDKVSW
SMANAGEEGWIPNIGDVKVQDSVRRDWSTAFTFITSRNPSKVSA

>AtMBD7
MQTRSSSSPSANHRRETQLQIADPTSFCGKIMPGWTVVNRPRSSNNGVVDTYFIEPGTGR
QFSSLEAIHRHLAGEVNDRRLTRAGSFFQDKTRVYEGSRTKQDHCGVEYASKGFRLPRGW
SVEEVPRKNSHYIDKYYVERKTGKRFRSLVSVERYLRESRNSIEQQLRVLQNRRGHSKDF
RLPDGWIVEEKPRRSSSHIDRSYIEPGTGNKFRSMAAVERYLISVGNITLDSVSMVHSERLP
LLMNRNGIRFQSEVIDPNPPKKVKWVLTGSGGNMFTANVRGSNVSSLVKHTWSEAFVSLI
EDRS

>AtMBD8
MDDGDLGNNHHNFLGGAGNRLSAESLPLIDTRLLSQSELRALSQCSSLSPSSSASLAASAG
GDDDLTPKIDRSVFNESAGSRKQTFLRLRLARHPQPPEEPPSPQRQRDDSSREEQTQVASLL
RSLFNVDSNQSKEEEDEGEEELEDNEGQIHYNSYVYQRPNLDSIQNVLIQGTSGNKIKRKR

GRPRKIRNPSEENEVLDLTGEASTYVFVDKTSSNLGMVSRVGSSGISLDSNSVKRKRGRPP
KNKEEIMNLEKRDSAIVNISAFDKEELVVNLENREGTIVDLSALASVSEDPYEEELRRITVG
LKTKEEILGFLEQLNGEWVNIGKKKKVVNACDYGGYLPRGWRLMLYIKRKGSNLLLACR
RYISPDGQQFETCKEVSTYLRSLLESPSKNQHYYLQSDNKTLGQQPVIANESLLGNSDSMD
SETMQYLESGRTSSEVFEEAKAVENGNEADRVKTSLMQKDDNADFLNGVEDNDDDMKK
RDGNMENLATLSNSEMTKSLPTTTNELQQYFSSQINRVQ

>AtMBD9
MEPTDSTNEQLGDTKTAAVKEESRSFLGIDLNEIPTGATLGGGCTAGQDDDGEYEPVEVV
RSIHDNPDPAPGAPAEVPEPDRDASCGACGRPESIELVVVCDACERGFHMSCVNDGVEAA
PSADWMCSDCRTGGERSKLWPLGVKSKLILDMNASPPSDAEGYGAEETSDSRKHMLASS
SCIGNSFDYAMMHSSFSSLGRGHASLEASGLMSRNTKMSMDALGSHNLGFGFPLNLNNSS
LPMRFPSLDPSELFLQNLRHFISERHGVLEDGWRVEFRQPLNGYQLCAVYCAPNGKTFSSI
QEVACYLGLAINGNYSCMDAEIRNENSLLQERLHTPKRRKTSRWPNNGFPEQKGSSVSAQ
LRRFPFNGQTMSPFAVKSGTHFQAGGSLSSGNNGCGCEEAKNGCPMQFEDFFVLSLGRIDI
RQSYHNVNVIYPIGYKSCWHDKITGSLFTCEVSDGNSGPIFKVTRSPCSKSFIPAGSTVFSCP
KIDEMVEQNSDKLSNRRDSTQERDDDASVEILLSEHCPPLGDDILSCLREKSFSKTVNSLR
SEVDSSRVDFDKNLSYDQDHGVEIGDIVVEEDSLSDAWKKVSQKLVDACSIVLKQKGTLN
FLCKHVDRETSEINWDTMNEKDNVILSLSKFCCSLAPCSVTCGEKDKSEFAAVVDALSRW
LDQNRFGLDADFVQEMIEHMPGAESCTNYRTLKSRSSSSVPITVAEGALVVKPKGGENVK
DEVFGEISRKAKKPKLNGGHGVRNLHPPPGRPMCLRLPPGLVGDFLQVSEVFWRFHEILG
FEEAFSPENLEQELINPVFDGLFLDKPGKDDKRSEINFTDKDSTATKLFSLFDESRQPFPAK
NTSASELKEKKAGDSSDFKISDSSRGSCVGALLTRAHISLLQVLICELQSKVAAFVDPNFDS
GESRSRRGRKKDDSTLSAKRNKLHMLPVNEFTWPELARRYILSLLSMDGNLESAEIAARE
SGKVFRCLQGDGGLLCGSLTGVAGMEADSMLLAEAIKKISGSLTSENDVLSVEDDDSDGL
DATETNTCSGDIPEWAQVLEPVKKLPTNVGTRIRKCVYEALERNPPEWAKKILEHSISKEIY
KGNASGPTKKAVLSLLADIRGGDLVQRSIKGTKKRTYISVSDVIMKKCRAVLRGVAAADE
DKVLCTLLGRKLLNSSDNDDDGLLGSPAMVSRPLDFRTIDLRLAAGAYDGSTEAFLEDVL
ELWSSIRVMYADQPDCVDLVATLSEKFKSLYEAEVVPLVQKLKDYRKLECLSAEMKKEIK
DIVVSVNKLPKAPWDEGVCKVCGVDKDDDSVLLCDTCDAEYHTYCLNPPLIRIPDGNWY
CPSCVIAKRMAQEALESYKLVRRRKGRKYQGELTRASMELTAHLADVMEEKDYWEFSAE
ERILLLKLLCDELLSSSLVHQHLEQCAEAIIEMQQKLRSLSSEWKNAKMRQEFLTAKLAKV
EPSILKEVGEPHNSSYFADQMGCDPQPQEGVGDGVTRDDETSSTAYLNKNQGKSPLETDT
QPGESHVNFGESKISSPETISSPGRHELPIADTSPLVTDNLPEKDTSETLLKSVGRNHETHSP
NSNAVELPTAHDASSQASQELQACQQDLSATSNEIQNLQQSIRSIESQLLKQSIRRDFLGTD
ASGRLYWGCCFPDENPRILVDGSISLQKPVQADLIGSKVPSPFLHTVDHGRLRLSPWTYYE
TETEISELVQWLHDDDLKERDLRESILWWKRLRYGDVQKEKKQAQNLSAPVFATGLETK
AAMSMEKRYGPCIKLEMETLKKRGKKTKVAEREKLCRCECLESILPSMIHCLICHKTFASD
DEFEDHTESKCIPYSLATEEGKDISDSSKAKESLKSDYLNVKSSAGKDVAEISNVSELDSGL
IRYQEEESISPYHFEEICSKFVTKDCNRDLVKEIGLISSNGIPTFLPSSSTHLNDSVLISAKSNK
PDGGDSGDQVIFAGPETNVEGLNSESNMSFDRSVTDSHGGPLDKPSGLGFGFSEQKNKKS
SGSGLKSCCVVPQAALKRVTGKALPGFRFLKTNLLDMDVALPEEALRPSKSHPNRRRAW
RVFVKSSQSIYELVQATIVVEDMIKTEYLKNEWWYWSSLSAAAKISTLSALSVRIFSLDAAI
IYDKPITPSNPIDETKPIISLPDQKSQPVSDSQERSSRVRRSGKKRKEPEGS

>AtMBD10
MENTDELVSIELPAPASWKKLFYPKRAGTPRKTEIVFVAPTGEEISSRKQLEQYLKAHPGNP
VISEFEWTTGETPRRSSRISQKVKATTPTPDKEPLLKKRRSSLTKKDNKEAAEKNEEAAVK
ENMDVDKDGKTENAEAEKEKEKEGVTEIAEAEKENNEGEKTEAEKVNKEGEKTEAGKE
GQTEIAEAEKEKEGEKAEAENKEAEVVRDKKESMEVDTSELEKKAGSGEGAEEPSKVEG
LKDTEMKEAQEVVTEADVEKKPAEEKTENKGSVTTEANGEQNVTLGEPNLDADAEADK
GKESKEYDEKTTEAEANKENDTQESDEKKTEAAANKENETQESDVKKTEAAVAEEKSND
MKAEDTNRSLEANQVQQQQGAAASVSC

>AtMBD11
MGGEEEVVSVELPAPSSWKKLFYPNKVGSVKKTEVVFVAPTGEEISNRKQLEQYLKSHPG
NPAIAEFDWTTSGTPRRSARISEKTKATPSPDKEPPKKRGRTKSPVSKKDAEGEKSEGGGE
ENSHVKDTEMNPPEGIAENENVTDKNGSGETERVNDAKENIVAEETPNAAPVQEEGESM
KEKALDSVDDKSKETDKEKDTGSIEKNSVDVEKKTVEASDEKKNSEAETRNHEENGLTT
EAEGKEKTAEGEATG

>AtMBD12
MVQCTDCKKWRLIPSMQHYNIIKETQLQTPFVCGTTSGWTPNMSCNVPQDGTTCDTWPS
IPPIPTGWSRSVHIRSESTKFADVYYFPPSGERLRSSAEVQSFLDNHPEYVREGVNRSQFSF
QIPKPLDDNYVKKRTRPVKRRKSSKDNNCEKGKK

>AtMBD13
MNGEGISDGLSAERKVEIRVRKNGRKDKVIVEKSAAQGLPEGWIKKLEITNRSGRKTRRD
PFFIDPKSEYIFQSFKDASRYVETGNIGHYARKLKESDIEDDDSGNGKTVLRLEYVDKRSA
DDVLEKEKTIDDVRRSKRRNLSSSDEHSKNCKMTSDLSIVTSQVLEDLGKKEEVKDPIEK
QLIAKRVTRSQTKASTTEEVVVDLKRNLSSSNAKSEKDSVNSSVRSQKPKKEAVMKEEEE
QDSSEKRITRSKVEEKKNELSNSVARRTSKRLAGIELEPTPELKTRAKVQRIVPLDDEPTPE
LKTRTKVQRVVPPDDEPTPELKTRTKIQRIVPPDDEPTLELKTRTKVQRILPPDDELTPELKS
RTKVQRIVPPDDELTPEFKTRTKVQQRIPPDDGRAGKCKQPVNHVTTSGSKKTEIPLNKEV
AQSCNEQSSQKPHAAAATSNNRVSADSAVGIQNIGKAVGRKPSKDKKTLKSPLIVYELNPV
FHLDGYKQKEEMSPVSPLSCQTSATKCEKTAAGKRVGRSSPKANLTTSVKPTQISPLRSPN
KGKQPHPSDSGSAIQRRNKLANEYSNSSVVRGTCSEVMEKSTNSFSSAFDSTLADLCKDP
CIAFAIKTLTGESLCLLNTPAISSNPINNHTKQKGVSFTPETPGNVNTCSEKLVFPSPPPGANI
WQDPCIDFAIKTLTGAIPIGLDEPDTKSKSQGMTSTTAATQEAKGRQNNCDYMTNKTVGK
PDDLRFTQSFSKD

>StMBD1
MAKDSPKTPKTSSSRSATNPSVSVWAAQCEKCLKWRRIATQEEFEEIRSRFTEEPFNCHN
QPNASCDDPTDIEYDSSRTWAIDKPNLPKTPSGFKRELYLRRDYSKMDAYYFTPSGKKLR
SFTEVTTFLQQNPDFSDVKPSDFSFTSPKVMIDTIPSTALLANSHKKGAASR

>StMBD2
MERDHLDLASPGKADHNDIYGSSVASRRDNMKHEPKDSRSSARSGGIGTSNQNPRDVAA

LAEEEEHNTDDEDNHSEDAQKQLVLYNPAAVGADEIALDADPVNSQPRRNSFPNYASRILP
SVGAFTVQCANCFKWRLIPTKEKYEEIREHILEQPFYCETAHEWRAELSCDDPPDLTQDG
SRLWAIDKPNIAQPPPGWERLLRIRGEGGTRFADVYYVAPSGKRLRSMVEVEKYLQEHPD
YVAQGVSMSQFSFQIPRPLQDNYVKKRPYRPALAPDETEPVNRITWIADNGDTDLRLGMP
GPPLFEPLSQSFKKKRTPSKRMSNADAACKSS

>StMBD3
MTKADLAISSIPWIIVTSDDFCNDGMPSDLLFPSGTYIDVELDVRVETTTPNEGDISIEG
KVSDVILSKLQIQRKIMPICRVKLDRPKWLLENWRFETKVSITGATAGIFDRYYIETVSS
DKFRSKNEYSMTKTRGDIVKLSVPKASKNKKRNAEEVSRSSKKKKIVVEEPKSNSDSDTM
TEIDNYEDSSAHASDDV

>StMBD4
MASPMEKGSHDDEIVSVELAAPPSWKKLFTPKQGGTPKKSEVVFITPTGEEVKNRKQLEQ
YLKAHPGNPAIAEFDWSTGETPRRSARISEKVKAKRPPSLLESPKKKRRTSSGTKKVSKE
KDAAKAEKESSETKGMESSKEENENLGKKAGEAEGEMEDEGKKEVEAVVEDERIEDAKL
PPAEKPDSESEEFHSADDGNQDKSEHAEAEMEDKKKKEVEATEEDECLKGAELPSGDKPE
PESEEVHSADVGKQNKSENADIEEKQVSEEKLVSQNKPEELAAEGTNVTIGGSANQAEHA
VDGVSKDAHSNDSEDRPKEEKETEGTDLAMENNNINQPGLVHSQQHQSPAPISC

>StMBD5
MGKKRGLPNWVPNGWKVEVRTRKKGKKEKWYIDPSKGLKFCSKADVLHYLSSVDSNC
SKSSNTKEMEKKGTEKCFTEKTASEGLPPGWIKELKVRRKGRKIRKDPYYIDPVSGQTFRS
MKQVSRFLETREPGRFESTPDNRCPDTVKLEEPSSSFPAEADKQMSLDSDASGKEVNSNQ
KLKLGAEHMTHSSCVEGGMSSCEGLGDAVAGENVGKVVSDPASEERQEKDDDGEVVSN
TASGEHQEKEDDDKAISDPASREHQGKEEDGKVVSATASGEHQENEDDGKVVSETVSGE
DQEKPSRDGAEKHDQKTFQRKRKKGMNLPRRASKRLAGIKADTSLKLQTNDQTQLTSVR
QQERTQAATSDNPLNFINTSKHVATTVTGITISKKVETGSLTIKRKKRLVASSQTEREKKPSP
AEPAKTVLASCEGDKKGETALESSLSDLWTDPCIEFAIKTLTGTIPVINEKKVDEIPGSNSSK
PSVNTPSSVGLPSDEIWADPCFMFAVKTLTGEIPIGDDLCSQKIVQQQCTSSQFQGNNGLVL
PNIRFEEFGQANLPRYAAPEMSSYKQQPAVTPNAYQNRWFAKFGSHRPSPLC

>StMBD6
MTEAAAVSSIPPATVTGDDFRDESLPRDPLLQSGTYIDAESNVNTTPKKGVRPDEGNVPD
LTPAPAEGTQAIQTTETVAGTVTPLRYGSRSAEVNVERPTWLPESWRFEAKVRTSGATAG
TVDRYYYEPVSGSKFRSKTEVLYFLETGGKRKKAITGSGTDATPSETPPIKKQKKSISKT
KKVTSFYFDSGNPPQSVCWVQTDTSADTWTPSCNGSVVPGTRKQEWDAVFLSVSKLKRR
NTQTGR

>StMBD7
MEMENKDVAMEKTDAVPIELPAPPGWKKRFTPGKSSTPRRNDIIFVSPDGDEIKNKRQLDK
YLKSHPGGPPASEFNWGTGDTPRRSTRLGGKSKATETPETDTPSTKRQRKSSSKKEAKED
GGAREAEGATEKEAKASDEPALPDAEDLEVQDVEMASKNLTDGDNIKDEKEKTNDGEIV
PKEPQASEDKMEIVHEKEETKDEKEKISDGEIVPEEPLVSEDKMEIVQEKEETKDEEEKTN

ACEIVPEEPPESEDKMEIVQEKEETKDEEEKTNAGEIVPEEPPESEDKMEIVQEKEETKDEE
EKTNAGEIVPEEPPESEDKMEIVQEKEETKDEEEKTNAGEIVPEEPPESEDKMEIVQEKEET
LDEGNIEKMKNREAEDKPLDNSLKTDNKTLPLNVFEENKIESKPAGTDASSLSAELSKASS
GSPEEIAEEAPAAEDALDVVDENKIESKPAGADASSHSAELSKATSVSQEEAPAAADPLVQ
NSNDGKVHENEQIDREIPIEEIIGTESAAAGVNSSVQQ

>StMBD8
MAKSVEKNEVVSIELPAPSGWSKKFLPKKGGTPKKNDIVFTAPTGEEITTRKQLEQYLKSH
PGGPPVAEFDWGTGETPRRSARITGKAKATQSPTESESAKKRGRKSSSSKKDSKDKEVPKE
TEAAKDDDMEEAEKHEKDTAAMEAEKDVEKEEDENPNETQDGESEVEKKDEIQSSEKD
VVKENLDEGQNVDNKVEDAQVEKDVEMADNVGHSKDVEEAPVVKAADGPDATKINEE
DKDVQVQEVEKVPTEEAHVEKDVKVADNDVEEEAPADKPAVSPEATKINEEGKDVQVQE
VEILPTEEAQVEKDVKMTDNIGHPDDVEETPADGPEATKINEEEKDVQIQESENKPTEEAQ
VEKDVNNVEEALADKAAEEKDVQVQEADKVDSSATEELKHQAEGKSTDDQDNLMNIEIS
KVEGEVTENGSNANEAKP

>StMBD9
MVIYDGKIVRMDEEQLNEWAIVPSTRAATLPYDLPDGWVIEEVPRTDGSMVDKYYYEPG
TGQKFRSRIAAQRYLAQMRENVPLSATLEELKENKPLSKMFKLYHHAKKSLPCKGNIVTE
NSDKSSFLSPPAKVNWVLSSPKGDAWNPFIAGTPIPDSVKQQWTKRFMLFMNGENLNAE
NSD

>StMBD10
MLGQNKYSTVGTGNSDQASLSRKLSPDEIPPGWENEIPPGWKYEIPAQNKDPIVGTTNSD
QASLSREKASLDEIPPGWEKEILAYNKDPAIRTGNSDQASLSRELASADELPPGWEKEMLT
RNKYPTVGTGNFNSLDDIPPGWEKELLVQQKDHTVGTGNTYQSFLSKEASVDGLPPGWK
TEFRMRKNARVIKKDPYYMDPVLGYVFRSKKDVMRYLQTGDIRSCAVKPTKRDPGSTMK
DSSPSTHDTSSKKLRCSLAGIQLSATDESKGSKCYVTCSMALQAENSSEHSKSSMFNLNTS
ISSIVADITEKHSYENVNEDTEIRLDVEPEIRDSVGDTKAAAAETVDVRRSLVVSPQLDFLP
EQQLPENEKEKQPARLRKFRNSKSSTPVRRVSKRLSRHNPEMVTDLNLGERVLQTVVNNS
ALSGLPMNVSGGESAQQTDLATGDSDQPSLSRKASSRNDPLKVVKCLSEGQAFNEQNDE
RILQDSQPAGISYERGKVHLEQHALNERPTAKLAKEEQDGQNRLLNNSQLAESASGRRVF
PAEDRSVSERSTVEQVSEKRNGEYEQWQYSQLAEPSWRSVDLNVENQQWQDVQLAPSH
RFVDLNMENQQWQDSQVAPSQRSVDLNVENQQWQDSQLPESSHRNLDLNVESQPVKEK
QTGELVTENQDEQNRRLHAQLASYPFGDYWSDPCMEFAFKTLTGALPVEDTLAFQGSTH
QEYNTSYTQADDGCFELPLFNTSSFYLNDAPNHCAPSEEHVVKEQPALNQTFLPTGNNSIP
GHSSVVSQNPGLTFLPNGNNSIPGCSSIVSQNPGLNPQAKDYQSKFKSHR

>StMBD11
MLKKGVRAKKNEVVFVAPTGEEIRNKRQLEKYLKTHDGNPGMSEFDWTTGEAPRRSARI
SQKVKAMPLPAVLEPAKKRQRTSYATKKEEEMDIADIEKENMESAIEEKEGLEKEENIVEV
EMEDKGGEMGANKENTEHKKEEEMAGTEVSDRKTEASNDIQFEDGGDKMAENVTEETI
AIDADFWNDSMDKDYFKGVFANAVIGETNGAEAGIEVGEKPGYGENLESQIDIDISGADN
MNHDIPDKGTPERKVAGGETYNIQDPAFVGEASGFREEPNSVEEEKNKNRAGLVMDNCQI

NQPERAHTPQHQSVATISC

>StMBD12
MDQNVSHGVEPVNNMKPPTTPMALVVECARCFKWRYIPTKEKYEEIREHILERPFYCETT
REWHSNKQCDDPPDLTEDESGLKWAIDTPNIPQPPPGWERVIKFRTKRNTRIADVYYNSP
SYQQLRSKIEVEKYLEQHPEYATQSAKLDQFSFQIPRLLEEDYVVNGEWYKDREISSINE
LKWKRKGSKRKKRDEWGTIVLFLKKIQASNEQTVAMTSVS

>StMBD13
MDQNVSHAVEPVNNMKPPTTPGALVVECARCFKWRYIPTKEKYEEIREHILERPFYCETT
REWHSNKKCDDPPDLTEDESGLKWAIDVPSIPQPPPGYYNSPSNQQLRSKIEVEKYLEQH
LEDATQSAKLDQFSFQIPRLLEEDYVVNGGMNLY

>StMBD14
MTKADATVSSIPLTVVTSDDFHNDGIPLDPLLPLGSYIDVELDVRVETTTPNNSDVPNEG
KVSDVNRSKLQIKRKIIPMCQVNPDRPEWLPNNWTFETIVRTSGASAGQKDSYYFEPVSG
SKFRSKPEVNHFLKTGLKREKLDPNRDDATPSEGKKQKKSGSKKEKKNVD

>StMBD15
MEPAVAVAGETVQERPSWLPEDWKFKSVVRMGGATAGLTDHYYYEPLSGKRFRSKIAVLD
FLKTGTKRKKGDSETPKAKNKEEDGTSFFFDSANPPESVCWTLTDSNEDTWKPSIDGDMI
SEMEKQQWDVVFSSVSKLEISNTKNEK

>OsMBD701
MGNSKTPQPSKKSRIMLSDTDGHQLDNDEFSSESASNQMVLFNPETVAKGQDELGENHSP
SLQKSANNPNRGMPSIGAFTVQCAKCFKWRLIPTKEKYEEIRECIIQEPFECERAREWRPD
VTCNDPEDISQDGSRLWAIDKPNIALPPPGWERQIRIRGEGGTKFADVYYTSPTGRKLRSLV
EIDRYLLENPDYVAQGVTLTQFSFQIPRPLRQDYVKKRPKIVNPNDEASVVTTKSVKPEEV
SPIAWAAPSVHQEGEAGERASHADEPPEAEELELTRKRKAESPLFEEAHSNHVSDEPKTKL
EDTQNGGPSA

>OsMBD703
MRTFQGHNPNTMKVHSRKSQPSKKKPREFYDTVEVHIIDDDSDGDANIHKDYSMEDTSK
HLVMYNPEITYDKQGEVEVTEPIDNYTSLNQRYMKPRHGYNTVLPSIGAYTVQCAHCFK
WRIIPTKEKYEEIRENICQDVFVCERAREWNRVISCVDPEDISQDGSRVWAIDKASISQTPP
GWDREVRIRGEGCSKFADVYYTSPTGKKLRSLVEVGRYLAENPHYIRQGVNLTQFSFATP
KPLQEDYVRKHTYAATPELPELLETAQVDPLCWAAPPTRSELLGELGASTSRSVDVSQSEV
SYPVDLHQPEESAPLPPRTKKKTMKRGRVSATGSQSTPPGSSKDQSGGCVSDVEFVSL

>OsMBD704
MAGPSVWKGLGVAGSTSIVISDDEKKEIQQDVEDLEEEEERPGWLPDGWIMEVYQGDDG
TIYRYYTSPISGLTFTMKSEVLQYLFSGMDERFLESKNCAADNQLINNSVYVSPTMTYMQ
MTHEWLPKGWIIEVRAGGKNMNKMYKFYVYPPAGVRLFSKEDVLLYINKSEITGFDTNG
ECDTRTKDNILANVEFNPHSLPEGWVKEVVFRKTKTGVIRKDPYFTDPVNNYSFRTRKSA

MLYVQTGKVPKRAFIQRTSVHDLYSFEKSADLHESLKKRLDFAARTNRKSRRSLKSKNSS
LTEKSLSDEESSYKYGDSSDDLSDSSSEVKKNKGKLEKTTCKTKKSVSFNTAKRPVGRPSK
RSTEEMPRDVEIKQESASSEEYWC

>OsMBD705
MSTPAGVAPRRSSRLASSAPPLGAGQPSSSPLPRRRRRRASPSGCAREVDNSPPPVVILDDN
EMEQHVEAEEMKKQGEESVEANEAEEKDKDAEVLEELPDWLPDGWIMEVRCGDNGNIY
RYYTSPVSGYTFSTKMETLHYLFSEMDERVLESQACADDNELHRMHTWLPDGWAIEVRA
GGKKMEKMYKFYVHLPTGMRFLSKENVLLYSNEGKISRCDVKGLCDTSSEDNILAMVEF
NPDGLPEGWVKEIIFRKCNDGIRKDPYYTDPVSRHVFRTLKSVINYLETGQITKHAYIPRRS
VTDMYSFDRCTDLPQSMLKRLKIQGKAKKKSVGASVKGKKLSNGLASNNCMSSGLDPEI
GPEERKLGTVKSITKEAVNSDTIKRSRGRPPKILMPTIESTKPEIALVTSEAIKRSRAEGVNS
CLIHLSEPNEKMVKATSAVEPASSNNAKRHGGSPQKKFKHITDITLDCAKSSNKESEHIVTA
KKLGIGGGEQVANENTLEHTNMKEHLGVIQDYTSNRKKDKLNLITDPDLHEHKNGKFTE
KLACTAVHKFYMRRSSNHTVALKKG

>OsMBD706
MYAVQCCECHKWRKVPTKDEFETIRENFTEEPWHCSRRPDCSCEDPADIEYDSSRIWVLD
KPNIPKPPAGTERLVIMRGDLSKMDTYYVMPNGKRVRCTAEVDKFLEANPQYKDRFSVES
FSFTTPKIVEETVSHNSVWKSGKAKKQDKINALSNNN

>OsMBD707
MATAGDEQQQQAAAAQTAEVTEAAAKEVVSVEMPAPEGWTKKFTPQRGGRFEIVFVSPT
GEEIKNKRQLSQYLKAHPGGPASSEFDWGTGDTPRRSARISEKVKAFDSPEGEKIPKRSRN
SSGRKGKQEKKEATENEEAKDAEADKEAPSEDAPKETDVETKPAEEAKEAPSEDAPKDT
DVEMKTAEDASKTADADTPAPAPAGTEKEDAKPAESEAAPPAPSEGGEKKEDAKPAEPEA
AAAPPSNPTEPSAPKAAAAAPVENSADKGPHQDSQPPSAAAPAKESSPVNNGQLPAGASA
VKCT

>OsMBD708
MGTEVAPMVDMRALSQSDLVALAAGSPYSADPRRGRDADVLPPPKIDRAVFNESAGSRK
QTFSRHRVATNLSHSLTPATASAAAAPAPAPADEDSENRLIAFHLQRLFAGEDPSFASPPQIA
PQPQPQTLITPAIAAAVTPAPSLPTPPPSNADMEVMNPNGVAVDLARLAELVDPYEEEMRR
RTAGLGAESELLGFMNGLEGQWGSRRRRRKFVDASMFGDHLPRGWKLLLGLKRKERVA
WINCRRYVSPSGHQFATCKEVSTYLMSLLGYVEAKPTAIQSSDAEVLELNAVNSVGHCQP
NSTEEKQSAPPVTSVPFSSHHGDPQRQLDKNETQVEANGKECQKCNLTFQDQSAYVQHQ
LSFHQRKAKRRKVNKSGEVGANKNVTIVTQECHITSEDKLGNIDHSLATTKSQGQTPEKM
PDETISGELGGRPSMAPEPVGFQETNGLTEQGKESSAGELLSGHCDPLHNMAGVPEKEKG
SAGEPVTGNHEDPIDNFSDHKIHDGACHNAEEPHAVEAASEFNIGNSANLQQTDSTKDLV
LSNADCTQNDNITKDLAPNPTIPQGESKCIDDPMECTDMKPSKKVSEPCDLLDDKFSSFPE
GANFNGQEENSPLSAALNEPDLNSIDMEVDNDNVECKYGNAGDSTSPENGKHIEDQIIDC
RMTALKDHEINTDVRIRDVNLNSCLDAMSPPVSGANYETSNAIDDNNRSSIIAQCFGANSA
DDNACKEENFVNNQSSVSKAESFNQNNDMIYQPNLTMDPISPAQINVDCFTSCSMTSEIKN
NSNRREDNAKEQLVNPRNITSNDAGFDVEAYSNIFNGAITESSLAQLNNAINMKADYSSC

YSLSDLNTLTGGPATDEIDIHSMRNSFVNSSTSRNEPNEHCTLDFDIKGSMLEALEKSDSDL
ENQYNGSTRPCGSLPTAGTSGSIDDFMSLQTNFGSLTSLVRSVEDGPMSRIIQDQCDLQLGF
GVQKPQMYPTFEEQLRMASAGAPQFGTMNRHNHVPVPEPTLMLGYAPHIGSCPPVQLG
WDMSMSKMVGGCVLQSSMCVWCNTQFQHFGTVADQQADSLGFICPACKEKISGHLSML
NNSSSQL

>OsMBD709
METEVFPVVDLRVLSQSDLEALAAASAHAVAPGGSCPDADQLPPLKIDRAVFNESAGSRK
QTFSRVRFGAAAAVAASPSSPSPSAAAKLPRGNDKEDSFIAYHLRRLFAPDDPSSPQIQTLA
LPAPPSPDPDQLTTNSKGVSVDLVSLSRLADPYDAELGKRTAGMTTEEELMGFISSLAGQW
VSQRMRRKLVDASFFGDHLPSGWRLQLGIKRKDRKAWVNCFSYVSPKGQSFATCQEVSA
YLMSLLGYPEFKTDNIEYGSTQQHGLCADDGVNVLGVQHQIGTGMDSQSILPVASITFSSH
SRDQDETDADDINSYECQQCNLTFHGQSAYAHHLITFHKMGSKRRKINKVGKFGEPVIGK
DGKFECPVCNKTFEEQSRYFGHVGSHAKYHGLTPEAFLQTLSGKVGNDSFAGLSCSLQDL
VGSPQLNEKTTASEARSQHHNCSTKHGGNSTRGIDLFNSNCPANFNGHNQTWCRPDEIPP
TTDAPSTWTYRNNVTNCADRTVPRTAPQPNDHMDCRVSGFAEATNFNDQAGRHQGFRPS
SFGTTNHCQGQIIDHAVAASKHAEVNNSMKSRDVNLNSRLNTISFPIATANNETSTALNDV
NRSCITGKGFSGSFSNNDGAASIVLPSSGLNNKIPSSLGVADRSSIAARSFNAGYVNENGAS
EANNIGNKNNTMVYQTSLAMRPVSPCDLQLGFSGQKQQILPGYGELRPAASGSPQLGGM
AANSSIPTRPSQPQFGSMARTDALPTGPSQPGSLARPNFVPTGFSQFASRPPTSVPPADSSQF
AGGMARQNIPTTSEPTLVLGYTPQMVNGPPAQLGWDLSLSRMVSEGMLPVLCIWCNSQF
HHFGPIDAQQSGSFGFICPACKEKMSGNPNAPNNGPWQP

>OsMBD710
MVSRPLDFRTIDIRLAMGAYYGSWEAFLEDVQEVIRNLHTAFGDRPDVLEMVVALSQSFE
SLYKTEVLDLVEKFDKYLSDKNAGSEMHEELHDILTAANSLPKAPWEDGVCKVCGIDRD
DDSVLLCDKCDSEYHTYCLNPPLARIPEGNWYCPSCMLGQTKAHHDQGVQDVKRQQKK
FVGEEAHAFQEELNKLATAMEEKEYWDLNMQERIYLLKFLCDEMLNTALIREHLDQCSD
KLGDLQQKFRASNFELKDLKYKEEMRTSYARQSRSSKTEQHFNNSSGPVENQQQCTPTAL
DHLEEAEQGNVGVNLNNPADGVPDGQLNVGKPYKSDKDISSASMVEERKSSGLSEQPSG
MAIDQIDGDAIDEGSQSCEKRSLGAKSSTCDNLNLKDTEFSTPGRELPDERASTSFQDNLE
ASSTKSIELDADNNEMDTLSDDISKLQDSISLLESQINMASSRRECLGKDSIGRLYWVIGRP
GKRPWLVADGSMLKPKERDISMVNSYPPSAFDCKGWNSASIFIYESDEEIQCLLDWLRDY
DPREKELKDSILQWQRHFCHQSSSPLVDPPISGPKGEQLMELPNTKAAVILEQKYGLQLDQ
DTSDLPKKRGKKIKLSSEDRTYRCDCLEPVWPSRYHCLTCHETYLISTEFEGHNDGKCSKI
HQSPDESRENDEPKVKVTKSDTKEKDSLECSSVIEPSSDRKLMQCPYDFEEICRKFVTNDS
NKETVKQIGLNGSNGVPSFVPSPAFFLEPAIVQSQNRKDDELKDWTSSLEECNAMSAQKL
VQEVSKSGQSCPGNVGDEKVQKSKKPTPDNTSGEEAHSTTGKPTRLLAVNGGLVPESSLR
PLIGRNSHILKQQKINLLDIEAALPEEALRASKCQQIRRRSWRAFVKDAESISQMVLAANL
LEGMIKAEFLKNDWWYWSSFTAAMKTSTVSSLALRVYTLDDCIIYSKDQVPSVEPADNTR
SGNRGGRRRRELESLAS

>OsMBD711
MALEGKPGFITMYAITCCKCEKWRTIPTKEEFEVIRENYPAKPWFCSKKRDCSCEHPEDIQ

YDTSRIWAIDRPNIPKPPPKTERLLIMRNDLSKMDAYYVLPNGKRAKGKPDIDRFLKENPE
YAATLPLSSFNFSTPKIVKETVSDSAKWVMAKSEREEQCMQLDAKEVPSSSSK

>OsMBD713
MGKSPSFGILDDNDCDEVKSSDESTKNLQSDDDDVDDRQYYICPVSGRTFTMKSEVLHYL
FSEMDQCFTESKNRAVGSNLTRTHEWLPKGWLVEIRAGGDNMDKMYKFYVYPPNRVRLF
SKDDVLLYIKEMKISGFDTDGQCNTSTQENILAILEFNPEGLPQGWVKEVVFRKTHTGRIR
RDRHYTDPIKSYVFRTKRSAAFYVETGKVTIRAFVQKTSVHEHESLQKRLNLGRTNQLRT
RSSKLQKLSLKEGILSDDQSSSSAARCRQQPEWVWVSFSFRFSVDRFPVSGALSYLGSAD
VVSTLWRGGSTAGDGLLVVFCAVVAWRELLWWRYWWWRVWVELATQS

>OsMBD714
MGKSPSFGILDDNDCDEVESSEEATKNLQSDDDDVDDRPGWLPDGWIMEVYRGDNGTIY
QYYICPVSGSTFTMKSEVLHYLFSEMDQRFMESKNCAVGCNLMRTHEWLPKGWLVEIRA
GGDNMDKMYKFYVYPPHRVRLYSKDDVLLYIKEMKISGFDTDGQCNTSTQENILAILEFN
PKGLPQGWVKEVVFRKTNTGGIRRDRHYTDPIKNYVFRTMRSAALYVETGKVTIRAFVQ
KTSVHEVYSFEKFTHLVIVLQNFSHFMLVLLWIK

>OsMBD715
METEVFPVVDLRVLTQSDLDELAAASAHAVDPRSSCPDRAVFNESAGSRKQTFSRVRFVPA
AAAAAAAASASAAAAAKLPRGNDKEDSFIAYHLRRLFAPDDPSLIENPSFPQTQTLARSPS
PDPDQLTTNSRGVSVDLVSLSRLADPYDAELGKRTARMTTEEELMGFISSLAGQWVNQR
MRRKLVDASFFGDHLPSGWRLQLGIERKDRKAWVNCFSYVSPKGQSFATCQEVSAYLMS
LLGYPEFKTDNIEYGSTQQHGLCADDGVNVLGVQHQIGTSMDSQSNLPVASATFYSHSRD
QDETVADDINSYECQQCNLTFHGQSAYAHHLITFHKVSSKRRKSNKVSKFGEPVIGKDGKF
ECPVCNKTFEEQSRYFGHIGSHAKYQGLTPEAFLQTFSGKVGNNSFAGLSSSLQVLVGSPQ
LNEKTTACEARSQHHDCSTKHGGNSTRGIDLFNSNHPANFNGHNQSWCRSDEIPPTTEAQ
STWTYRNNEMNCADRTVPRTVPQPNDHEDCRVSGFAEATNFNDQAGRHQGFRPFSFGTT
NHCQGQIIDHAVAASKHAEVNNSMKSRDVNLNSRLNTISFPIATANNETSTALNDVNRSCI
TGKGFSGSFSNNDGAASIVLPSSGLNNKISSSLGVADRSSIAARSFNAGYVYENGASEANNI
GNKNNTMVYQTSLAMRPLSPGKQSSGVASRRTAAVVSERHGGQVVRHPRTASSVYMPEQ
GPGWL

>OsMBD717
MDYSNSSCSRTCSGLVRLNNTIESNEVLFSKTRSGLVRINSTVESNEVSLSKTRSGLIRGNT
NVRSNEGSYSTTRSELAGANGVVDSNVGDLCSKTRSGLVRRNATLDSNEGSCSKTRSGLA
RGDGIVGLNQGSSSKTRSGLVRGGDIMNFSEGSSSKTRSGLVRGNTTVAASNGSYSATPSR
FVRGNIIVGSKEGSCSRTRSGLVRESIQMDYSDSSCSRTRSGLVRRKPFMVQVKDEAAMN
GLSDDCLKEDSPGKNEPNHKSNLVENSDKPVMKGPDGWWKEGMLTKNGSKYMSDPVQ
TKGEACINGLPGGQWKENSAEKNVSNHKNELVQRKDDLIVDGLPDGWWKEDRPRKNGS
NLKTDPYYIDPVSGYEFRSLKDVHRFLKSGDIYKCIVRPRKRTIQDPCTIENQSHTATLLQH
TRPGTADKAIQCELLTSEGLMLPWEEQLSPYRELNNPKKMPELEGMIASQKHAYKVDAPR
EKKSFPRKRKQPSAGGKPKKHKIVPAKMVAMPVRASPRLASLKITHDLNTEPEDELIGVN
LVNEVQSTKENPTDKSRLNQAGISTEMTSVQERADNQLLSSQADTGNHIRVMEGDTTDSS

QLRQADTVNQILTDQENAVSQLQSSHTDYFVQIRPRQEYVTNYSQSQLSRAATVNQIETN
QRNMSGQLQSSQADSLDQIQTEQESSASRLQSSQADSFNEIHTIQEYITEQSESQVSHANQI
QIDLGNTVDLLPSSQADAIFQMETTQEYITNQSQSRQADIVDHMQVNQDNTANQFHLRQA
DTVNRIQTMQESTTDQPQLIQALTVNQIQVNGENTANHVQSRQADTVNHIQLNQDNTVN
QFQLRRADTVNRIQTMQDSTADQPHLIQALTVDQIHANRENVVNHFQSRQADTVNHIQVI
QDNTANQFQFRRADTVNKIQTMQDSTTDQPRFIQALTVNQIQANGENTANYLQPNYAEN
NIMQVGFSLTPEPEEAPATSFWRNVANQELPVSMQTDGKPVVSSALNVEYQNVPATAPAQP
TRAPHPEAASYPSGLAVPSLFGNSWSDPCIEFAFKTLRGDIPVLDDTSAVEQYFPQHDLNKP
PSPDYSASPSCFSSSFDNTRNFTQVDHASLPAPNPSDKLYNGGWFPPK

>OsMBD718
MDYSNSSCSRTCSGLVRLNNTIESNEVLFSKTRSGLVRINSTVESNEVSLSKTRSGLIRGNT
NVRSNEGSYSTTRSELAGANGVVDSNVGDLCSKTRSGLVRRNATLDSNEGSCSKTRSGLA
RGDGIVGLNQGSSSKTRSGLVRGGDIMNFSEGSSSKTRSGLVRGNTTVAASNGSYSATPSR
FVRGNIIVGSKEGSCSRTRSGLVRESIQMDYSDSSCSRTRSGLVRRKPFMVQVKDEAAMN
GLSDDCLKEDSPGKNEPNHKSNLVENSDKPVMKGPDGWWKEGMLTKNGSKYMSDPVQ
TKGEACINGLPGGQWKENSAEKNVSNHKNELVQRKDDLIVDGLPDGWWKEDRPRKNGS
NLKTDPYYIDPVSGYEFRSLKDVHRFLKSGDIYKCIVRPRKRTIQDPCTIENQSHVSSLQFF
LV

>TaMBD1
MASPAPVPASPGSSSQKKRGATESIGLYAVQCCECHKWRTVSTKDEFETIRENFTEDPWSCS
KRPECSCEDPPDIEYDSSRIWVIDKPNIPKPPPKTERLVIMRGDLSKMDIYYVLPNGKRARG
IGDVQKFLDTNPEYKDRISAESFSFTVPKIVEKTVSQSSLWKTKKAKKQDKINASSSKKDK
ANASSSEN

>TaMBD2
MDSSKSPQPLKKSRTSLSGTDGHQFENDELPSETASDKTPGLKFETVDKVQDEFGEDSSPL
QQSAASNVSYRGSPCIGAFTIQCARCFKWRLIPTKEKYEEIREHIIQEPFDCERAREWKPDV
TCDDQEDISQDGSRLWAIDKPNIAQPPAGWERQIRIRGGGGTKFADVYYTSPTSRKLRSLV
EVDRYLQENPEYGAQGVTLAQFSFQIPRPLRQNYVKKRPKNASPSDEATTKPVQPVEVNPI
SWAAPLASEAKASEPASHADEKPVGSADVELVRKRKAEPGEANANNHVSDGPETKVEDA
QNGDATTTA

>TaMBD3
MVETKREPAGEGLPDGWLKEYRPRKPRPGSRFRRDKFYIDPTNSYEFRSLKEAHHYLESQ
DTSDSVVTPKKKKIEDLQVSGNKSQHAGRPSSEPEGVSKGRLADLELQVARKNDQRLNH
ESAAREEANVEPKPKGKKQKTEPVKQIAAPVRSSPRLTALKRNEEANMYLETHLWTTDRY
R

>TaMBD4
MDKPGIPRPPPATKRLVIMRRDLSKMDTYYLLPNGKRARSGNDVEKFLQENPEYRANLPA
SKFSFATPKIVPATIGESSLWRVAQAEGEKFEEMDVFGF

>TaMBD5
MDKAGIPCPPPVTERLVIMRRDLSKMDTYYLLPNGKRVRSGGDVEKFLQENPEYRVNLPA
SKFSFAMPKTVPATVVESSLRRVAKAEGKV

>TaMBD6
MATGGDRAAEELVSVEMPAPEGWTKKFTPQSRGRSEIVFVSPTGEEIKNKRQLNSYLKAN
PGGPTSSEFDWSTGDTPRRSARISEKVKVFDSPEGEKIPKRSRNSSGRKGKQEKKEDPETE
EDKEAEAGTEAPSEDVAKGTDVEMTIAEAIDAAKSTDVEMKPAEANDAAKNADVEMKV
AEEVKACS
