# Supplementary material for: Exploration of the Potential Transcriptional Regulatory Mechanisms of DNA Methyltransferases and MBD Genes in Petunia Anther Development and Multi-Stress Responses
Source: Genes (Basel). 2022 Feb 8;13(2):314. doi: 10.3390/genes13020314 (PMC8872020; doi:10.3390/genes13020314)
Supplement: Supplementary file 1 [file genes-13-00314-s001.zip › Table S3.pdf]

Table S3 Primers of *PhC5-MTase and PhMBD* genes used in the experiments.

		Primer sequence(5'→3')	
Gene number	Gene name	Forward	Reverse
Peaxi162Scf00326g00710.1	*PhDRM2*	GGGCAGCAACAGGGTGAG	CGAGGATAGTGATAAAACAAGGAAG
Peaxi162Scf00441g00015.1	*PhCMT1*	ATGCCGAGAAAACGAAAAGC	TGTCGCTTTGATTTCCTAGTTGT
Peaxi162Scf00164g00518.1	*PhDRM3*	TCCACCAGTGCCAGTTATTCA	CAAGGATGCGTTCTATTTGTTCT
Peaxi162Scf01053g00039.1	*PhMET1*	GAGGTGGTTGGTTGTCAGGTG	TCCCCATCCCAGTTTATTTCTT
Peaxi162Scf00062g00201.1	*PhCMT3*	TAGATTTGTATTCGGGATGTGGA	GCAAAAGGGCTAAGGTCAACG
Peaxi162Scf00045g00047.1	*PhCMT2*	TGTTTCCAAAGTCAATGTGGTAGA	CCAAGGCTCAACCCCGTA
Peaxi162Scf01039g00228.1	*PhMBD1*	CACAATGTGGGAACTGCTTCA	GGGTTTTAGGTTGATTTGGCTTA
Peaxi162Scf00320g00413.1	*PhMBD2*	CCGAGCCACATGCTTTGA	TCTGATTAGCTTGTTCCCTTGC
Peaxi162Scf00915g00037.1	*PhMBD3*	ACCACTAAAGGAGCGAGAACTG	GGCATACCCAGACAAGGACC
Peaxi162Scf00313g00818.1	*PhMBD10*	CTGAAGAAACTGAACCAGCAAAG	TCAGTTGCTTGGGCTTCCTC
