# Supplementary material for: Exploration of the Potential Transcriptional Regulatory Mechanisms of DNA Methyltransferases and MBD Genes in Petunia Anther Development and Multi-Stress Responses
Source: Genes (Basel). 2022 Feb 8;13(2):314. doi: 10.3390/genes13020314 (PMC8872020; doi:10.3390/genes13020314)
Supplement: Supplementary file 1 [file genes-13-00314-s001.zip › Table S5.pdf]

Table S5. Nomenclature and physicochemical properties of *PhC5-MTases*.

PhC5-MTase number	Protein ID	theoretical isoelectric points	Protein length (aa)	molecular weights
PhMET1	Peaxi162Scf01053g00039.1	5.88	1557	174.83957
PhCMT2	Peaxi162Scf00045g00047.1	6.32	1022	113.98887
PhCMT3	Peaxi162Scf00062g00201.1	5.26	892	100.55967
PhCMT1	Peaxi162Scf00441g00015.1	5.08	786	88.47439
PhDRM3	Peaxi162Scf00164g00518.1	5.75	662	74.12327
PhDRM2	Peaxi162Scf00326g00710.1	4.8	604	68.48901
PhDRM4	Peaxi162Scf00005g00086.1	4.96	602	67.69436
PhDRM1	Peaxi162Scf00089g00638.1	5.32	553	62.94315
PhDNMT2	Peaxi162Scf00341g00624.1	6.26	397	45.2213
