# Supplementary material for: Exploration of the Potential Transcriptional Regulatory Mechanisms of DNA Methyltransferases and MBD Genes in Petunia Anther Development and Multi-Stress Responses
Source: Genes (Basel). 2022 Feb 8;13(2):314. doi: 10.3390/genes13020314 (PMC8872020; doi:10.3390/genes13020314)
Supplement: Supplementary file 1 [file genes-13-00314-s001.zip › Table S6.pdf]

Table S6. Nomenclature and physicochemical properties of *PhMBD* genes.

MBD number	Protein ID	theoretical isoelectric points	Protein length (aa)	molecular weights
PhMBD4	Peaxi162Scf00714g00124.1	8.71	1948	213.81139
PhMBD7	Peaxi162Scf00411g00117.1	9.92	220	25.2789
PhMBD5	Peaxi162Scf00224g00513.1	5.57	246	27.54061
PhMBD1	Peaxi162Scf01039g00228.1	8.54	167	19.10753
PhMBD9	Peaxi162Scf00332g00710.1	4.93	336	37.58765
PhMBD2	Peaxi162Scf00320g00413.1	6.02	323	36.1113
PhMBD10	Peaxi162Scf00313g00818.1	4.43	368	39.98523
PhMBD3	Peaxi162Scf00915g00037.1	5.94	542	59.28419
PhMBD6	Peaxi162Scf00954g00319.1	5.96	334	37.39162
PhMBD8	Peaxi162Scf00207g00645.1	4.63	312	34.50802
PhMBD11	Peaxi162Scf00392g00416.1	4.94	1190	130.347
