# Supplementary material for: Exploration of the Potential Transcriptional Regulatory Mechanisms of DNA Methyltransferases and MBD Genes in Petunia Anther Development and Multi-Stress Responses
Source: Genes (Basel). 2022 Feb 8;13(2):314. doi: 10.3390/genes13020314 (PMC8872020; doi:10.3390/genes13020314)
Supplement: Supplementary file 1 [file genes-13-00314-s001.zip › Table S7.pdf]

Table S7. Fifteen different motifs commonly observed in *PhC5-MTase* genes.

Motif	Best possible match
1	NRNSDSPLSDVKNEQJLVFLSIVDYLRPKYVLMENVVDFVRFNKGYLFRLALASLLEMGYQVRFGIMEAGAYGLPQFRKRVFIWAALPSEVLPEWPEPTH
2	VQMCFHPEQDRVLTVRECARLQGFPDSYKFSGTIKEKYRQVGNAVAVPVAFALGY
3	ISRTDRYKSLGNSFQVDTVAYHLSVLKDLFPNGINVLSLFSGIGGAEVALHRLGIRLKNVVSVEISEVNRNILRSWWEQTNQTGELI
4	YAIMEKTGEPDDCLSTCEAKELEFKLDGNKKSTLPLPGQVDVICGGPPCQGISG
5	TSVLYDHRPLQLNEDBYIRVCQIPKRPGABFRDLPGVIV
6	ELALLDJYSGCGGMSTGLCKGAVSSGVWLITKEEPDANAFAQNSLKLNH
7	YYENEAFKAKDGWFYISVRLFYRPPDTVDEKYYAADIRKRGYIHNLPIDNPFALLPKKEVTI
8	IYFVEYMFESSDHSKMLHGKLLQRGSETVLGTAANERELFLTNECLTVQLKDIKGTVSLEIRSRPWGHQYRKENIDADKLDRARAEERKAKDLPTEYYCK
9	TDMAWYRLGKPSKQYAPWFEPVLKTVRVGISILTLLKRESRVAKLSYADVIKRLCGLEENDKAYISSKLLDVERYVVVHGQIILQLFEEYPDEDIKRCPF
10	GDDKEEEKGVRFQSFGRVENWNISGYEDGSPVIWISTALADYDCRKPSKKYKKJYDYFFEKACACVEVYKSLSK
11	VHGKSKGPFGRLWWDENVPTVVTDPZPH
12	QVQKYVLDQCRKWNLVWVGRNKLAPLEPDEVEMJLGFPKNHTRG
13	HHTKWIIKKKKKILQKGENLNPRAGIAPVVSKMKAMQATTTRLVNRIWGEFYSIY
14	DLPCRTLHNWALYNSDSRLISLELLPMKPCADIDVTIFGSGVVAEDDGS
15	SGJEILSSGYGGFDFVISQSPCIYLQLANLDST
