# Supplementary material for: Exploration of the Potential Transcriptional Regulatory Mechanisms of DNA Methyltransferases and MBD Genes in Petunia Anther Development and Multi-Stress Responses
Source: Genes (Basel). 2022 Feb 8;13(2):314. doi: 10.3390/genes13020314 (PMC8872020; doi:10.3390/genes13020314)
Supplement: Supplementary file 1 [file genes-13-00314-s001.zip › Table S8.pdf]

Table S8. Fifteen different motifs commonly observed in *PhMBD* genes.

Motif	Best possible match
1	IKKBEVYYVAPTGKRFRSKKEVZKYLKEH
2	VGAFTVQCANCFKWRLIPTKEKYEEIRGHILEEPFYCETAREW
3	CDDPPDITQDGSRLWAIDKPNIPQPPSGWERJLRI
4	ISEFDWSTGETPRRSARISEKVKAKPPPAVLESPKKRRRTSSASKK
5	EEPLPPGWKKEJRPRKKG
6	DIWADPCFEFAFKTLTGEIPIEDDFCFQGIVQQEC
7	FYSDPGKGLKFRSKRDVLRYL
8	YVRQGVQLNQFSFQIPRPLQEDYVKKR
9	LMMENGKINQPEPAHSPQQQ
10	MEGDQPGLVTPGKMDQNNNHG
11	NHGEDAQKQLVLYDPAVVG
12	CWVHEDSFADIWTDPCIEFA
13	RGLPYDVDIDPGFGSKFW
14	RNPSTPKIPMPPRFRRRWSGEEH
15	AFKGETGDFEENFGEVRH
