# Supplementary figures and images for: Exploration of the Potential Transcriptional Regulatory Mechanisms of DNA Methyltransferases and MBD Genes in Petunia Anther Development and Multi-Stress Responses
Source: Genes (Basel). 2022 Feb 8;13(2):314. doi: 10.3390/genes13020314 (PMC8872020; doi:10.3390/genes13020314)

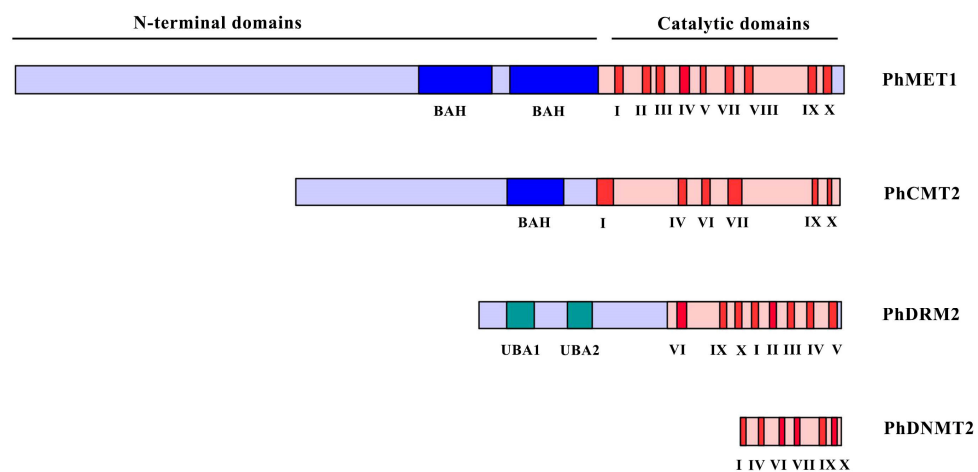

**Figure S1. Schematic structures of *PhC5-MTase* genes.**

Supplement: Supplementary file 1 [file genes-13-00314-s001.zip › Figure S1.pdf]

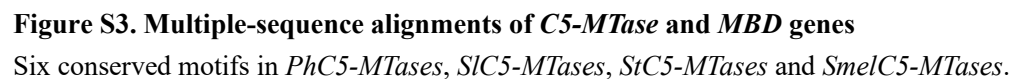

Six conserved motifs in *PhC5-MTases*, *SlC5-MTases*, *StC5-MTases* and *SmelC5-MTases*.

Supplement: Supplementary file 1 [file genes-13-00314-s001.zip › Figure S3.pdf]
